# Priming agents transiently reduce the clearance of cell-free DNA to improve liquid biopsies

**DOI:** 10.1126/science.adf2341

**Published:** 2024-01-19

**Authors:** Carmen Martin-Alonso, Shervin Tabrizi, Kan Xiong, Timothy Blewett, Sainetra Sridhar, Andjela Crnjac, Sahil Patel, Zhenyi An, Ahmet Bekdemir, Douglas Shea, Shih-Ting Wang, Sergio Rodriguez-Aponte, Christopher A. Naranjo, Justin Rhoades, Jesse D. Kirkpatrick, Heather E. Fleming, Ava P. Amini, Todd R. Golub, J. Christopher Love, Sangeeta N. Bhatia, Viktor A. Adalsteinsson

**Affiliations:** 1 Koch Institute for Integrative Cancer Research, Massachusetts Institute of Technology, Cambridge, MA 02139, USA.; 2 Harvard-MIT Division of Health Sciences and Technology, Institute for Medical Engineering and Science, Massachusetts Institute of Technology, Cambridge, MA 02139, USA.; 3 Broad Institute of MIT and Harvard, Cambridge, MA 02142, USA.; 4 Department of Radiation Oncology, Massachusetts General Hospital, Boston, MA 02114, USA.; 5 Harvard Medical School, Boston, MA 02115, USA.; 6 Division of Pulmonary and Critical Care, Department of Medicine, Massachusetts General Hospital, Boston, MA 02124, USA.; 7 Department of Biological Engineering, Massachusetts Institute of Technology, Cambridge, MA 02139, USA.; 8 Microsoft Research, Cambridge, MA 02142, USA.; 9 Department of Pediatric Oncology, Dana-Farber Cancer Institute, Boston, MA 02115, USA.; 10 Department of Chemical Engineering, Massachusetts Institute of Technology, Cambridge, MA 02139, USA.; 11 Department of Electrical Engineering and Computer Science, Massachusetts Institute of Technology, Cambridge, MA 02139, USA.; 12 Department of Medicine, Brigham and Women’s Hospital, Boston, MA 02115, USA.; 13 Wyss Institute at Harvard University, Boston, MA 02215, USA.; 14 Howard Hughes Medical Institute, Cambridge, MA 02138, USA.

## Abstract

**INTRODUCTION:**

Liquid biopsies including the analysis of cell-free DNA (cfDNA) from blood can be used to diagnose, monitor, or molecularly profile disease. Despite the fast adoption of liquid biopsies in oncology, prenatal testing, infectious disease, and organ transplant monitoring, higher sensitivity is needed in many important clinical applications. In oncology, efforts to improve the sensitivity for detecting circulating tumor DNA (ctDNA) have mostly focused on ex vivo sequencing and analysis methods. However, an intrinsic challenge is the scarcity of ctDNA in vivo, which leaves little ctDNA to be collected and analyzed.

**RATIONALE:**

We hypothesized that transiently attenuating cfDNA clearance in vivo would augment the levels of ctDNA in circulation and increase the amount recovered from a blood draw. The two natural mechanisms for clearing cfDNA are uptake by liver-resident macrophages and degradation by circulating nucleases. In this work, we sought to develop two intravenous priming agents given 1 to 2 hours before a blood draw that act on these mechanisms and enhance ctDNA recovery. Our priming agents comprise (i) nanoparticles that act on the cells responsible for cfDNA clearance and (ii) DNA-binding monoclonal antibodies (mAbs) that protect cfDNA.

**RESULTS:**

We first investigated the nanoparticle priming strategy and identified a succinyl phosphoethanolamine–based liposomal agent that inhibited cfDNA uptake in vitro and transiently increased the recovery of cfDNA from blood in healthy mice. We confirmed that liposomes rapidly accumulated in the liver and that liver resident macrophages were necessary for cfDNA half-life extension. As an orthogonal strategy, we showed that DNA-binding mAbs interacted with elements of cfDNA and protected double-stranded DNA from nuclease digestion. Engineering the mAb to abrogate Fc-γ-receptor (FcγR) binding increased its persistence Circulation and the recovery of cfDNA from blood compared with that of the native mAb and an isotype control mAb in healthy mice. Using a bespoke ctDNA assay tracking 1822 tumor-specific single-nucleotide variants (SNVs) in plasma samples from mouse preclinical cancer models, we demonstrated that our two orthogonal priming strategies increase the recovery of ctDNA by >10-fold, enable more complete tumor molecular profiling from ctDNA, and increase the sensitivity for detection of small tumors from <10% to >75%.

**CONCLUSION:**

By modulating cfDNA clearance in vivo, priming agents improved the sensitivity and robustness of ctDNA testing in tumor-bearing mice. Just as intravenous contrast agents have profoundly improved clinical imaging, we envision that priming agents will improve the sensitivity and utility of liquid biopsies across clinical applications. Additionally, the concept of delivering priming agents that transiently attenuate analyte clearance in vivo and boost diagnostic sensitivity may inform similar approaches to enhance the testing for other scarce biomarkers in oncology and beyond.

## Graphical Abstract

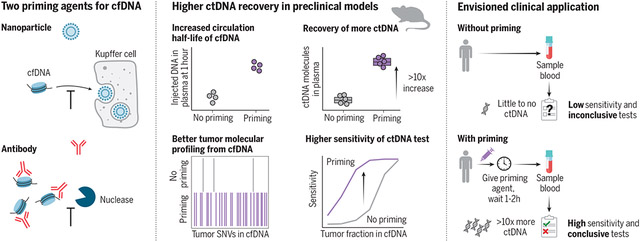

**Priming agents reduce the clearance of cfDNA and enhance the sensitivity of liquid biopsies.** Priming agents transiently attenuate natural clearance mechanisms for cfDNA and consist of nanoparticles that act on the cells responsible for cfDNA clearance (top left) or DNA-binding antibodies that protect cfDNA from cellular uptake and enzymatic digestion (bottom left). In preclinical models, priming agents increased the half-life of cfDNA, enhanced recovery of ctDNA, and improved tumor molecular profiling from ctDNA and sensitivity of ctDNA testing (middle). We envision that priming agents could be administered 1 to 2 hours prior to a blood draw in the clinic to improve the recovery of ctDNA and boost the sensitivity of many types of liquid biopsy tests (right).

Liquid biopsies such as blood draws are a source of biological analytes such as cell-free DNA (cfDNA), and enable noninvasive diagnosis, monitoring, and molecular profiling of disease (1). The number of diagnostics based on liquid biopsies has grown rapidly over the last two decades in prenatal testing (2), infectious disease (3), oncology (4), and organ transplant monitoring (5), but the sensitivity of liquid biopsies remains inadequate for many applications. For example, in oncology the sensitivity of circulating tumor DNA (ctDNA)–based screening tests is low (~20 to 40% for Stage I cancer) (6, 7), and liquid biopsies can be inconclusive in up to 40% of patients with advanced cancer (8). Additionally, up to 75% of patients who test negative for minimal residual disease after surgery experience recurrence (9-11).

To date, efforts to improve the sensitivity for detecting ctDNA have focused on sequencing and analysis (12, 13), such as tracking multiple somatic variants (9, 10, 14-16) and integrating features such as DNA methylation or fragmentation patterns (17-19). An intrinsic challenge for all these methods is the scarcity of ctDNA in the collected samples, which limits sensitivity (20, 21). One option to improve sensitivity is to draw larger volumes of blood (4) or to perform plasmapheresis (16). Large volumes, however, are impractical in frail or ill patients, and plasmapheresis carries major risks and requires expensive instrumentation. Alternatively, methods to sample more proximally to the tumor (22) or to increase tumor DNA shedding have been proposed (23, 24). These methods require prior knowledge of tumor location, are limited to specific primary tumors, and often require specialized, expensive, and invasive procedures.

To realize a generalized approach for enhancing the amount of ctDNA recovered in any blood collection, we have developed two intravenous priming agents that transiently delay cfDNA clearance in vivo (Fig. 1A). The two natural mechanisms for clearing cfDNA are uptake by liver-resident cells of the mononuclearphagocyte system (MPS) (25, 26) and degradation by circulating nucleases (27) (Fig. 1B, left). Given that the majority of cfDNA circulates while bound to histone proteins as nanoparticulate mononucleosomes (~11 nm in diameter) (1), we hypothesized that a competing nanoparticle, such as a liposome, that is efficiently phagocytosed by the cells of the MPS would attenuate cfDNA cellular clearance (Fig. 1B. middle). Although the notion of saturating MPS uptake with a nanoparticle has been explored therapeutically to decrease the hepatic accumulation of nanomedicines (25, 28-31), we have now applied this strategy to increase the abundance of an endogenous analyte for enhancing a diagnostic signal. As an orthogonal strategy, we also hypothesized that a DNA-binding priming agent could directly protect cfDNA itself from circulating DNases and extend its half-life in circulation (Fig. 1B, right). For this affinity-based approach, we selected monoclonal antibodies (mAbs) to develop given their persistence in circulation, ease of engineering, established manufacturing processes, and well-established safety and efficacy as biopharmaceuticals (32, 33). In this work, we show that both approaches to priming agents improve recovery of ctDNA by more than 10-fold, enable better molecular profiling of tumors from blood samples, and increase the sensitivity for detection of small tumors from <10 to >75% in preclinical cancer models.

## Nanoparticle priming agent attenuates cfDNA uptake by cells of the MPS

**Fig. 2.**
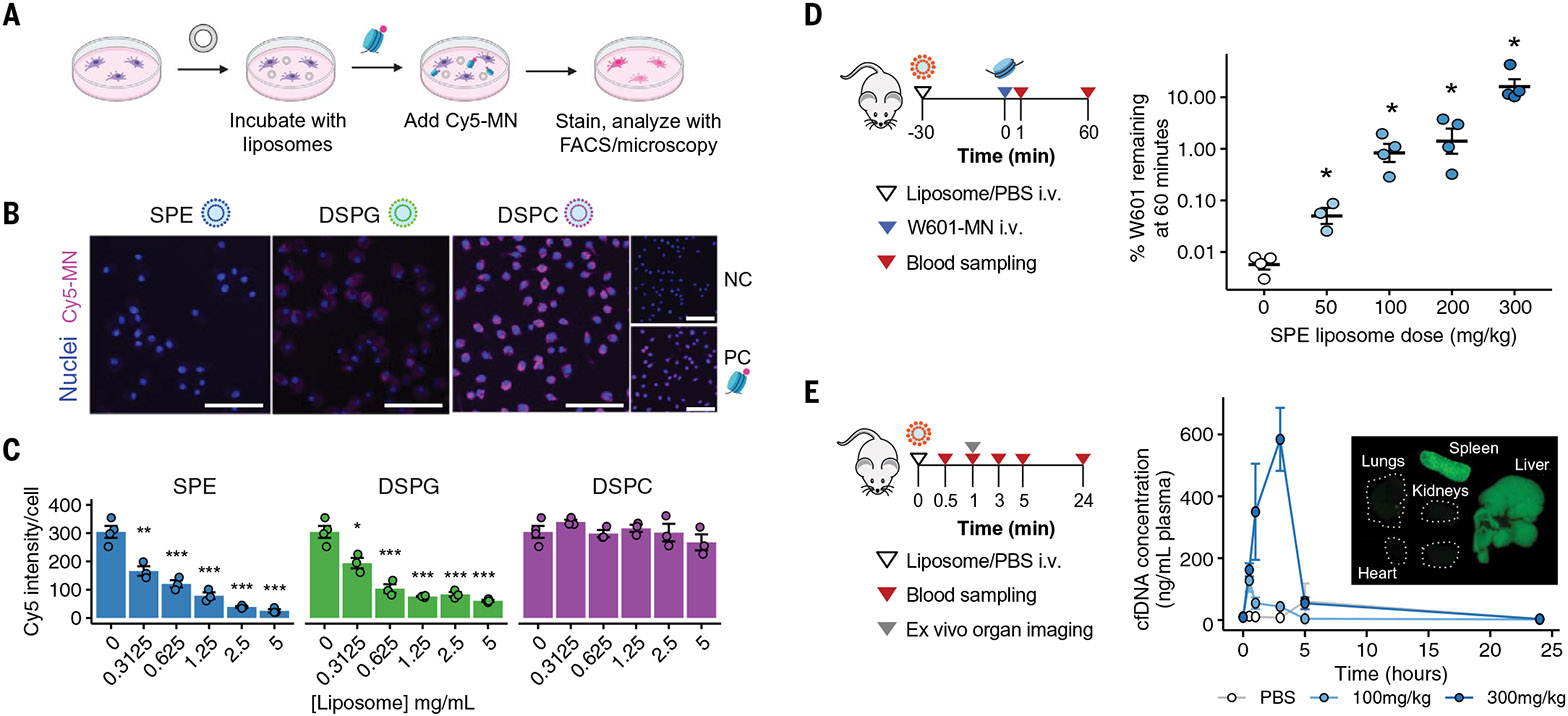
SPE liposomes inhibit the uptake of mononucleosomes by macrophages in vitro and increase the recovery of cfDNA through decreased clearance in healthy mice. (**A**) Schematic of in vitro macrophage uptake inhibition assay. (**B**) Representative images of uptake of Cy5-labeled mononucleosomes (Cy5-MN) following incubation of J774A.1 with different liposomes at 5 mg/ml, without liposomes or Cy5-MN (negative control, NC), or with Cy5-MN only (positive control, PC). Scale bars, 100 nm. (**C**) Quantification of Cy5-MN uptake by J774A.1 cells from epifluorescence images after liposome pre-treatment (mean ± SEM, *n* = 3 to 4 wells per condition, *N* = 2). *P < 0.05; **P < 0.01; ***P < 0.001; one-way ANOVA. (**D**) (Left) Experimental approach to determine the plasma bioavailability of W601-mononucleosomes (W601-MN) following SPE liposome priming. (Right) Percentage of W601 remaining in plasma 60 min after administration of different SPE liposome doses (median, *n* = 3 to 4 mice per group, *N* = 2). *P < 0.05, two-tailed Mann-Whitney test. (**E**) (Left) Experimental approach to determine plasma cfDNA yields and liposome organ biodistribution. (Right) Plasma cfDNA concentration following Cy7-SPE administration (mean ± SEM, *n* = 3 mice per group). The largest elevation relative to the PBS group was at 30 min with the dose of 100 mg/kg liposome (10.3-fold, *P = 0.034) and at 3 hours with the dose of 300 mg/kg liposome (78.0-fold, **P = 0.005) (unpaired two-tailed *t* test; *n* = 3 mice per group, *N* = 1). (Insert) Organ biodistribution of Cy7-SPE liposomes 1 hour after administration. Images from a representative mouse are shown (*n* = 4 mice, *N* = 3).

To test our hypothesis that administering liposomes inhibits cellular uptake of cfDNA, we first designed an in vitro two-dimensional assay using the murine macrophage cell line J774A.1 (Fig. 2A). Following pretreatment of J774A.1 cells with liposomes, we added Cy5-labeled mononucleosomes (fig. S1) and quantified their uptake. Empty liposomes were generated with cholesterol (50 mol%) and one of three different lipids [1,2-dipalmitoylsn-glycero-3-phosphoethanolamine-N-(succinyl) (SPE), 1,2-distearoyl-sn-glycero-3-phospho-(1'-rac-glycerol) (DSPG), or 1,2-distearoyl-sn-glycero-3-phosphocholine (DSPC) (50 mol%)] sometimes used in FDA-approved liposomal formulations (34) (figs. S2 and S3). The average hydrodynamic diameter of the liposomes was between 230 and 260 nm and designed to match the size of the fenestrae of murine liver capillaries (31) such that they would preferentially target liver-resident macrophages over hepatocytes. The SPE- and DSPG-based formulations, but not the DSPC-based one, significantly (*P* < 0.05) inhibited the uptake of mononucleosomes by macrophages in a dose-dependent manner (Fig. 2, B and C). These two formulations are more negatively charged, consistent with prior reports that negatively charged particles display increased interactions with macrophages versus neutrally charged particles (35, 36). Using SPE liposomes, we confirmed that inhibition of mononucleosome uptake was also dose dependent in the independent macrophage cell line RAW264 (fig. S4, A and B). Cell viability was not compromised with liposome treatment (fig. S4, C and D), and the liposomes did not impair phagocytosis of inactivated *Escherichia coli* in J774A.1 cells at the range of concentrations tested (fig. S5). These data suggest that our priming agent may not affect normal phagocytic pathways that play an important role in defending the host from infection (37).

We next assessed whether SPE-based liposome administration would decrease cfDNA clearance in vivo. We administered liposomes to healthy mice, followed by exogenous mononucleosomes carrying the Widom601 sequence (W601) (38), then quantified the levels of W601 in plasma over time. We observed that the percentage of injected W601 in plasma 60 min after administration increased as liposome doses increased [mean increase between 9- and 3198-fold at liposome doses of 50 and 300 mg/kg, respectively, relative to phosphate-buffered saline (PBS) administration; *P* < 0.05] (Fig. 2D).

To determine whether attenuated clearance of mononucleosomes would translate to higher concentrations of endogenous cfDNA in plasma, we next administered liposomes or PBS into healthy mice and measured cfDNA levels in blood collected longitudinally. We observed an increase in the concentration of endogenous cfDNA in plasma after liposome injection (Fig. 2E), with peak concentration achieved 30 min after administration of a 100 mg/kg dose (10.3-fold increase over PBS) or 3 hours after administration of a 300 mg/kg dose (78.0-fold increase over PBS). Notably, levels returned to baseline within 5 and 24 hours of liposome treatment at the lower and higher doses, respectively, suggesting a transient effect of liposomes on cfDNA levels. We confirmed that SPE liposomes rapidly accumulated in MPS organs in vivo (Fig. 2E, insert, and fig. S6), where they can interact with liver macrophages responsible for mononucleosome clearance (fig. S7), and that depletion of MPS macrophages by means of liposomal clodronate eliminated the effect of liposomes on cfDNA clearance (fig. S8). Together, these results suggest that uptake of SPE nanoparticles by MPS macrophages can attenuate the cellular uptake of mononucleosomes and increase the recovery of endogenous cfDNA from a blood draw.

## Antibody priming agent protects cfDNA from clearance

We next investigated whether directly protecting cfDNA with a DNA-binding mAb could provide an alternative method to increase recovery of ctDNA in a blood draw. Of the eight known mouse anti-DNA immunoglobulin G (IgG) antibodies that we tested for double-stranded DNA (dsDNA) binding activity, four showed detectable binding at similar levels of affinity (fig. S9A). From these four, we selected a mouse IgG2a mAb (35I9) derived from a NZWxNZB F_1_ lupus-prone mouse for further investigation, given its reported biochemical and binding characterization, with dissociation constant (*K*_d_) of 90 and 700 nM to dsDNA and single-stranded DNA (ssDNA), respectively (39).

**Fig. 3.**
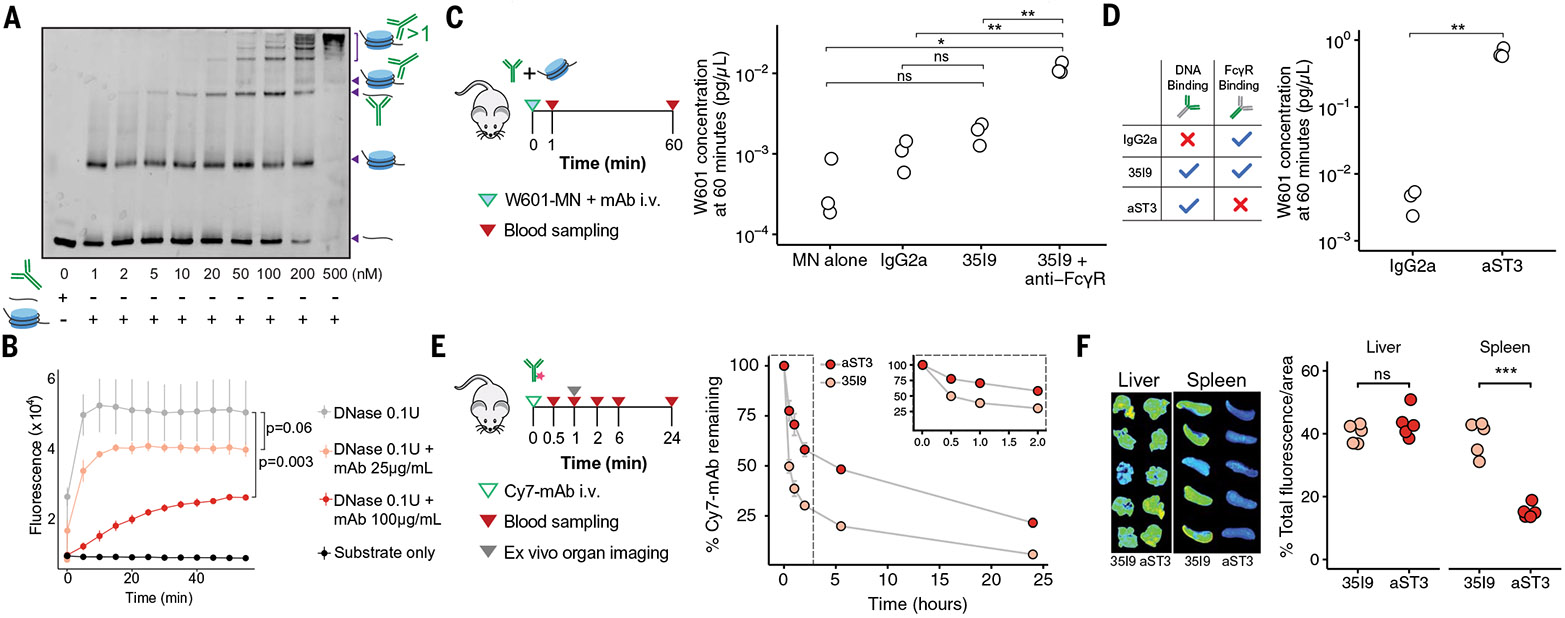
Antibody priming agent binds cfDNA and attenuates its clearance in healthy mice. (**A**) EMSA of free and histone-bound dsDNA (4 ng/μL per lane) with varying concentrations of DNA-binding mAb 35I9 in PBS (*N* = 3). (**B**) Fluorescence signal from the digestion of a DNA substrate carrying a hexachlorofluorescein dye on one end and a dark quencher on the other, with or without 0.1 U of DNase I and mAb 35I9. Points indicate mean and lines indicate SEM of three technical replicates. Fluorescence signals across the whole experiment were compared by using mixed models with replicates as random effects (*N* = 2). (**C**) (Left) Experimental approach to evaluate the effect of priming mAb on dsDNA clearance. (Right) Concentration of W601 in plasma 60 min after injection of W601 only, coinjection with an unrelated IgG2a antibody, with 40 μg of DNA-binding antibody 35I9, or with 40 μg of 35I9 together with anti-FcγRI (20 μg), anti-Fc(γ)RII, and anti-Fc(γ)RIII (40 μg) (*n* = 3 mice per group, *N* = 2). (**D**) (Left) Overview of the DNA binding and FcγR binding properties of engineered mAb aST3 versus IgG2a control mAb and DNA-binding mAb 35I9. (Right) Concentration of W601 in plasma 60 min after coinjection of W601 with an unrelated IgG2a antibody or with the Fc-mutant aST3 DNA-binding antibody (*n* = 3 mice per group, *N* = 3). (**E**) (Left) Experimental approach to quantify pharmacokinetics of 3519 and aST3 labeled with AQuora750 in plasma. (Right) Plasma clearance of antibodies over time (mean ±SEM, *n* = 5 mice per group, *N* = 1). (**F**) Biodistribution and quantification of 3519 (Fc-WT antibody) or aST3 concentration in liver and spleen 1 hour after administration (*n* = 5 mice per group, *N* = 1). [(C), (D), (F)] ns, not significant; *P < 0.05; **P < 0.01; ***P < 0.001; one-way ANOVA.

We first explored the interaction of 35I9 with elements of cfDNA (mononucleosomes and free dsDNA) using electrophoretic mobility shift assays (EMSAs). EMSA of 35I9 with a mixture of free and histone-bound 147-bp dsDNA revealed H3-negative bands corresponding to discrete ratios of mAb-to-dsDNA, as well as H3-positive bands corresponding to the binding of one or more than one mAb to histone-bound dsDNA (Fig. 3A and fig. S9B). 35I9 demonstrated rapid association and dissociation kinetics and similar binding affinity to various dsDNA oligonucleotides in vitro (fig. S10). To evaluate whether the observed interactions would interfere with nuclease activity, we next characterized the susceptibility of a fluorescence-quenched dsDNA probe to deoxyribonuclease (DNase) I degradation when incubated with different concentrations of 35I9. The fluorescent signal generated by cleavage of DNA in the presence of DNase I diminished with increasing concentrations of 35I9 (Fig. 3B). Together, these data demonstrate the ability of mAbs to interact with both free and histone-bound DNA and protect dsDNA from nuclease digestion.

To test mAb activity in vivo, we injected mononucleosomes carrying W601 with 35I9, without 35I9, or with IgG2a control into mice and measured the concentrations of W601 in plasma over time (Fig. 3C). Although the relative clearance of W601 was significantly (*P* < 0.05) delayed with mAb treatment (fig. S11), the absolute quantity of W601 recovered at 60 min was similar between 35I9 and the IgG2a control (Fig. 3C). We hypothesized that this lack of difference at 60 min was due to Fc-γ receptor (FcγR)–mediated clearance of dsDNA-35I9 complexes (40, 41). This effect could relate to some of the larger complexes observed in vitro (Fig. 3A), which would be expected to be sequestered and cleared rapidly through FcγR in vivo (42). Coinjection of the W601-mAb preparation and antibodies blocking mouse Fc(γ)RI, Fc(γ)RII, and Fc(γ)RIII yielded higher W601 levels at 60 min (0.012 pg/μL versus 0.00043 pg/μL; *P* = 0.007) (Fig. 3C). Together, these results suggest that administration of DNA-binding mAbs can delay the clearance of dsDNA from blood, but that FcγR-mediated clearance of dsDNA bound to mAb reduces the benefits for prolonged stabilization of the dsDNA.

Engineered variants of the Fc domain of mAbs provide one way to modulate interactions with FcγR and have been used in biopharmaceutical candidates clinically (43). We selected three sets of sequence variants known to disrupt FcγR binding (44)– aglycosylated N297A (denoted aST2) (45-47), L234A/L235A/P329G (denoted aST3) (48), and D265A (denoted aST5) (49, 50) (Fig. S12). All three variants still bound to dsDNA (Fig. S13). In vivo, aST3 yielded the highest recovery of W601 at 60 min (Fig. 3D, Fig. S14; 0.641 pg/μL vs. 0.004 pg/μL, P = 0.007), and was investigated further. We compared the pharmacokinetics of fluorophore-labeled aST3 and the Fc–wild type (WT) equivalent 35I9 mAb and observed that aST3 levels were elevated in plasma (Fig. 3E). W601 levels were below the limit of detection by 24 hours with or without aST3 (fig. S15), consistent with a transient effect. We also compared the biodistribution of aST3 and 35I9. The area-corrected accumulation of both mAbs was similar in the liver (fig. S7) but reduced in the spleen (Fig. 3F), suggesting differences in the clearance of aST3 by the two organs (51). Together, these data suggest that aST3, a DNA-binding mAb with abrogated FcγR binding, protects cfDNA from enzymatic digestion, has higher persistence in circulation, and increases cfDNA recovery from plasma compared with the native mAb and an IgG2a control.

## Nanoparticle priming agent improves tumor detection

**Fig. 4.**
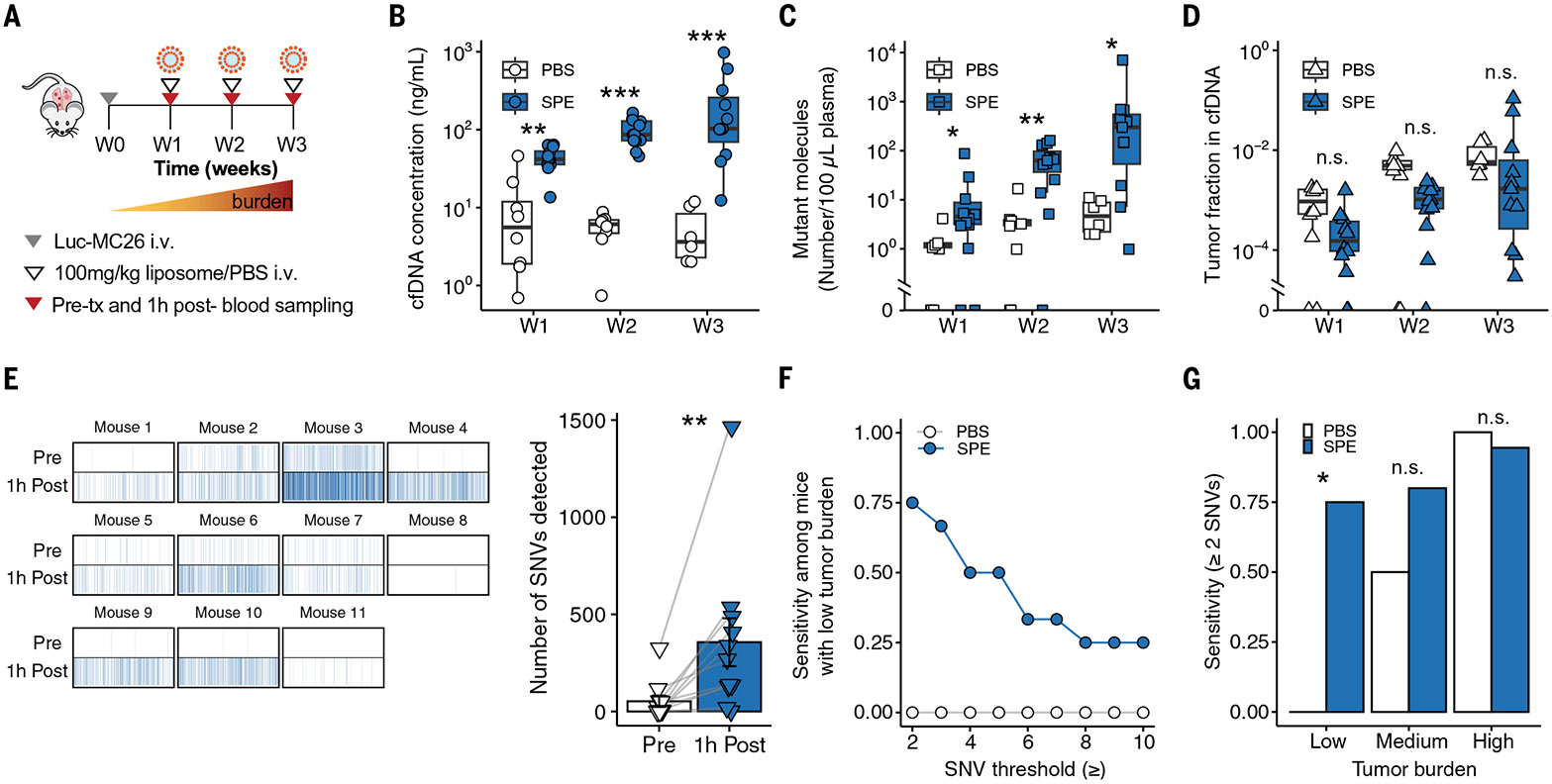
Liposome priming improves ctDNA recovery and enables detection of smaller tumors in a murine lung metastasis model. (**A**) Experimental approach for the detection of mutations from the plasma of Luc-MC26 tumor-bearing mice using the liposome priming agent. For each mouse, blood was drawn prior to and 1 hour after i.v. administration of PBS or SPE liposomes (100 mg/kg) at 1 week (W1), 2 weeks (W2), and 3 weeks (W3) after tumor inoculation. (**B**) Plasma cfDNA concentrations, (**C**) concentration of mutant molecules detected, and (**D**) tumor fractions 1 hour after PBS (white) or SPE (blue) administration at W1, W2, or W3 (*n* = 6 to 12 mice per group). (**E**) (Left) Mutational fingerprints showing distinct SNVs detected pre– and post–SPE administration for mice with high tumor burden (burden > 1.5e8 p/s total flux, as measured by IVIS). Each vertical band corresponds to a SNV in our 1822-SNV panel and is colored blue if detected at least once in the plasma sample. (Right) Quantification of these distinct SNVs. (**F**) Sensitivity of ctDNA tests versus SNV threshold for tumor detection in mice with low tumor burden after administration of PBS or SPE liposomes (burden < 1.5e7 p/s total flux, as measured by IVIS). (**G**) Sensitivity of ctDNA tests for different tumor burdens after PBS or SPE administration (Low, burden < 1.5e7 p/s; Medium, 1.5e7 p/s ≤ burden ≤ 1.5e7 p/s; High, burden > 1.5e8 p/s). Sensitivity was calculated as the fraction of samples for which the number of SNVs detected in a blood sample was ≥ 2 (*n* = 6 to 12 mice per group; *P < 0.05, Chi-squared test) (fig. S20, independent replicate at week 2). Boxplots in (B), (C), (D), and (E) show median and interquartile range. ns, not significant; *P < 0.05; **P < 0.01; ***P < 0.001; one-way ANOVA.

Because both liposomal and antibody priming agents showed increased recovery of cfDNA in healthy mice, we next explored whether they could enhance ctDNA-based tumor detection using a tumor-informed approach, tracking 1822 tumor-specific single-nucleotide variants (SNVs) (9, 52) (fig. S16). After selecting one hour as the optimal time post liposome administration for blood sampling (fig. 2E) (52), we performed an experiment with escalating doses of liposomes in a flank tumor model (figs. S17 and S18 and data S1) and selected a liposome dose of 100 mg/kg for further testing in a more disease-relevant transplantation model of lung metastases (fig. S19 and data S2). In this model, the luciferized MC26 cell line (Luc-MC26) was injected intravenously to establish lung metastases. Plasma was collected once a week at different stages of tumor progression, each time at one hour after administration of liposomes or PBS (Fig. 4A). We observed that administration of liposomes significantly (*P* < 0.05) increased concentrations of plasma cfDNA (7-fold, 14-fold, and 28-fold at weeks one, two, and three, respectively) (Fig. 4B) and the number of mutant molecules recovered at each time point (4-fold, 19-fold, and 60-fold) (Fig. 4C) relative to PBS-treated mice (data S3; independent replicate at week two, fig S20 and data S4). The maximum improvement in mutant molecule recovery (~60-fold) was observed at week three. Moreover, additional SNVs were detected after the administration of liposomes (6-fold and 90-fold higher at two and three weeks, respectively) (figs. S21 an S22). Liposome administration did not significantly decrease the tumor fractions (the fraction of total cfDNA originating from the tumor) in this experiment (*P* > 0.05) (Fig. 4D) but did reach significance (*P* < 0.05) in an independent cohort (fig. S20). Incubating primary murine white blood cells with liposomes in vitro led to a dose-dependent increase in the detection of DNA in conditioned medium, as measured using SYTOX green dye (fig. S23), suggesting that cfDNA release by white blood cells exposed to high concentrations of liposomes (53) may contribute to the modest decrease in tumor fraction observed.

We next assessed how the enhancement in recovered mutant molecules would impact the performance of ctDNA analyses, such as tumor genome profiling and sensitivity for tumor detection. In the absence of priming, most high-burden tumors (burden > total flux 1.5e8 p/s) were detectable, but priming with liposomes enabled detection of 67-fold (median) more SNVs than PBS (Fig. 4E), providing a more comprehensive molecular profile of these tumors. We next evaluated the sensitivity of ctDNA testing with and without priming by classifying each plasma sample as ctDNA positive only if the number of SNVs detected surpassed a given SNV threshold (between 2 and 10 SNVs, from lower to higher test stringencies). The liposomes improved the sensitivity of the ctDNA test (defined as the fraction of samples that were classified as ctDNA positive), regardless of SNV threshold, with the largest improvement in sensitivity observed in the group with the lowest tumor burden (burden < total flux 1.5e7 p/s) (Fig. 4F and fig. S24). By using a threshold of two SNVs, as has been previously applied to clinical samples (9), cancer was not detected in any of the untreated low–tumor burden mice, whereas 75% of liposome-primed mice were diagnosed as tumor-bearing with the same threshold. Improvements in sensitivity became smaller in the medium- and high-burden groups, as the untreated cohorts already had substantial levels of ctDNA prior to priming. Furthermore, the liposomes did not affect tumor progression (fig. S25) or evoke acute toxicity or weight loss after repeated dosing in healthy mice (fig. S26). Taken together, these results suggest that the nanoparticles enable profiling of more of the tumor genome and improve the sensitivity of a ctDNA-based test to enable detection of smaller tumors in preclinical models.

## Antibody priming agent improves tumor detection

**Fig. 5.**
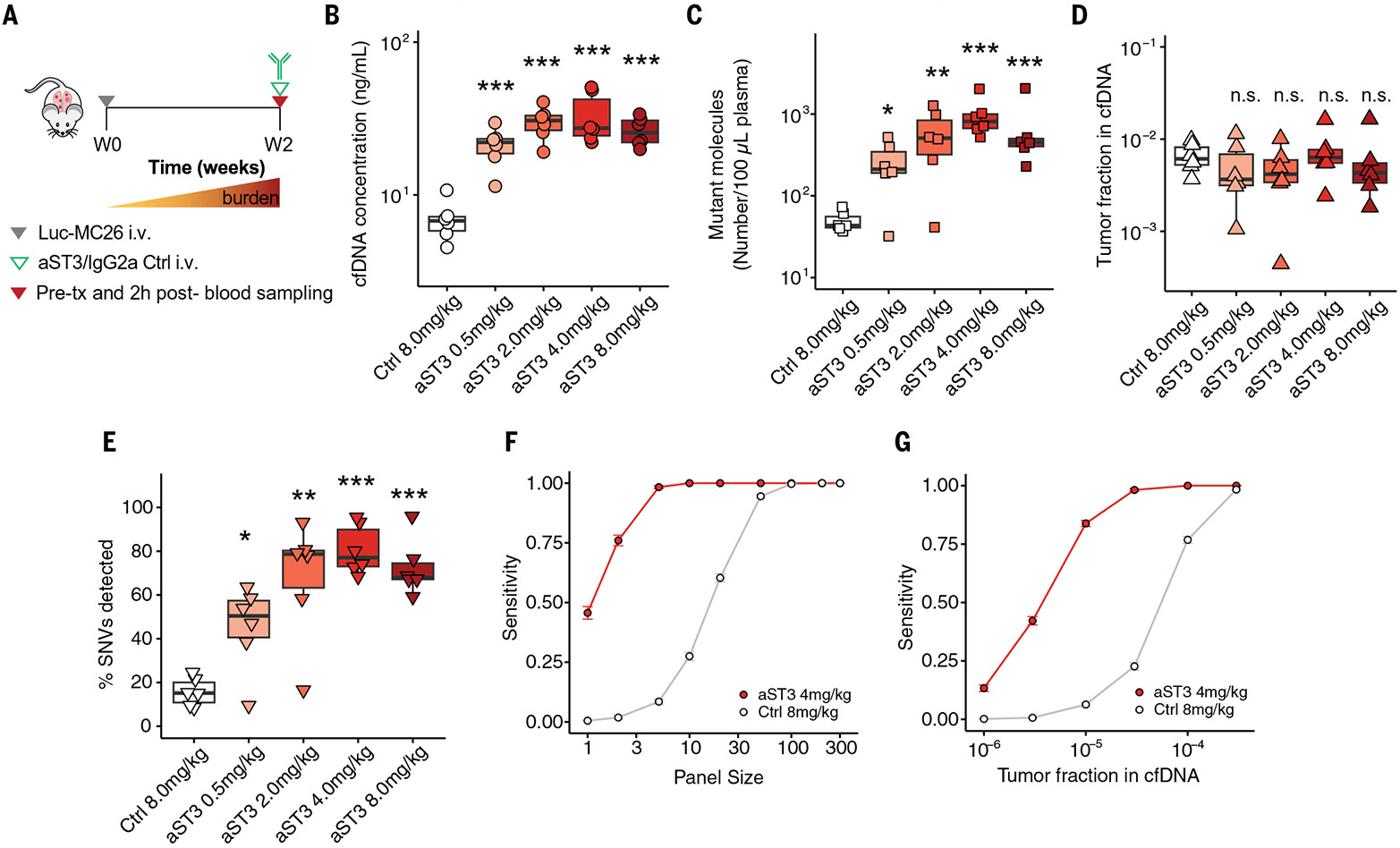
Antibody priming agent improves ctDNA recovery in murine lung metastasis model. (**A**) Experimental approach for the detection of mutations from the plasma of Luc-MC26 tumor-bearing mice with the antibody priming agent aST3. (**B**) Plasma cfDNA concentrations, (**C**) concentration of mutant molecules detected, and (**D**) tumor fractions detected 2 hours after administration of IgG2a control mAb or various doses of aST3 (*n* = 6 mice per group) (fig. S29, independent replicate at aST3 4.0 mg/kg). (**E**) Percentage of distinct SNVs from an 1822-SNV panel detected in plasma with control mAb or various doses of aST3. (**F** and **G**) Estimation of sensitivity for detection of ctDNA upon administration of 8 mg/kg of IgG2a control or 4 mg/kg of aST3 versus (F) panel size (G) or tumor fraction based on binomial down-sampling of mutant molecules, with a detection threshold of ≥ 2 SNVs (mean ± SEM, *n* = 100 replicates). Boxplots in (B) to (E) show median and interquartile range. ns, not significant; *P < 0.05; **P < 0.01; ***P < 0.001; one-way ANOVA.

We next explored the effect of our antibody priming agent on ctDNA-based tumor detection in the same transplantation model of lung metastases. We tested our antibody priming agent at a range of doses (0.5 to 8 mg/kg aST3 versus IgG2a Control at 8 mg/kg) at a single time point (2 weeks) during tumor progression (Fig. 5A). We sampled blood two hours after administering the mAb, as this interval corresponded to the peak accumulation of endogenous cfDNA in plasma after injection of aST3 in healthy mice (fig. S27). Accordingly, we also observed significantly (*P* < 0.001) higher recovery of cfDNA from plasma at all concentrations of mAbs (compared with an IgG2a isotype control) in tumor-bearing mice (Fig. 5B).

Administration of mAb resulted in consistently higher concentrations of mutant molecules with aST3 compared with IgG2a control, with a dose-dependent improvement between 0.5 and 4.0 mg/kg (Fig. 5C, fig. S28, and data S5; independent replicate at 4.0 mg/kg aST3, fig. S29 and data S4). The maximum effect occurred at a dose of 4.0 mg/kg, with a median 19-fold improvement over IgG2a isotype control. With this agent, no difference in tumor fraction was observed between the groups post injection (Fig. 5D). We also detected more total SNVs when priming with the engineered mAb (median 77% of SNVs detected with 4.0 mg/kg versus 15% detected with IgG2a isotype control) (Fig. 5E), again suggesting that priming improves the genomic profiling of tumors from a liquid biopsy. Consistent with the nuclease protection afforded by the DNA-binding mAb (Fig. 3B), we also found that priming resulted in greater enrichment of parts of the genome close to or overlapping with DNase hypersensitivity peaks (figs. S30, A to C, and S31). We also observed enrichment of sites with higher GC content and those overlapping CpG islands (figs. S30, D and E, and S31) (52).

We next investigated the effect of our priming agent on the sensitivity of ctDNA assays. Recognizing that our conditions in this preclinical model may not be representative of current commercial assays that typically have smaller mutation panels (9, 54, 55), or of much lower tumor fractions typically observed in early detection and minimal residual disease settings, we estimated the benefit of priming in such settings through a computational down-sampling approach (52). Across a wide range of panel sizes and detection thresholds, we consistently observed superior sensitivity with our priming agent compared with that of the IgG2a isotype control (Fig. 5F and fig. S32). We then evaluated the effect of our priming agent on ctDNA assay sensitivity at lower tumor DNA abundance (lower tumor fractions) (fig. S33) (52) and found that our priming agent resulted in similar sensitivity to that of the IgG2a isotype control at approximately 10-fold lower tumor fraction, irrespective of the SNV threshold used (fig. S34). By using a threshold of two SNVs, priming with aST3 improved the sensitivity across all different tumor fractions modeled, including at tumor fractions of 1 to 10 parts per million that are typical in the context of low tumor burden or minimal residual disease (Fig. 5G) (11, 56). These detection levels were reached in samples of mouse plasma with mean volumes of only 0.33 mL (SD, 0.09 mL), >10-fold less than plasma from a typical blood draw in humans (4 mL).

## Discussion

We have developed intravenous priming agents for liquid biopsies: agents that are given 1 to 2 hours prior to a blood draw to enable recovery of more cfDNA in a blood sample. The liposomal nanoparticles attenuate the uptake capacity of cfDNA by liver macrophages, whereas the DNA-binding antibody aST3 protects the cfDNA itself from nuclease degradation and plasma clearance. Both agents increase the recovery of ctDNA molecules from blood >10-fold, enable more of the tumor genome to be recovered in a blood draw, and enhance the sensitivity of ctDNA diagnostic tests.

Our priming agents intervene in vivo on the natural clearance pathways of cfDNA to boost ctDNA recovery, addressing the well-recognized barrier of low quantities of input cfDNA that limits the sensitivity of liquid biopsy tests (16, 57, 58). Sampling larger blood volumes has traditionally been used to increase the total quantity of cfDNA available for assays, but with only modest linear increases in recovery given the notable practical limitations on sampling large volumes of blood. The priming agents we describe increase the concentration of cfDNA in blood prior to sampling. These approaches are also distinct from those that rely on local sampling, such as lymph fluid or bronchoalveolar lavage (22, 59), because they preserve the advantages of a blood draw: sampling from all potential disease sites and avoiding the need for specialized, invasive, and disease-specific sampling procedures. Our antibody priming agent showed 86% sensitivity at 1∕100,000 tumor fraction in 0.33-mL mouse plasma samples, a sensitivity on par with the best-performing genome-wide cfDNA tests reported to date, which use >10-fold higher plasma volumes from patient plasma samples (57, 60). When scaling the sample volumes from mouse plasma to typical clinical blood draws, the sensitivity afforded by our priming agents could far exceed those reported in the literature. Furthermore, because these priming agents are given prior to collecting and processing liquid biopsies, they could also enhance existing genome-wide workflows (16, 18, 19) to maximize sensitivity.

Although results from our proof-of-concept studies in preclinical models are encouraging, it remains to be determined how these strategies would translate clinically. Further development prior to clinical testing of either agent would involve preclinical optimization, formulation, testing, and tolerability in other animal models. For the nanoparticles, optimizing formulations by using emerging technologies in nanoparticle engineering (61, 62) could improve potency and mitigate dose-dependent reductions in tumor fractions. Additionally, investigating the cellular mechanisms driving the inhibition of cfDNA uptake, which may involve changes in membrane availability or composition (i.e., competition for or internalization of receptors) or feedback mechanisms in phagocytic signaling networks (30, 63), could reveal additional avenues for development. For the antibody, higher affinity or alternative cfDNA binders could be explored to further improve recovery of cfDNA. One clinically relevant observation to support the translational potential of the antibody is from studies of the autoimmune disease systemic lupus erythematosus. A feature of this disease is elevated levels of anti-DNA antibodies. Higher concentrations of cfDNA have been associated with increased titers of anti-DNA antibodies along with reduced degradation of extracellular DNA (64, 65). These observations support the potential efficacy of an antibody priming agent in humans. Furthermore, engineering of the Fc-effector function, as we demonstrated, could reduce or eliminate potential safety risks related to Fc-mediated immune activation (66-68) for transient administration of low doses, as tested here (49, 69). In our testing, no sign of acute toxicity was observed with either agent. Future development work will be needed to evaluate safety in other animal models prior to first-in-human testing.

Because the two approaches have different targets (liver macrophages for nanoparticles and cfDNA in blood for antibodies), each has distinct advantages as a priming agent. For nanoparticles, interfering with the uptake capacity of macrophages could potentially enhance the recovery of other circulating analytes cleared through similar pathways. For antibodies, their target specificity could be further engineered to enhance the recovery of other analytes or of subpopulations of cfDNA molecules, such as those carrying specific epigenetic marks. The optimal approach would depend on the intended application. With our two approaches targeting different processes, a broad range of potential diagnostic applications as well as possible combinations of the two could be considered.

We envision that the initial clinical use of our priming agents could be in patients with a previous cancer diagnosis in which tumor detection or monitoring sensitivity is currently lacking. Priming could boost the sensitivity of minimal residual disease tests to guide clinical decisions, such as the use of adjuvant therapy or evaluating the efficacy of nonsurgical organ-preserving treatments. In patients with advanced cancer, priming could enable the detection of rare targetable mutations missed by conventional liquid biopsies. Looking ahead, priming could also boost the sensitivity of liquid biopsy cancer screening tests and would be especially useful for individuals at elevated risk of cancer, with nonspecific symptoms that may be associated with cancer, or with indeterminate findings from other diagnostics such as imaging scans. A notable example would be indeterminate nodules on lung computed tomography scans. Furthermore, given that our priming agents modulate cfDNA clearance, their use could be considered in applications beyond oncology. Priming could improve detection of microbial cfDNA during early or deep-seated infections (70), where diagnosis is critical for therapy selection but remains challenging. Liquid biopsy–based applications in cardiovascular disease and Alzheimer’s disease are other areas where the low abundance of cfDNA is a limitation, and where priming agents may be beneficial (71, 72). Deeper characterization of the effect of priming on other aspects of cfDNA, such as epigenetics and fragmentomics, could reveal further insights into cfDNA biology and motivate other applications. We believe that the concept of a priming agent capable of perturbing endogenous biomarker clearance in vivo can change how we think about the limit of diagnostic detection. These approaches should spark interest in the field, not only for further development of related priming agents for cfDNA detection, but also for improved detection of other circulating biomarkers.

In this work, we present liquid biopsy priming agents that improve the sensitivity and the robustness of ctDNA testing in tumor-bearing mice by modulating cfDNA clearance. Just as iodinated and gadolinium contrast agents greatly improve the sensitivity of clinical imaging, we envision that priming agents can boost the sensitivity of liquid biopsies in cancer care and for indications beyond oncology.

## Materials and methods summary

### Liposome synthesis and characterization

Liposomes were prepared using the lipid film rehydration method with slight modifications from the protocol described by Saunders *et al.* (31). Briefly, ovine cholesterol (50 mol %, cat. 700000P, Avanti Polar Lipids) was solubilized in chloroform and added to 1,2-dipalmitoylsn-glycero-3-phosphoethanolamine-N-(succinyl) (sodium salt) (SPE) (50 mol %, cat. 870225P, Avanti Polar Lipids), 1,2-distearoyl-sn-glycero-3-phospho-(1'-rac-glycerol) (sodium salt) (DSPG) (50 mol %, cat. 840465P, Avanti Polar Lipids), or 1,2-distearoyl-sn-glycero-3-phosphocholine (DSPC) (50 mol %, cat. 850365P, Avanti Polar Lipids) with 1:1 (v/v) methanol. The solution was evaporated under nitrogen flow to form a thin dry film and vacuumed overnight to remove any traces of organic solvent. The lipid film was hydrated at 60°C with sterile Dulbecco’s phosphate-buffered saline (DPBS) to a total lipid concentration of 50 mg/ml. Extrusion was performed at 60°C with 1-μm (cat. WHA110410, MilliporeSigma) and 0.4-μm polycarbonate membranes (cat. WHA10417101, MilliporeSigma), 21 and 20 times respectively, using the 1000-μL Mini-Extruder from Avanti Polar Lipids (cat: 610023). For the fluorescent liposome used for biodistribution studies, 0.2 mol % of SPE was replaced for Cy7-SPE (cat: 810347C, Avanti Polar Lipids) prior to solubilization with organic solvents. The hydrodynamic diameter and polydispersity index of liposomes was characterized using a Zetasizer NanoZS (Malvern Instruments). The morphology of liposomes was confirmed by cryo–transmission electron microscopy imaging.

### Mononucleosome preparation and labeling

To prepare mononucleosomes, chromatin was extracted from CT26 cells following manufacturer’s recommendations using the Nucleosome Preparation Kit (cat. 53504, Active Motif). The enzymatic digestion time was optimized as 30 min, and the resulting mononucleosomes were confirmed via electrophoresis through a 1.5% agarose gel. Subsequently, aliquots of 10 μg mononucleosomes were washed and buffer-exchanged into PBS. Four washes were performed using 30-kDa Amicon filters (cat. UFC503024, EMD Millipore) by centrifugation at 12,000 rpm for 10 min at 4°C. The protein yield was calculated using a commercial HeLa mononucleosome standard by measuring absorbance at 230 nm using a Nanodrop 8000 Spectrophotometer (cat. ND-8000-GL, Thermo Fisher). To label mononucleosomes, sulfonated-Cy5 (cat. 13320, Lumiprobe) was added at a 25:1 molar ratio of dye to protein, and the reaction incubated at 4°C in an Eppendorf Thermomixer C Model 5382 (Eppendorf) at 550 rpm overnight. Excess dye was removed using Micro Bio-Spin Columns with Bio-Gel P-6 (cat.7326221, BioRad) by centrifugation at 1000*g* for 2 min at room temperature. Labeling efficiency was quantified by measuring Cy5 intensity at 650/680 nm against a Cy5 standard using an Infinite F200 Pro reader (Tecan) fluorometer and protein yield was estimated by measuring absorbance at 230 nm using a Nanodrop 8000 Spectrophotometer.

### In vitro macrophage mononucleosome uptake inhibition assay with liposomes

J774A.1 (TIB-67, ATCC) cells were plated at a density of 30,000 and 45,000 cells per chamber, respectively, in 8-well chamber slides (cat.80806, Ibidi). Following overnight acclimatization, cells were incubated with 300 μL of liposomes (SPE, DSPG, or DSPC) diluted in Dulbecco's Modified Eagle Medium (DMEM) at the desired concentrations (0.1 to 5 mg/ml) for 4 hours at 37°C. Next, 30 μL of mononucleosomes were spiked into each well to achieve a final mononucleosome concentration of 10 nM and further incubated for 2 hours at 37°C. Cells incubated with DMEM followed by mononucleosome addition were used as a positive control for uptake, and cells incubated only with DMEM were used as a negative control. At the end of the incubation, cells were washed once with DMEM, stained with Hoechst 33342 (cat. H3570, ThermoFisher) at 1:2000 dilution in DMEM for 10 min at room temperature, and further washed (twice with DMEM and once with PBS) to remove any extracellular mononucleosomes. Subsequently, cells were fixed with 4% PFA for 20 min at room temperature and washed with PBS prior to imaging on an Eclipse Ti microscope (Nikon).

To quantify cellular uptake, four fields of view per well were obtained at 10X magnification, and mean Cy5 fluorescence intensity per cell was quantified using custom scripts in QuPath (73). Results are displayed after background subtraction using the mean Cy5 fluorescence intensity per cell from the negative control.

### Electrophoretic mobility shift assays (EMSA)

Widom601 dsDNA complexed with recombinant human histones was purchased from Epicypher (cat. 16-0009). dsDNA (free and/or histone bound) was combined at a final concentration of 4 ng/μL total DNA with varying concentrations of 35I9 (Abcam ab27156) in PBS (21-040-CM, Corning) in 10 μL total volume. 1 μL of Novex high density TBE sample buffer (cat. LC6678, Thermo Fisher Scientific) was added, and 10 μL of mixture was loaded into 6% DNA Retardation Gels (cat. EC6365BOX, Thermo Fisher Scientific). Gels were run at 4°C, 100 V for 120 min in 0.5x TBE, stained with SYBR Safe (cat. S3312, Thermo Fisher Scientific) at 1:10000 dilution in 0.5x TBE for 30 min, and imaged on an ImageQuant LAS4000.

### DNase protection assays

To measure sensitivity to DNase digestion, the DNaseAlert kit (cat. 11-02-01-04, IDT) was used in combination with various concentrations of recombinant DNase I and antibody 35I9 in 100-μL reactions incubated at 37°C in a Tecan microplate-reader with initial measurement before addition of DNase I and subsequent measurements every 5 min after addition of DNase I (excitation 365 nm, emission 556 nm).

### Animal models

All animal studies were approved by the Massachusetts Institute of Technology Committee on Animal Care (MIT Protocols 042002323, 2301000462). Female BALB/c mice (6 to 10 weeks, Taconic Biosciences) were used for all healthy mice experiments. To generate the CT26 flank tumor model, female BALB/c mice (6 weeks, Taconic Biosciences) were injected subcutaneously with 2 × 10^6^ CT26 cells resuspended in Opti-Mem (cat. 11058021, Thermo Fisher) into bilateral rear flanks. Tumors were measured every other day for 2 weeks, and tumor volumes were calculated by the modified ellipsoidal formula *V* = 0.5 × (*l* × *w*^2^), where *l* and *w* are the tumor length and width, respectively. To generate the transplantation model of lung metastasis, 1 × 10^5^ Luc-MC26 cells in 100 μL of DPBS were injected intravenously (i.v.) into female BALB/c mice (6 weeks, Taconic Biosciences). Tumor growth was monitored by luminescence using the In Vivo Imaging System (IVIS, PerkinElmer) on days 6, 13, and 20 after tumor inoculation.

### Blood collection

Retroorbital blood draws (70 μL in general, 35 μL for antibody pharmacokinetic study) were collected by means of nonheparinized capillary tubes from mice under isoflurane anesthesia, alternating between eyes for serial draws. Blood was immediately displaced from the capillary tube into 70 μL of 10-mM EDTA (cat. AM9260G, Thermo Fisher Scientific) in PBS. For terminal bleed samples, blood was collected through cardiac puncture into a syringe filled with 200 μL of 10-mM EDTA in PBS. Total volume was measured and an additional 10 mM of EDTA in PBS was added to reach a 1:1 ratio of blood to EDTA. Blood with EDTA was kept on ice and centrifuged within 90 min at 8000*g* for 5 min at 4°C. The plasma fraction was collected and stored at −80°C until further processing.

### cfDNA extraction and quantification

Frozen plasma was thawed and centrifuged at 15,000*g* for 10 min to remove residual cells and debris. PBS was then added into plasma to make the total volume 2.1 ml for cfDNA extraction using the QIAsymphony Circulating DNA kit (cat:937556, Qiagen). The extracted cfDNA was quantified using a Taqman quantitative polymerase chain reaction (qPCR) assay targeting a locus in the mouse genome and then kept at 4°C until ready for further processing.

### In vivo mononucleosome pharmacokinetic studies

SPE liposomes or sterile DPBS were administered i.v. into awake mice (50 to 300 mg/kg, 200 μl). 30 min after liposome injection, 1 μg of recombinant mononucleosomes carrying the Widom601 (W601) sequence (cat. 81070, ActiveMotif) suspended in 10 μL of DPBS were injected i.v. into anesthetized mice. In the study evaluating the percentage of exoNCP remaining at 60 min (*n* = 4 per group), 70 μL of blood was drawn retro-orbitally 1 and 60 min after mononucleosome injection. For the mAb pharmacokinetic assay, 10 to 20 ng of W601 sequence (cat. 81070, ActiveMotif) was combined with antibody in 200 μL of PBS. Engineered variants were produced in house; 35I9 was purchased from Abcam (cat. ab27156); mouse IgG2a control (clone 20102, cat. MAB003), anti-FcγRII/III (rat anti-mouse, clone 190909, cat. MAB1460), and anti-FcγRI (rat anti-mouse, clone 29035, cat. MAB2074) were purchased from R&D systems. 40 μg of anti-FcγRII/III and 20 μg of anti-FcγRI were used in FcγR-blocking conditions. Each mouse was anesthetized with inhaled isoflurane and injected i.v. with 200 μL of mixture. At 1 min after injection, 70 μL of blood was collected through a retro-orbital blood draw. Mice were allowed to recover after this and between subsequent blood draws (all 70 μL). Percentage of W601 remaining was calculated as the percentage of W601 remaining at 60 min relative to 1 min, as quantified using Taqman qPCR.

### Plasma cfDNA concentration measurements following liposome administration

100 or 300 mg/kg SPE liposomes (200 μL in sterile DPBS) or DPBS were administered i.v. in awake mice (*n* = 3 mice per group). At 1 and 30 min and 1, 3, 5, and 24 hours after liposome administration, 70 μL of blood was collected retro-orbitally. Only two blood samples were collected from each mouse to prevent repeated sampling from the same capillary bed. Plasma cfDNA concentration was quantified as described above. Given that cfDNA recovery was highest 30 min and 3 hours after liposome administration for the 100 mg/kg and 300 mg/kg doses, respectively, we decided to sample blood 1 hour after liposome administration, which allowed us to compare results from animals treated with different liposome doses in our tumor models.

### Antibody expression and purification

Desired Fc changes were introduced into the heavy-chain sequence (as determined by liquid chromatography–mass spectrometry de novo sequencing) and codon-optimized for expression in HEK293 cells. Gene blocks for the heavy and light chains were cloned into the same gWiz plasmid, separated by the T2A ribosome skipping sequence (74, 75). Expi293F cells at a density of 3 × 10^6^ cells/mL were transfected with 1 mg/L of culture of plasmid complexed with PEI Max 40K (cat. 24765-100, Polysciences) in a 1:2 plasmid:PEI w/w ratio in 40 mL of Opti-MEM (cat. 31985062, Thermo Fisher Scientific) per 1L culture. Flasks were kept in a shaking incubator (125 rpm) at 37°C and 8% CO_2_. 24 hours after transfection, flasks were supplemented with glucose and valproic acid (cat. P4543, Millipore Sigma) to final concentrations of 0.4% v/v and 3 mM, respectively. Culture supernatant was harvested after 5 to 6 days and purified using Protein A affinity chromatography (AKTA, Cytiva), buffer exchanged into PBS, and sterile filtered and stored at −80°C.

### Cell-line and buffy coat sequencing and fingerprint design

Genomic DNA (gDNA) was extracted from CT26 cells, Luc-MC26 cells, and Balb/c buffy coat, then sheared to 150 bp. gDNA libraries were prepared using the Kapa HyperPrep Library Construction kit (cat. KK8504, Roche Diagnostics). Whole-genome sequencing was performed to 30× coverage for CT26 and Luc-MC26, and 15× coverage for Balb/c buffy coat. Tumor fingerprints consisting of 98 and 1822 single-nucleotide variants (SNVs) were designed for CT26 and MC26 (data S1 and S2, respectively), as previously described (9).

### Library construction, hybrid capture, and sequencing

cfDNA libraries were constructed using the Kapa Hyper Prep kit (cat: 07962363001, Roche) with custom dual index duplex UMI adapters (IDT), as previously described (9). Hybrid capture (HC) using tumor specific panels was performed using the xGen hybridization and wash kit (cat: 1080584, IDT) with xGen Universal blockers (cat: 1075476; IDT). For the ctDNA diagnostic test, libraries were pooled up to maximum 12-plex, with a library mass equivalent to 25× DNA mass into library construction for each sample, and a panel consisting of 120-bp long probes (IDT) targeting tumor-specific SNVs was applied. After the first round of HC, libraries were amplified by 16 cycles of PCR and then carried through a second HC. After the second round of HC, libraries were amplified through 8 to 16 cycles of PCR, quantified, and then pooled for sequencing (151 bp paired-end runs) with a targeted raw depth of 40,000×per site per 20 ng of DNA input. Sequencing data were processed by our duplex consensus calling pipeline as previously described, yielding measurements of the total number of mutant duplexes detected, the unique number of loci detected, and the tumor fractions (9). Relative duplex depth at each site was computed by subtracting mean overall depth for the library and then dividing by the standard deviation to obtain a relative duplex depth.

### Assessing the performance of liposomal priming agent for tumor detection

Six days after tumor inoculation, mice bearing Luc-MC26 metastatic tumors were randomized into different treatment groups [100 mg/kg SPE liposomes (*n* = 12 mice) or PBS (*n* = 8 mice)] such that total burden was equivalent across different treatment groups (1.08 ± 0.5e7 photons/s for 100 mg/kg SPE liposomes versus 9.95 ± 5.2e6 photons/s for PBS). To determine how our liposomal priming affected ctDNA performance at different tumor burdens, priming was performed 1, 2, and 3 weeks after tumor inoculation. At each timepoint, 70 μL of blood was sampled retro-orbitally from each mouse prior to treatment as an internal control. Subsequently, 100 mg/kg SPE liposomes (in 200 μL sterile DPBS) or sterile DPBS were administered i.v. into awake mice. 1 hour after treatment, 70 μL of blood was collected retro-orbitally from the contralateral eye, and a terminal bleed was then performed. cfDNA concentration measurement and ctDNA detection was performed on all samples as described above.

To calculate the sensitivity of the ctDNA test for tumor detection, mice were grouped as a function of tumor burden into those with small (total burden < 1.5e7 photons/s), medium (1.5e7 photons/s < total burden < 1.5e8 photons/s), and large (total burden > 1.5e8 photons/s) tumors. Retro-orbital plasma samples were classified as ctDNA positive if the number of distinct SNVs detected surpassed a given SNV threshold (between 2 and 10 SNVs, from lower to higher stringency of the test), and sensitivity was calculated as the % of samples that were ctDNA positive per group.

### Assessing the performance of antibody priming agent for tumor detection

Between days 10 and 12 post–tumor inoculation, the performance of aST3 on ctDNA testing was assessed in Luc-MC26 tumor-bearing mice. As an internal control, 70 μL of blood was sampled retro-orbitally from each mouse prior to treatment. Subsequently, 4.0 mg/kg of aST3 (in 200 μL sterile DPBS) or 4.0 mg/kg of IgG2a isotype were administered into awake mice i.v. 2 hours after treatment, 70 μL of blood was collected retro-orbitally from the contralateral eye, and the remainder of the blood was collected by means of cardiac puncture. The 2 hour time point was chosen as it resulted in the highest endogenous cfDNA concentration in healthy mice after injection of aST3 (fig. S27). cfDNA concentration measurement and ctDNA detection was performed on all samples as described above.

### ctDNA sensitivity estimation

To estimate sensitivity at smaller panel sizes, we used a bootstrap procedure down-sampling with replacement from our 1822-site panel to smaller panel sizes. Sensitivity at different detection thresholds was estimated as the fraction of mice that had mutant molecules detected at the given threshold. For each panel size and dose, 100 replicates were generated, and the mean sensitivity and standard error was computed. To estimate sensitivity at lower tumor fractions, we first confirmed that the distribution of mutant molecules (*n_ij_*) and the distribution of the ratio of mutant molecules to total molecules (*n_ij_/t_ij_*) could be accurately recapitulated through a binomial sample *n_ij_* ~ Binom (*t_Ij_*, *f_i_*), where *n_ij_* is the number of mutant molecules at site *j* in sample *i*, *t_ij_* is the number of total molecules at site *j* in sample *i*, and *f_i_* is the global tumor fraction in sample *i*. To estimate sensitivity at lower tumor fractions, we then generated distributions of mutant molecules under lower *f_i_* for each sample, also incorporating various panel sizes as above, and computed sensitivity for detection of mutant molecules under various detection thresholds. Sensitivity at each *f_i_*, dose, and panel size was estimated by taking the mean and standard error from 100 replicates.

### Statistical analysis

One-way analysis of variance (ANOVA) was used for statistical testing unless noted otherwise. A suite of scripts (Miredas) was used for calling mutations and creating metrics files (9, 15). All other analysis was performed using GraphPad Prism v9, custom Python scripts, and R (v4.0.3) [code available on Zenodo (76)]. Detailed statistical information is provided in figure captions. For each animal experiment, mice were randomized such that groups would have comparable tumor burden. Investigators were not blinded to the groups or the treatments during the experiments.

Full materials and methods are available in the supplementary materials (52).

## Supplementary Material

Supplementary Materials

Data S1-S5

MDAR

## Figures and Tables

**Fig. 1.**
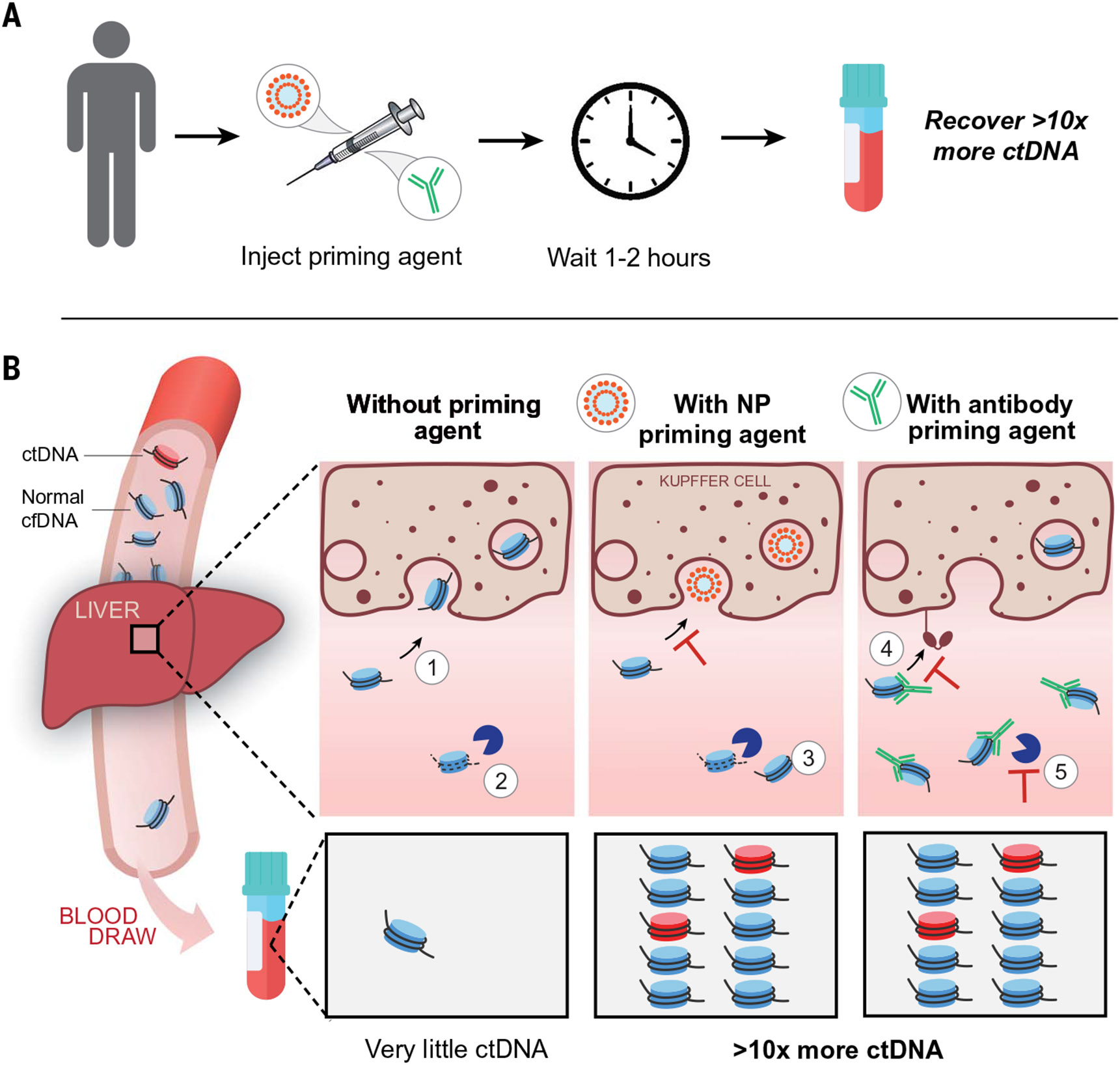
Priming agents reduce clearance of cfDNA and improve the recovery of ctDNA. (**A**) Priming agents are injected 1 to 2 hours prior to a blood draw and improve the recovery of ctDNA by >10-fold. (**B**) (Left) In the absence of a priming agent, cfDNA (mostly in the form of mononucleosomes) is (i) rapidly taken up by macrophages of the MPS in the liver and (ii) degraded by circulating nucleases, yielding little ctDNA molecules in a blood draw. (Center) Following intravenous administration of a nanoparticle (NP) priming agent, (iii) cellular uptake is attenuated through MPS saturation. (Right) Intravenous administration of an antibody priming agent (iv) extends the half-life of cfDNA in circulation and (v) protects it from nuclease digestion. Both priming strategies enhance ctDNA recovery and improve mutation detection from a blood draw.
