# Supplementary material for: Priming agents transiently reduce the clearance of cell-free DNA to improve liquid biopsies
Source: Science. Author manuscript; Available in PMC 2024 Nov 1. (PMC11529396; doi:10.1126/science.adf2341)
Supplement: Supplementary Materials [file NIHMS2028973-supplement-Supplementary_Materials.pdf]

Science AAAS

Supplementary Materials for

Priming agents transiently reduce the clearance of cell-free DNA to improve liquid biopsies

Carmen Martin-Alonso *et al*.

Corresponding authors: Shervin Tabrizi, shervin@broadinstitute.org; J. Christopher Love, clove@mit.edu; Sangeeta N. Bhatia, sbhatia@mit.edu; Viktor A. Adalsteinsson, viktor@broadinstitute.org

Science **383**, eadf2341 (2024)
DOI: 10.1126/science.adf2341

The PDF file includes:

Materials and Methods
Figs. S1 to S34
References

Other Supplementary Material for this manuscript includes the following:

MDAR Reproducibility Checklist
Data S1 to S5

Materials and Methods

Liposome synthesis

Liposomes were prepared using the lipid film re-hydration method with slight modifications from the protocol described by Saunders et al. (31) Briefly, ovine cholesterol (50 mol %, cat. 700000P, Avanti Polar Lipids) was solubilized in chloroform and added to 1,2-dipalmitoyl-sn-glycero-3-phosphoethanolamine-N-(succinyl) (sodium salt) (SPE) (50 mol %, cat. 870225P, Avanti Polar Lipids), or 1,2-distearoyl-sn-glycero-3-phospho-(1'-rac-glycerol) (sodium salt) (DSPG) (50 mol %, cat. 840465P, Avanti Polar Lipids) or 1,2-distearoyl-sn-glycero-3-phosphocholine (DSPC) (50 mol %, cat. 850365P, Avanti Polar Lipids) with 1:1 (v/v) methanol. The solution was evaporated under nitrogen flow to form a thin dry film and vacuumed overnight to remove any traces of organic solvent. The lipid film was hydrated at 60°C with sterile DPBS to a total lipid concentration of 50 mg/ml. Finally, extrusion was performed at 60°C with 1 µm (cat. WHA110410, MilliporeSigma) and 0.4 µm polycarbonate membranes (cat. WHA10417101, MilliporeSigma), 21 and 20 times respectively, using the 1000 µL Mini-Extruder from Avanti Polar Lipids (cat: 610023). For the fluorescent liposome used for biodistribution studies, 0.2 mol % of SPE was replaced for Cy7-SPE (cat: 810347C, Avanti Polar Lipids) prior to solubilization with organic solvents.

Liposome characterization

Liposomes were characterized using a Zetasizer NanoZS (Malvern Instruments). To measure the hydrodynamic diameter and polydispersity index (PdI) of the liposomes, samples were diluted 1:100 in PBS and analyzed. To measure the zeta potential of the liposomes, 10 µL of sample was added to 40 µL of PBS and 850 µL of deionized water. The morphology of liposomes was confirmed by cryogenic TEM (cryo-TEM) imaging. Prior to sample preparation, lacey copper grids coated with a continuous carbon film (LC200-Cu, Electron Microscopy Sciences) were plasma treated using a Denton Sputter Coater at 4 mA for 5 s. The cryo-TEM samples were prepared on a Gatan Cryoplunge III at room temperature and 100% humidity. Liposome solution (3 µL, 5 mg/mL in PBS) was dropped on the plasma-treated grids and the excess of samples was removed by blotting for 4 sec before plunge freezing. The frozen grid was mounted on a Gatan 626 single-tilt cryo-transfer holder and imaged using a JEOL 2100F at 200 kV. All images were taken at 30 kx or 40 kx using the minimum dose exposure system and recorded on a Gatan UltraScan CCD camera.

Mononucleosome preparation:

To prepare mononucleosomes for labeling, chromatin was extracted from CT26 cells using the Nucleosome Preparation Kit (cat. 53504, Active Motif). The enzymatic digestion time for this cell line was optimized to 30 min and the mononucleosome preparation protocol was followed as per manufacturer's recommendations. To verify that chromatin had been successfully digested to mononucleosomes, the solution was subjected to DNA cleanup, and digestion efficiency was assessed via electrophoresis through a 1.5% agarose gel.

Mononucleosome labeling:

Aliquots of 10 µg mononucleosomes were first washed and buffer-exchanged into PBS to remove any impurities from the preparation reagents. Four washes were performed using 30 kDa Amicon filters (cat. UFC503024, EMD Millipore) by centrifugation at 12,000 rpm, 10 min, 4°C. After concentrating the volume of the last wash to 40 µl, the protein yield was calculated using a

commercial HeLa mononucleosome standard by measuring absorbance at 230 nm using a Nanodrop 8000 Spectrophotometer (cat. ND-8000-GL, Thermo Fisher). To label mononucleosomes, sulfonated-Cy5 (cat. 13320, Lumiprobe) was added at a 25 dye: 1 protein molar ratio, and the reaction incubated at 4°C in an Eppendorf Thermomixer C Model 5382 (Eppendorf) at 550 rpm overnight. Excess dye was removed using Micro Bio-Spin® Columns with Bio-Gel P-6 (cat.7326221, BioRad) by centrifugation at 1000 *g*, 2 min at room temperature. Labeling efficiency was measured by measuring Cy5 intensity at 650/680 nm against a Cy5 standard using an Infinite F200 Pro reader (Tecan) fluorometer and protein yield by measuring absorbance at 230 nm using a Nanodrop 8000 Spectrophotometer.

<u>Mononucleosome characterization:</u>
Mononucleosome stability post-labeling was confirmed by running a 4-12% Tris Glycine Novex gel (cat. XP04122BOX, Thermo Fisher) with unlabeled or Cy5-labeled mononucleosomes (native running buffer, 4°C, 150 V, 90 min). Colocalization of a Cy5-positive band with a protein band after Coomassie staining corresponding to the molecular weight of a mononucleosome was confirmed. Additionally, the morphology of mononucleosomes prepared from the chromatin of CT26 cells was compared to the morphology of mononucleosomes purchased commercially and used for in vivo pharmacokinetic studies (cat. 81070, ActiveMotif) using negative-stained TEM imaging. For sample preparation, first, ultrathin carbon film Au grids (CF300-Cu-UL, Electron Microscopy Sciences) were plasma treated using a Denton Sputter Coater at 5 mA for 8 s. Next, 5 µL of NCP solution (17 ng/µL in PBS) was incubated on the plasma-treated grids for 1.5 min and the excess of liquid was removed with a filter paper (pore size 110 nm, Whatman). The grid was washed twice by dropping 5 µL of deionized water on the grids and quickly removed using a filter paper. Negative staining was performed by dropping 5 µL of 2% uranyl acetate solution on the grid for 18–20 s and the excess of liquid was removed by a filter paper. Imaging was performed on a JEOL 2100F TEM at 200 kV. All images were recorded on a Gatan UltraScan charge-coupled device (CCD) camera.

<u>Cell culture</u>
For in vitro mononucleosome uptake experiments, mouse macrophage cell lines RAW264 (TIB-71, ATCC) and J774A.1 (TIB-67, ATCC) were cultured in DMEM. Both media were supplemented with 10% fetal bovine serum (cat:100-500, GemCell) and 1% penicillin/streptomycin (cat. 30-002-C1, Corning), and cells were cultured in a humidified atmosphere of 95% air and 5% CO_2 at 37°C. For tumor inoculations, mouse cell lines CT26 (cat. CRL-2638, ATCC) and Luc-MC26 (carrying firefly luciferase, from the Kenneth K. Tanabe Laboratory, Massachusetts General Hospital) were cultured in RPMI-1640 (cat: R8758, Sigma) and ATCC-formulated Dulbecco's Modified Eagle’s Medium DMEM (cat.10-013-CM, Corning), respectively. The CT26 and Luc-MC26 cell lines were declared pathogen-free after being subjected to murine pathogen testing by the Diagnostic Laboratory of the Division of Comparative Medicine (MIT). Expi293F cells (cat. A14527, Thermo Fisher Scientific) for antibody expression were maintained in Expi293 Expression Medium (cat. A1435101, Thermo Fisher Scientific) at densities of 0.25-6x10^{6} cells/mL in a humidified atmosphere of 95% air and 8% CO_2 at 37°C.

In vitro macrophage mononucleosome uptake inhibition assay with liposomes:
J774A.1 and RAW264 cells were plated at densities of 30,000 and 45,000 cells/chamber, respectively, in 8-well chamber slides (cat.80806, Ibidi). Following overnight acclimatization, cells were incubated with 300 µL of liposomes (SPE, DSPG, or DSPC) diluted in DMEM at the desired concentrations (0.1-5 mg/ml) for 4 h at 37°C. Next, 30 µL of mononucleosomes were spiked into each well to achieve a final mononucleosome concentration of 10 nM and further incubated for 2 h at 37°C. Cells incubated with DMEM followed by mononucleosome addition were used as a positive control for uptake, and cells incubated only with DMEM were used as a negative control. At the end of the incubation, cells were washed once with DMEM, stained with Hoechst 33342 (cat. H3570, ThermoFisher) 1:2000 in DMEM for 10 min, and further washed (twice with DMEM and once with PBS) to remove any extracellular mononucleosomes. Subsequently, cells were fixed with 4% PFA for 20 min and washed with PBS prior to imaging on an Eclipse Ti microscope (Nikon).

To quantify cellular uptake, 4 fields of view per well were obtained at 10X magnification and mean Cy5 fluorescence intensity per cell were quantified using custom scripts in QuPath (*73*). Results are displayed after background subtraction using the mean Cy5 fluorescence intensity per cell from the negative control. In parallel, quantification was also performed using flow cytometry by pooling cells from 2 wells per condition. To harvest cells for flow cytometry, cells were trypsinized with 50 µL of trypsin/well (5 min, 37°C) and quenched with 100 µL of DMEM. Cells were scraped off the well and resuspended in 1 ml of PBS. Cells were pelleted (350 *g*, 5 min, 25°C) and fixed with 100 µL of 4% PFA (20 min, 25°C). Cells were then washed with 1 mL of FACS buffer (2% BSA in PBS) and resuspended in 400 µL of FACS buffer prior to analysis in a LSRII-HTS flow cytometer (BD Biosciences). Excitation was performed with the 640 nm laser and emission measured with the APC-A filter. Gating was performed to include only vertical and horizontal singlets. After gating, the percent positive cells was calculated as the fraction of cells above the APC value that marked the lower limit of the distribution for the positive control (treated with NCP only).

In vitro cell viability and liposome uptake studies in macrophages:
J774A.1 cells were seeded at 10,000 cells/well and RAW264 cells at 12,000 cells/well into two 96-well plates with transparent bottom. To measure liposome uptake, 24 h after seeding, cells were incubated with Cy7-SPE liposomes in DMEM at concentrations of 0-5 mg/mL for 4 h and washed three times with PBS. Liposome uptake was then measured using an Infinite F200 Pro reader (Tecan) at 750/785 nm. To measure cell viability, 24 h after seeding, cells were incubated with SPE liposomes at concentrations of 0-5 mg/mL in DMEM containing the viability dye from the RealTime-Glo MT Cell Viability Assay (cat: G9712, Promega). After incubation with liposomes for 4 h, endpoint cytotoxicity analysis was performed by measuring luminescence using an Infinite F200 Pro reader (Tecan) fluorometer. Percent viability was calculated relative to untreated cells in DMEM.

In vitro E. coli uptake study in macrophages with liposomes:
J774A.1 cells were seeded at 10,000 cells/well into 96-well plates (Greiner #655892). After 48 hours, cells were incubated with SPE liposomes (50mg/mL) at 1:10, 1:20, and 1:50 dilution conditions for 1 hour in a cell culture incubator at 37°C with 5% CO_2. Alexa 488 conjugated Escherichia coli (K-12 strain) BioParticles (cat. E13231, ThermoFisher) was added at 10 µg/well.

After 1 h incubation at 37°C with 5% CO_2, cells were fixed with 4% paraformaldehyde at room temperature for 20 min and treated with 1x cell permeabilization buffer (cat. 00-8333-56, Invitrogen) for 10 min. Cells were stained with Hoechst 33342 (1:5000, cat. H3570, ThermoFisher) and Phalloidin-Alexa 594 (1:2000, cat. 20553, Cayman) for 30 min at room temperature. Cells were washed 5 times with 250 μL PBS per well and hydrated with 50 μL PBS per well. Images were taken using the Opera Phenix Imaging System (Perkin Elmer), and uptake of the *E. coli* bioparticles per cell was quantified automatically using the Harmony PhenoLOGIC software (Version 9.0, Perkin Elmer).

<u>Animal models</u>

All animal studies were approved by the Massachusetts Institute of Technology Committee on Animal Care (MIT Protocols 042002323, 2301000462). Animals were maintained in the Koch Institute animal facility with a 12 h light/12 h dark cycle at 18-23°C and 50% humidity. All animals received humane care, and all experiments were conducted in compliance with institutional and national guidelines and supervised by staff from the Division of Comparative Medicine of the Massachusetts Institute of Technology. Female BALB/c mice (6-10 weeks, Taconic Biosciences) were used for all healthy mouse experiments. To generate the CT26 flank tumor model, female BALB/c mice (6 weeks, Taconic Biosciences) were injected subcutaneously with $2x10^6$ CT26 cells resuspended in Opti-Mem (cat. 11058021, Thermo Fisher) into bilateral rear flanks. Tumors were measured every other day for 2 weeks, and tumor volumes were calculated by the modified ellipsoidal formula: $V = 0.5 * (l * w^2)$, where l and w are the tumor length and width, respectively. To generate the transplantation model of lung metastasis, 1x106 Luc-MC26 cells in 100 μL DPBS were injected intravenously (i.v.) into female BALB/c mice (6 weeks, Taconic Biosciences). Tumor growth was monitored by luminescence using the In Vivo Imaging System (IVIS, PerkinElmer) on days 6, 13, and 20 after tumor inoculation.

<u>Blood collection</u>

Retro-orbital blood draws (70 μL in general, 35 μL for antibody pharmacokinetic study) were collected via non-heparinized capillary tubes from mice under isoflurane anesthesia, alternating between eyes for serial draws. Mice were allowed to recover from anesthesia between blood draws. Blood was immediately displaced from capillary tube into 70 μL of 10 mM EDTA (cat. AM9260G, Thermo Fisher Scientific) in PBS. For terminal bleed samples, blood was collected via cardiac puncture into a syringe filled with 200 μL of 10 mM EDTA in PBS. Total volume was measured and additional 10 mM EDTA in PBS was added to reach a 1:1 ratio of blood to EDTA. Blood with EDTA was kept on ice and centrifuged within 90 minutes at 8000 *g* for 5 minutes at 4°C. The plasma fraction was collected and stored at -80°C until further processing.

<u>cfDNA extraction and quantification:</u>

Frozen plasma was thawed and centrifuged at 15,000 *g* for 10 minutes to remove residual cells and debris. 1x PBS was then added into plasma to make the total volume 2.1 ml for cfDNA extraction using the QIAsymphony Circulating DNA kit (cat:937556, Qiagen). The extracted cfDNA was quantified using a qPCR assay and then frozen at -20°C until ready for further processing. Plasma cfDNA concentration was quantified using a Taqman qPCR assay targeting a locus on mouse genome.
Forward primer: GGGACTCCTGCAGATCGTTA;
Reverse primer: ATCTGGCCCTATCTTCCATCCT;

Taqman probe: /56-FAM/CCTGTGGTG/ZEN/CTGAACCTATCAACAGCA/3IABkFQ/.

In vivo mononucleosome pharmacokinetic study

SPE-liposomes or sterile DPBS were administered i.v. into awake mice (50-300 mg/kg, 200 µL). 30 min after liposome injection, 1 µg recombinant mononucleosomes carrying the Widom601 (W601) sequence (cat. 81070, ActiveMotif) suspended in 10 µL DPBS were injected i.v. into anesthetized mice. In the study evaluating the % exoNCP remaining at 60 min (n=4 per group), 70 µL of blood was drawn retro-orbitally 1 min and 60 min after mononucleosome injection. For the mAb pharmacokinetic assay, 10-20 ng of W601 sequence (cat. 81070, ActiveMotif) was combined with antibody in 200 µL of PBS. Engineered variants were produced in-house; 35I9 was purchased from Abcam (cat. ab27156); mouse IgG2a control (clone 20102, cat. MAB003), anti-FcγRII/III (rat anti-mouse, clone 190909, cat. MAB1460), and anti-FcγRI (rat anti-mouse, clone 29035, cat. MAB2074) were purchased from R&D systems. 40 µg of anti-FcγRII/III and 20 µg of anti-FcγRI was used in FcγR-blocking conditions. Each mouse was anesthetized with inhaled isoflurane and injected i.v. with 200 µL of mixture. At 1 minute after injection, 70 µL of blood was collected via a retro-orbital blood draw. Mice were allowed to recover after this and between subsequent blood draws (all 70 µL). % W601 remaining was calculated as the % W601 remaining at 60 min relative to 1 min, as quantified using Taqman qPCR (forward primer: 5’CGCTCAATTGGTCGTAGACA, reverse primer: 5’TATCTGACACGTGCCTGGAG and Taqman probe: /56-FAM/TC TAG CAC C/ZEN/G CTT AAA CGC ACG TA/3IABkFQ/).

Liposome biodistribution study

100 mg/kg Cy7-SPE-liposomes (200 µL in sterile DPBS) were administered i.v. into awake mice. For ex vivo organ imaging, 1 h after liposome administration mice were euthanized and liver, spleen, lungs, kidneys, and heart were harvested (n = 4 mice per group). A PBS-treated mouse was used as a negative control to measure organ autofluorescence. Organ fluorescence was measured using the 800 nm filter of an Odyssey CLx instrument (Li-Cor). Biodistribution was quantified as the % of total fluorescence across all organs for each mouse. For in vivo liposome biodistribution studies (n = 1 mouse per group), 50-300 mg/kg Cy7-liposomes (200 µL in sterile DPBS) were administered i.v. in awake mice. Accumulation of liposomes in the liver and spleen was measured using the In Vivo Imaging System (IVIS, PerkinElmer) by defining regions of interest covering the upper abdomen of mice.

Plasma cfDNA concentration measurements following liposome administration:

100 mg/kg or 300 mg/kg SPE-liposomes (200 µL in sterile DPBS) or DPBS were administered i.v. in awake mice (n = 3 mice per group). 1 min, 30 min, 1 h, 3 h, 5 h, and 24 h after liposome administration, 70 µL of blood was collected retro-orbitally. Only 2 blood samples were collected from each mouse, to prevent repeat sampling from the same capillary bed. Plasma cfDNA concentration was quantified as described in “cfDNA extraction and quantification”. Given that cfDNA yields reached maxima at 30 min and 3 h for the 100 mg/kg and 300 mg/kg doses, respectively, we decided to sample blood 1 h after liposome administration, which allowed us to compare results from animals treated with different liposome doses in our tumor models. Furthermore, on the prospect of clinical translation, a 1 h wait would be compatible with more streamlined incorporation into clinical practice.

Macrophage depletion using liposomal clodronate

Mice were treated with 56 mg/kg liposomal clodronate (Clophosome-A, cat: F70101C-A, Liposome Expert) or PBS to deplete macrophages in the liver and spleen. 2 days after, the in vivo mononucleosome pharmacokinetic study, as described above, was repeated in clodronate-naive (intact macrophages) and clodronate-treated (depleted macrophages) mice (n = 4-5/group).

DNA-binding antibodies, ELISA, and BLI

Mouse IgG antibodies against DNA were obtained from commercial vendors – 404 (cat. sc-66081, Santa Cruz Biotechnology), 1.BB.27 (cat. sc-73064, Santa Cruz Biotechnology), 3H12 (cat. sc-73065, Santa Cruz Biotechnology), HYB331-01 (cat. sc-58749, Santa Cruz Biotechnology), 121-3 (cat. ab270732, Abcam), 35I9 (cat. ab27156, Abcam), rDSD/4565 (cat. ab273137, Abcam), AE-2 (cat. MA1-35346, Thermo Fisher Scientific). Antibodies were tested for binding to dsDNA using a mouse anti-dsDNA IgG ELISA kit per the manufacturer's instructions (cat. 5120, Alpha Diagnostic International). Biolayer Interferometry was performed using SA biosensors (cat. 18-5019, Sartorius) with a 90-bp biotinylated dsDNA oligonucleotide (5'-CATAAAGTCCAACCAGTCACAAACCCTTTGATATACAATGGTGTCCAGCCTGCAGGATATAATGGTGCAATAGTGGCATGAGAGTTGTGG from mm9 Chromosome 1) and antibodies in 1x kB buffer (PBS, 0.5% w/v BSA, 0.05% v/v Tween) at 37°C with the following assay times: Baseline1 150 s, Loading 300 s, Baseline2 150 s, Association 500 s, Dissociation 600 s.

Electrophoretic mobility shift assays (EMSA)

Widom601 dsDNA complexed with recombinant human histones was purchased from Epicypher (cat. 16-0009). Free Widom601 dsDNA was amplified from a purchased template (cat. 18-0005, Epicypher) using primers 5'-CTGGAGAATCCCGGTGC and 5'-ACAGGATGTATATATCTGACACGTGC. Double-stranded DNA (free and/or histone bound) was combined at a final concentration of 4 ng/µL total DNA with varying concentrations of 35I9 in PBS (21-040-CM, Corning) in 10 µL total. 1 µL of Novex high density TBE sample buffer (cat. LC6678, Thermo Fisher Scientific) was added and 10 µL of mixture was loaded into 6% DNA Retardation Gels (cat. EC6365BOX, Thermo Fisher Scientific). Gels were run at 4°C, 100 V for 120 minutes in 0.5x TBE, stained with SYBR Green (cat. S7567, Thermo Fisher Scientific) at 1:10000 dilution in 0.5x TBE for 30 minutes and imaged on an ImageQuant LAS4000.

DNase protection assays

To measure sensitivity to DNase digestion, the DNaseAlert kit (cat. 11-02-01-04, IDT) was used in combination with various concentrations of recombinant DNase I and antibody 35I9 in 100 µL reactions incubated at 37°C in a Tecan microplate-reader with initial measurement before addition of DNase I and subsequent measurements every 5 minutes after addition of DNase I (excitation 365 nm, emission 556 nm).

Immunoblot analysis

EMSA gels, after imaging for dsDNA with SYBR Green, were transferred to nitrocellulose membranes using the iBlot2 dry transfer system (Thermo Fisher Scientific) with transfer voltage of 23V for 6 minutes. Membranes were blocked by incubating with 5% milk in tris-buffered saline + 0.1% Tween (TBST-M) for 1 hour at room temperature with gentle rocking. Membranes were then incubated with HRP-conjugated primary antibody against human histone H3 (cat. ab21054, Abcam) at 1:2000 dilution in TBST-M for 2 hours at room temperature. After incubation,

membranes were washed with tris-buffered saline + 0.1% Tween (TBST) three times (5 minutes each) with gentle rocking followed by a final 5-minute wash in tris-buffered saline (TBS). Bound antibody was detected with Novex ECL chemiluminescent substrate (cat. WP20005, Thermo Fisher Scientific).

Antibody expression and purification

Desired Fc mutations were introduced into the heavy chain sequence (as determined by LC-MS de-novo sequencing, Rapid Novor Inc) and codon-optimized for expression in HEK293 cells. Gene-blocks for the heavy and light chain (IDT) were cloned into the same gWiz plasmid, separated by the T2A ribosome skipping sequence (*79,80).* Expi293F cells at a density of $3x10^6$ cells/mL were transfected with 1 mg/L of culture of plasmid complexed with PEI Max 40K (cat. 24765-100, Polysciences) in a 1:2 plasmid:PEI w/w ratio in 40mL Opti-MEM (cat. 31985062, Thermo Fisher Scientific) per 1 L culture. Flasks were kept in a shaking incubator (125 rpm) at 37°C and 8% CO_2. 24 h after transfection, flasks were supplemented with glucose and valproic acid (cat. P4543, Millipore Sigma) to final concentrations of 0.4% v/v and 3 mM, respectively. Culture supernatant was harvested after 5-6 days and purified using Protein A affinity chromatography (AKTA, Cytiva), buffer exchanged into PBS and sterile-filtered.

Antibody pharmacokinetics and biodistribution

To label antibodies, the amine-reactive fluorophore AQuora 750 (cat. AQ-111960LF, Quanta Biodesign) was incubated with 100 µL aliquots of 1.3 mg/ml aST3 or 1.0 mg/ml 35I9, at 8 dye:1 protein and 18 dye:1 protein molar ratio, respectively, for 2.5 h at room temperature. Excess dye was removed by washing conjugate with sterile DPBS 10 times using 30 kDa Amicon filters (cat. UFC503024, EMD Millipore) at 12,000 rpm, 2.5 min, 4°C. After concentrating the volume of the last wash to approximately 200 µL, a Nanodrop spectrophotometer (Thermo Fisher Scientific) was used to measure the protein yield (absorbance at 280 nm) and the fluorophore concentration and degree of labeling (absorbance at 740 nm) to confirm comparable degree of labeling for both proteins (aST3: 2.8 fluorophores/protein and 35I9: 3.1 fluorophores/protein). For antibody pharmacokinetic studies (n=5 per group), AQuora 750-labeled antibodies were injected i.v. at 4.0 mg/kg (200 µL in sterile DPBS) into anesthetized mice and 50 µL of blood collected from alternating eyes at 1 min, 30 min, 1 h, 2 h, 6 h, and 24 h. Abundance of antibodies in plasma samples was measured using a Tecan fluorometer 750/785 nm and presented as % remaining relative to the 1 min plasma sample. For biodistribution studies (n=5 per group), AQuora 750-labeled antibodies were injected i.v. at 4.0 mg/kg (200 µL in sterile DPBS) into anesthetized animals. 1 h after injection, mice were euthanized via isoflurane overdose and perfused with 30 mL PBS to remove blood from circulation. Liver, kidneys, spleen, heart, and lungs were harvested and imaged using an Odyssey DLx imaging system (Licor). AQuora 750-fluorescence values were normalized to organ surface area in the imaging scan as a surrogate of organ weight.

Liposome dose-titration for tumor detection in the CT26 flank-tumor model

Mice bearing bilateral CT26-flank tumors were randomized into different treatment groups (300 mg/kg, 200 mg/kg, 100 mg/kg, 50 mg/kg SPE-liposomes or PBS, n = 5-7 mice per group) at end point such that the tumor burden of the group ranged between 700-1000 mm^3. As an internal control, 70 µL blood was sampled retro-orbitally from each mouse prior to treatment. Subsequently, 50-300 mg/kg SPE-liposomes (in 200 µL sterile DPBS) or sterile DPBS were administered i.v. into awake mice. 1 h after treatment, 70 µL of blood was collected retro-orbitally

from the contralateral eye, and a terminal bleed was then performed, and blood collected for analysis. We sampled blood at 1 h given that endogenous cfDNA yields reached maxima at 30 min and 3 h for the 100 mg/kg and 300 mg/kg doses, respectively (Fig. 2E), thus allowing us to compare results from animals treated with different liposome doses in our tumor model. Furthermore, on the prospect of clinical translation, a 1 h wait would be compatible with more streamlined incorporation into clinical practice.

Performance of liposomal priming agent for tumor detection in a lung metastasis model

Six days after tumor inoculation, mice bearing Luc-MC26 metastatic tumors were randomized into different treatment groups (100 mg/kg SPE-liposomes (n = 12 mice) or PBS (n = 8 mice)) such that total burden was equivalent across different treatment groups (1.08 ± 0.5 e7 photons/s for 100 mg/kg SPE-liposomes versus 9.95 ± 5.2 e6 photons/s for PBS). To determine how our liposomal priming affected ctDNA performance at different tumor burdens, a similar workflow to that described for the CT26 model above was performed 1 week, 2 week, and 3 weeks after tumor inoculation. Namely, at each timepoint blood was collected retro-orbitally pre-treatment as an internal control and 1 h after SPE-liposome- or PBS-treatment. Additionally, at end point, terminal bleed blood was collected. cfDNA concentration measurement and ctDNA detection was performed on all samples as described below.

To calculate the sensitivity of the ctDNA test for tumor detection, mice were grouped as a function of tumor burden into those with small (total burden < 1.5e7 photons/s), medium (1.5e7 photons/s < total burden < 1.5e8 photons/s), and large (total burden > 1.5e8 photons/s) tumors. Subsequently, retro-orbital plasma samples were classified as ctDNA positive if the number of unique SNVs detected surpassed a given SNV threshold (between 2 and 10 SNVs, from lower to higher stringency of the test) and sensitivity calculated as the % of samples that were ctDNA positive per group.

Cell-line and buffy coat sequencing and fingerprint design

gDNA was extracted from CT26 and Luc-MC26 and BALB/c buffy coat, sheared, and libraries were prepared using the Kapa HyperPrep Library Construction kit (cat. KK8504, Roche Diagnostics). Whole genome sequencing was performed to 30x coverage for CT26 and Luc-MC26 and 15x coverage for BALB/c buffy coat. Sequencing data was aligned against mm9 genome assembly, SNV calling with Mutect2, and tumor fingerprint selection for a 200- and 2000-SNV panel, for CT26 and MC26, respectively as previously described (*9*). A pool of 120-bp biotinylated probes against the 200 or 2000 single-nucleotide variants (SNVs) was ordered from IDT. 98- and 1,822-SNVs validated against CT26 and Luc-MC26 gDNA, respectively (Data S1 and S2).

Library construction, hybrid capture, and sequencing

Cell-free fDNA and gDNA libraries were constructed using the Kapa Hyper Prep kit (cat: 07962363001, Roche) with custom dual index duplex UMI adapters (IDT), as previously described (9). A maximum of 50 µL or 20 ng of extracted cfDNA or 20 ng gDNA mass was used as input into library construction (LC). The prepared libraries were then quantified using the Quant-iT PicoGreen assay (cat: P11496, Invitrogen) on a Hamilton STAR-line liquid handling system. Hybrid capture (HC) using cancer cell line specific panels was performed using the xGen hybridization and wash kit (cat: 1080584, IDT) with xGen Universal blockers (cat: 1075476; IDT). For the ctDNA diagnostic test, libraries were pooled up to maximum 12-plex, with a library mass

equivalent to 25 times DNA mass into LC for each sample, and 0.56 pmol/µL of a panel consisting of 120 bp long probes (IDT) targeting cancer cell line-specific SNVs were applied. After the first round of HC, libraries were amplified by 16 cycles of PCR and then carried through a second HC but with half volumes of human Cot-1 DNA, xGen Universal blockers, and probes (note: we tested mouse Cot-1 DNA and did not observe any impact on the assay performance). After the second round of HC, libraries were amplified through 8-16 cycles of PCR, quantified, and pooled for sequencing (151 bp paired-end runs) with a targeted raw depth of 40,000 x per SNV locus for 20 ng DNA input. Sequencing data was processed by our duplex consensus calling pipeline as previously described, yielding measurements of the total number of mutant duplexes detected, the number of distinct SNVs detected, and the tumor fractions detected as previously described (*9*). Relative duplex depth at each SNV locus was computed by subtracting mean overall depth for the library and dividing by the standard deviation to obtain a relative duplex depth.

<u>In vitro primary murine white blood cell shedding assay</u>

Whole blood was collected from mice via cardiac puncture and erythrocyte lysed in ACK buffer (Thermo Fisher, 00-4333), per supplier's recommendation. Following centrifugation of cells at 4°C (400 *g*, 5 min), the supernatant was discarded and the white blood cell (WBC) pellet resuspended in RPMI medium. WBC were seeded at 100,000 cells/well into 96 wells and left to adhere for 30 min. After this, medium was discarded and RPMI supplemented with SYTOX-green nucleic acid dye (Thermo Fisher, S7020) and 0, 1.2 or 5 mg/ml SPE-liposomes was added to cells (200 µL working volume, n=4 wells/condition). 2 hours after the incubation, 150 µL supernatant per well were centrifuged for at 4°C (15,000 *g*, 15 min) and 100 µL of supernatant plated into a new 96-well plate. Fluorescence was measured using an Infinite F200 Pro reader (Tecan) at 488/523 nm.

<u>Toxicity assessment of nanoparticle priming agent</u>

100 mg/kg or 300 mg/kg SPE-liposomes (in 200 µL sterile DPBS, n = 3 mice per group) or sterile DPBS (n = 3 mice per group) were injected i.v. into awake mice once a week for 3 weeks. Weight measurements were performed every other day from the first injection to 1 week after the last injection. 1 week after the last injection, terminal bleed blood was collected, and serum was used for biochemical analysis using the IDEXX Chem21 panel (performed through the Division of Comparative Medicine Laboratory at the Massachusetts Institute of Technology). Additionally, heart, lungs, liver, spleen, and kidney were harvested, fixed, paraffin-embedded, sectioned, and stained with hematoxylin and eosin and assessed by a veterinary pathologist.

<u>Plasma cfDNA concentration measurements following mAb administration</u>

aST3 (40 µg in 200 µL DPBS) was administered into anesthetized mice (n = 4 mice per group). 1 min, 1 h, 2 h, 3 h, and 5 h after liposome administration, 50 µL of blood was collected retro-orbitally. Only 2 blood samples were collected from each mouse, to prevent repeat sampling from the same capillary bed. Plasma cfDNA concentration was quantified as described in "cfDNA extraction and quantification".

<u>Assessing the performance of antibody priming agent for tumor detection</u>

Between days 10 and 12 post-tumor inoculation, the performance of aST3 on ctDNA testing was assessed in Luc-MC26-tumor bearing mice. As an internal control, 70 µL blood was sampled retro-orbitally from each mouse prior to treatment. Subsequently, 4.0 mg/kg aST3 (in 200 µL sterile

DPBS) or 4.0 mg/kg IgG2a isotype were administered into awake mice i.v. 2 h after treatment, 70 μL of blood was collected retro-orbitally from the contralateral eye and mice exsanguinated via cardiac puncture to collect the remainder of blood for analysis. The 2 h timepoint was chosen as it resulted in the highest endogenous cfDNA concentration in healthy mice after injection of aST3 (Fig. S27). cfDNA concentration measurement and ctDNA detection was performed on all samples as described below.

Sensitivity estimation

To estimate sensitivity at smaller panel sizes, we applied a bootstrap procedure down-sampling with replacement from our 1822-SNV panel to smaller panel sizes. Sensitivity at different detection thresholds was estimated as the fraction of mice that had mutant molecules detected at the given threshold. For each panel size and dose, 100 replicates were generated, and the mean sensitivity and standard error was computed. To estimate sensitivity at lower tumor fractions, we first confirmed that the distribution of mutant molecules (n^{-}_{ij}) and the distribution of the ratio of mutant molecules to total molecules (n^{-}_{ij}/t_{ij}) could be accurately recapitulated via a binomial sample n^{-}_{ij} ~ Binom(t^{--}_{Ij}, f_i) where n_{ij} is the number of mutant molecules at SNV locus j in sample i, t_{ij} is the number of total molecules at SNV locus j in sample i, and f_i is the global tumor fraction in sample i. To estimate sensitivity at lower tumor fractions, we then generated distributions of mutant molecules under lower f_i for each sample, also incorporating various panel sizes as above, and computed sensitivity for detection of mutant molecules under various detection thresholds. Sensitivity at each f_i, dose, and panel size was estimated by taking the mean and standard error from 100 replicates.

Identification of enriched SNV loci

The duplex depth within each dose level was normalized to mean 0 and standard deviation 1. For each SNV locus, the Pearson correlation between aST3 dose and normalized duplex depth was computed (with the control antibody group taken as dose = 0 mg/kg), and SNV loci were ordered based on the correlation measurement to identify those with relative enrichment in presence of higher antibody doses.

Analysis of overlap with DNase HS and CpG sites

We downloaded DNase HS peaks for all leukocyte and myeloid datasets in mouse ENCODE (ENCFF063EHX, ENCFF125IXR, ENCFF171XTE, ENCFF185ZCW, ENCFF215CPX, ENCFF359GEV, ENCFF434GOV, ENCFF550NKM, ENCFF566TDU, ENCFF689PKR, ENCFF702OKE, ENCFF754XGR, ENCFF761OVL) and converted to mm9 coordinates using LiftOver (https://genome.ucsc.edu/cgi-bin/hgLiftOver). ENCSR000CMQ and ENCSR000CNP were excluded due to very low read depth. Although megakaryocytes have been recently recognized as important contributors to cfDNA, they were not included in the DNase-seq datasets from mouse ENCODE and could not be incorporated in this analysis (*77*). Distance between each of the 2000 SNV loci and the nearest DNase HS peak was computed, with a distance of 0 indicating overlap between the SNV locus and a peak. Coordinates of mouse CpG islands were downloaded from UCSC genome browser (table cpgIslandExt, assembly mm9). GC content of each site was computed as the percentage of G/C bases in the 120 bp probe sequence.

For genome-wide analysis of overlap with CpG and DNase hypersensitive sites, we performed whole-genome sequencing of 15 cfDNA samples - 3 samples each from mice treated with 8.0

mg/kg IgG2a control, 0.5 mg/kg aST3, 2.0 mg/kg aST3, 4.0 mg/kg aST3, and 8.0 mg/kg aST3. Samples were sequenced to a mean depth of 9x. Mean coverage by deduplicated reads was computed for CpG islands and DNase hypersensitive sites and divided by whole-genome mean coverage for each sample to obtain relative coverage of CpG islands and DNase hypersensitive sites.

Statistical analysis

One-way ANOVA was used for statistical testing unless noted otherwise. A suite of scripts (Miredas) was used for calling SNVs and creating metrics files (*9*, *15*). All other analysis was performed using GraphPad Prism v9, custom Python scripts and R (v4.0.3) (code available on Zenodo, https://doi.org/10.5281/zenodo.10237042 *(76)*). For each animal experiment, mice were randomized such that groups would have comparable tumor burden. Investigators were not blinded to the groups and treatments during the experiments.

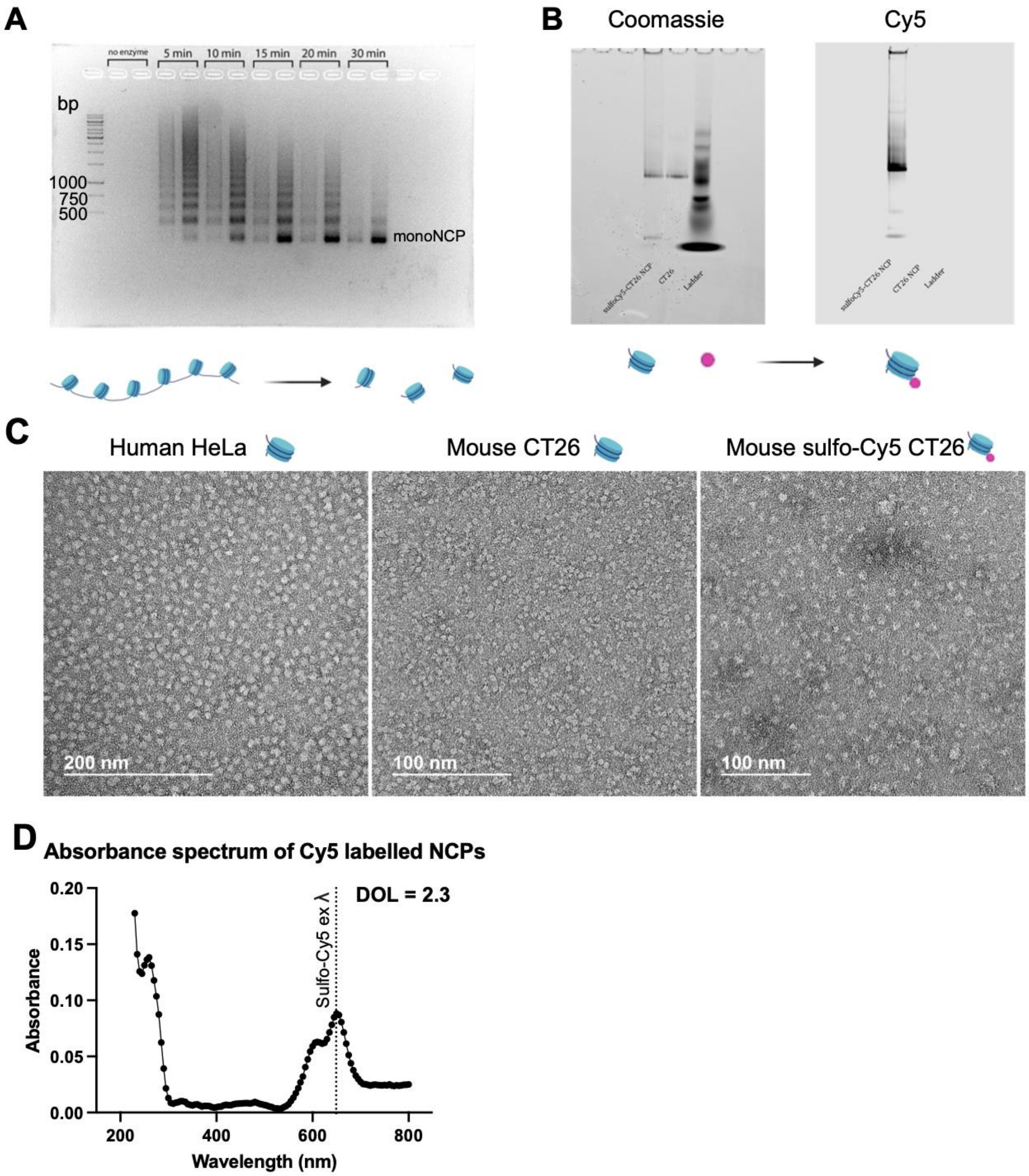

Fig. S1. Optimization of mononucleosome preparation and Cy5-labeling from the chromatin of CT26 cells. (A) Mononucleosomes (MN) were prepared by digesting the chromatin of CT26 cells for 5-30 min using the Nucleosome preparation kit (cat. 53504, Active Motif). The MN were subjected to DNA cleanup and digestion efficiency assessed via electrophoresis through a 1.5% agarose gel. A digestion time of 30 min was found to be optimal for MN preparation. **(B)** MN were labeled with sulfo-Cy5 dye (25 dye: 1 protein molar ratio, 4°C at 550 rpm overnight) and labeling confirmed by co-localization of a Cy5-positive band and a protein band in a 4-12% Tris Glycine Novex gel (100 V, 2 h, 4°C, native running buffer). **(C)** Representative TEM images of unlabeled commercial human HeLa MN (left) and murine CT26 MN prepared as described in (A) prior to (middle) and after sulfo-Cy5 labeling (right). Scale bars as indicated in images. Prepared MNs

remain stable after labeling and resemble those purchased commercially, albeit being in smaller in size [11.4 ± 1.3 nm (81 particles) for human versus 5.2 ± 0.7 nm (82 particles) for mouse, respectively, measured by the ImageJ software]. **(D)** Absorbance spectrum of Cy5-labeled MNs measured using an Infinite F200 Pro reader (Tecan) shows a peak at the excitation wavelength of the sulfo-Cy5 fluorophore (649 nm). Degree of labeling was estimated by calculating the protein concentration using a Nanodrop and the dye concentration using the Infinite F200 Pro reader to be 2.3 dye molecules per MN molecule**.**

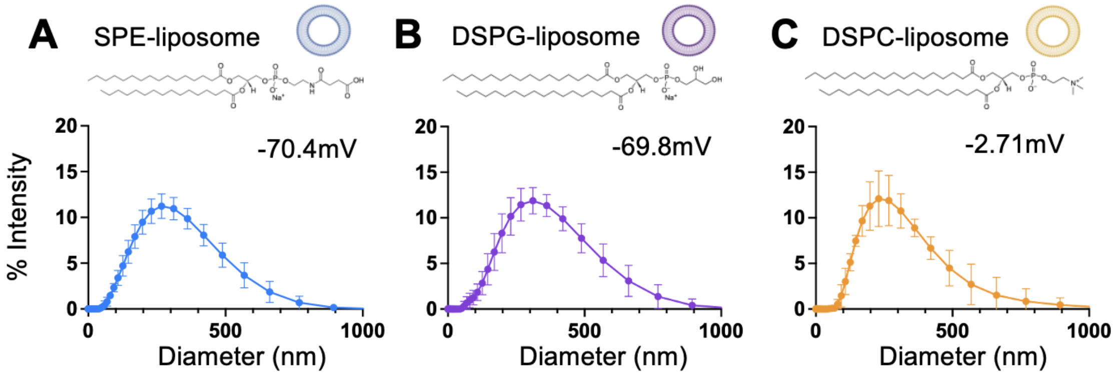

Fig. S2. Chemical structures of lipids and liposome characterization. (A) SPE: 1,2-dipalmitoyl-sn-glycero-3-phosphoethanolamine-N-(succinyl) (sodium salt), **(B)** DSPG: 1,2-distearoyl-sn-glycero- 3-phospho-(1'-rac-glycerol) (sodium salt), and **(C)** DSPC: 1,2-distearoyl-sn-glycero-3-phosphocholine. All lipids contain fully saturated fatty acid tails but SPE has a 16 carbon chain whereas DSPG and DSPC have an 18 carbon chain. Each lipid has a different head group: SPE (phosphoethanolamine-N-(succinyl), negatively charged), DSPG (phospho-(1'-rac-glycerol), negatively charged), and DSPC (phosphocholine, neutral). Histograms represent size distribution (average diameter: SPE = 234 nm ± 5.98 s.d., DSPG = 260 nm ± 3.3 s.d. and DSPC = 227 nm ± 3.3 s.d.) and embedded values zeta potential (SPE = -70.4 mV ± 1.32 s.d., DSPG = -69.8 mV± 1.0 s.d, and DSPC = -2.71 mV ± 0.6 s.d) of the three liposomal formulations, as measured by DLS.

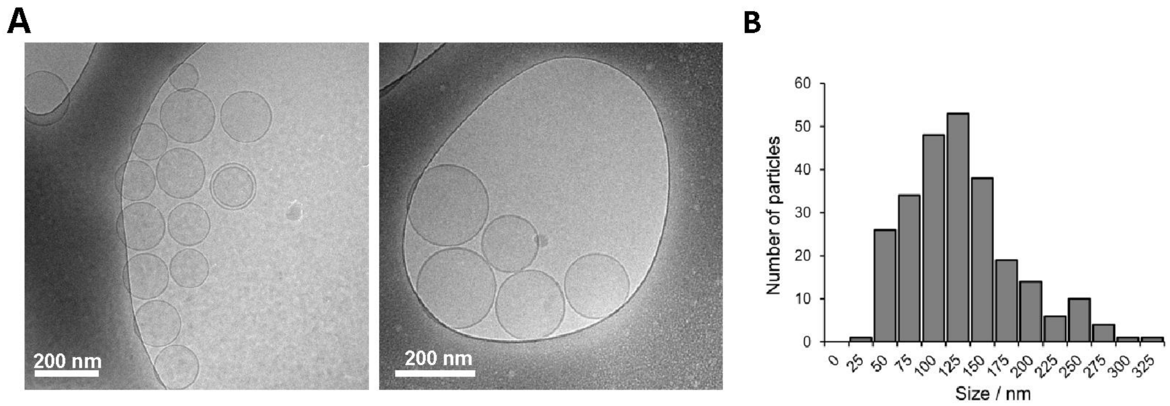

Fig. S3. Cryogenic transmission electron microscopy (cryo-TEM) images verified the vesicular morphology for SPE-based liposomes. (A) Representative images of SPE-based liposomes show the vesicular morphology of liposomes, with a dark opaque hydrophobic layer separating the inner hydrophilic lumen. Liposomes are mostly unilamellar. Scale bars as indicated in images. **(B)** Histogram of particle size was obtained by manually counting 256 particles. Each particle diameter was measured three times using the ImageJ software and the average value was recorded in the histogram.

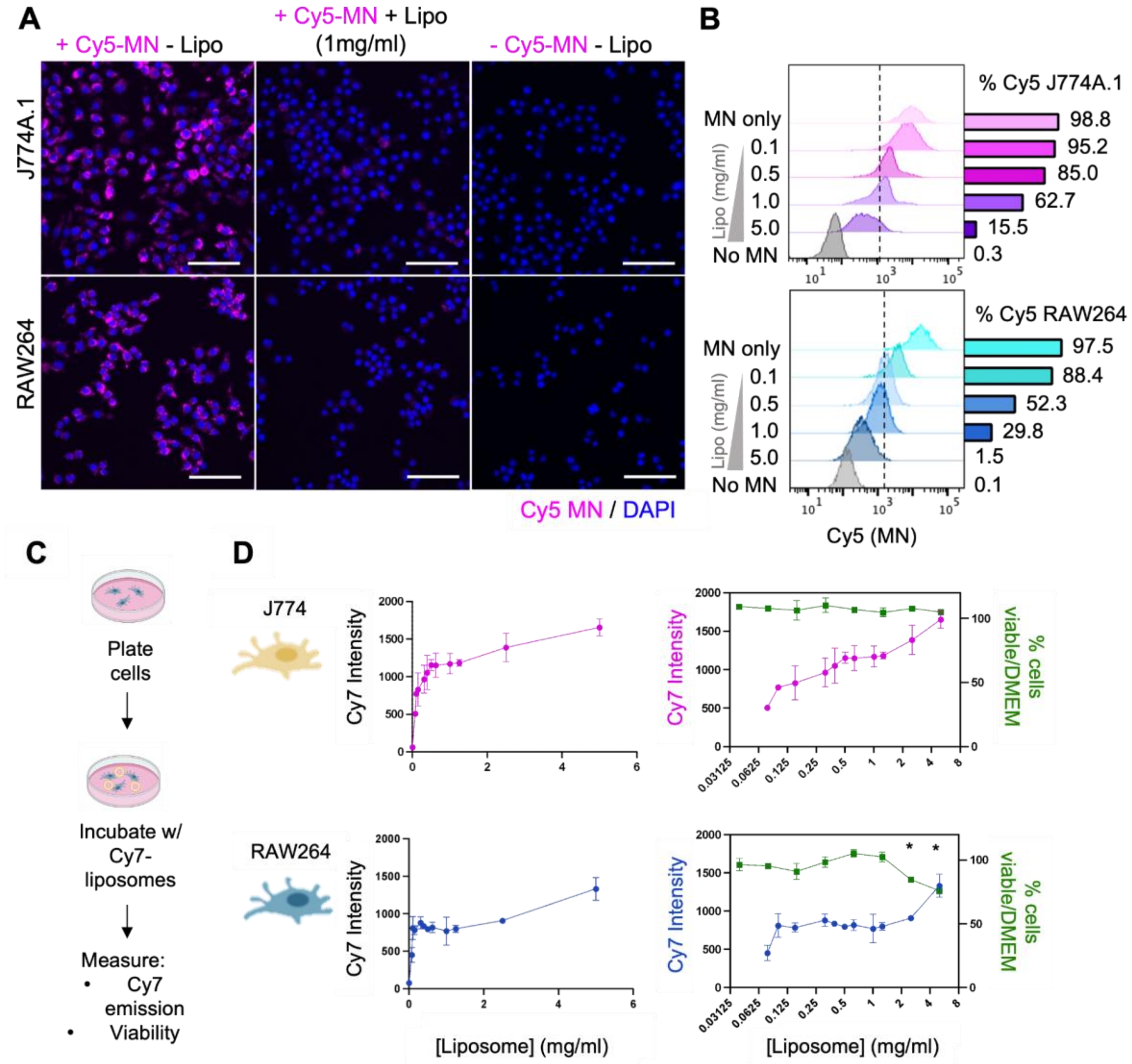

Fig. S4. SPE liposomes inhibit the uptake of mononucleosomes in two different macrophage cell lines with minimal effects on cell viability. (A) Representative epifluorescence images (and **(B)** flow cytometry-based quantification of Cy5-MN uptake by J774A.1 and RAW264 cells after liposome pre-treatment for 4 hours. **(C)** Experimental workflow to determine the viability of cells 4h post-liposome treatment and to characterize the uptake of Cy7-liposomes. **(D)** Overlay of cell viability measurements and Cy7-liposome measurements at a range of liposome concentrations. n = 3 wells per condition for A-D, * $P < 0.05$, two-tailed ANOVA**.** Scale bar = 100 µm.

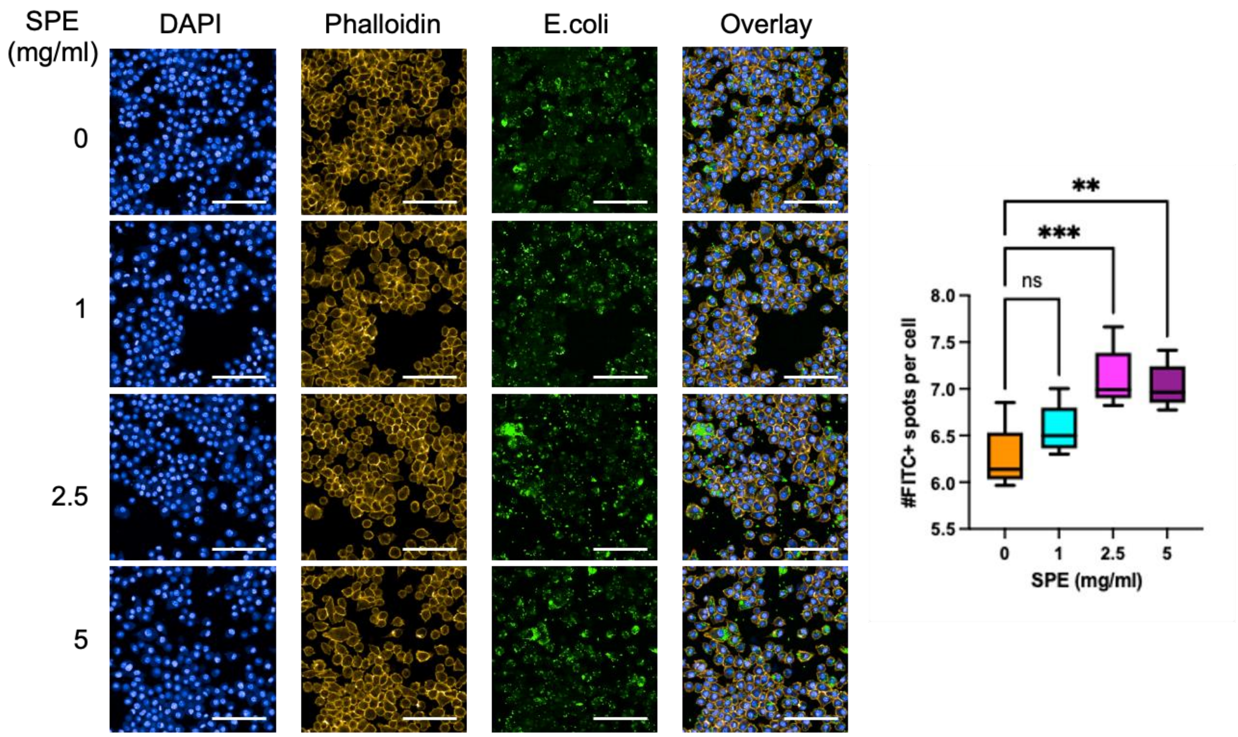

Fig. S5. SPE-liposomes do not inhibit phagocytosis of *E. coli* particles in J774A.1 cells. Representative epifluorescence images (left; scale bar = 100 μm) and image analysis-based quantification (right) of E.Coli uptake by J774A.1 cells after liposome pre-treatment for 1 hour.

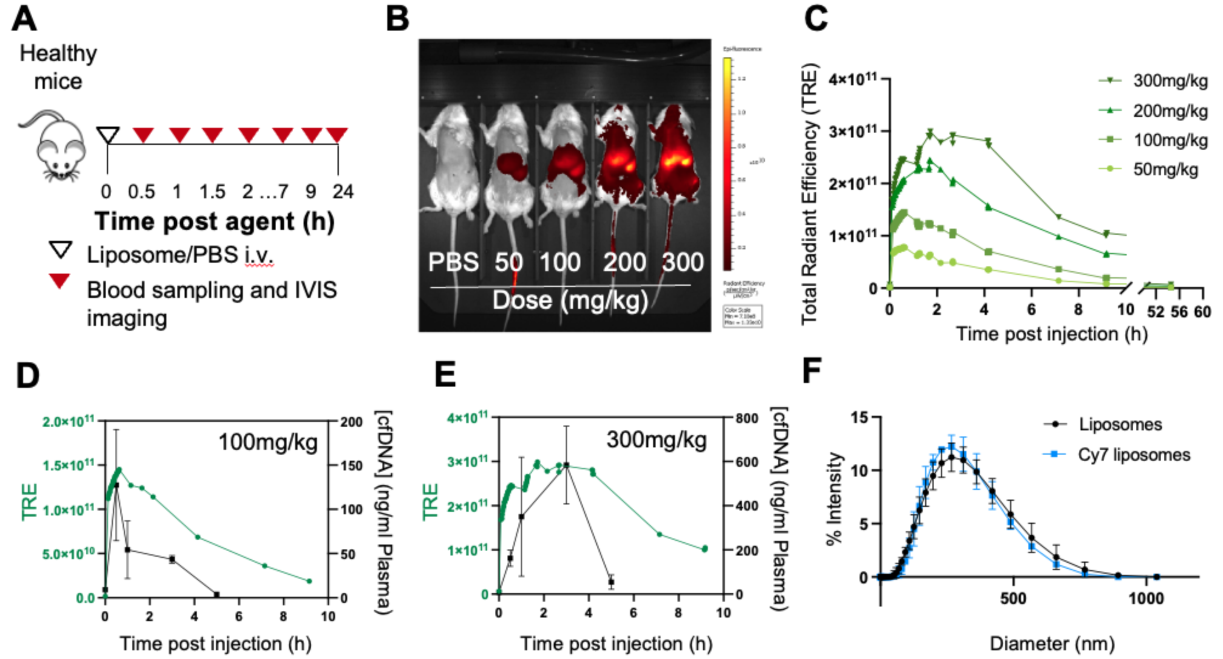

Fig. S6. In vivo in healthy mice SPE-liposomes occupy the two major organs of the MPS. (A) Experimental timeline to investigate the kinetics of liposome biodistribution and of cfDNA concentration changes in the plasma of healthy Balb/c mice following liposome administration. **(B)** Image of liposome accumulation in the liver and spleen of mice 10 min after administration. **(C)** IVIS-based quantification of liposome accumulation in the liver and spleen (as defined by a region of interest occupying the upper abdominal area of mice that overlaps with the anatomical positioning of the liver and the spleen) over time. Overlay of cfDNA plasma levels and spleen and liver accumulation (in Total Radiant Efficiency (TRE) units) after dosing liposomes at **(D)** 100 mg/kg or **(E)** 300 mg/kg showing that the maximum accumulation of liposomes in target organs is achieved prior to the peak in plasma cfDNA levels. This suggests that accumulation in MPS organs may be driving the observed trends in plasma cfDNA concentrations. **(F)** DLS-based characterization of non-fluorescent SPE liposomes (average diameter = 234 nm, PDI = 0.215) and Cy7-liposomes (average diameter = 230 nm, PDI = 0.2).

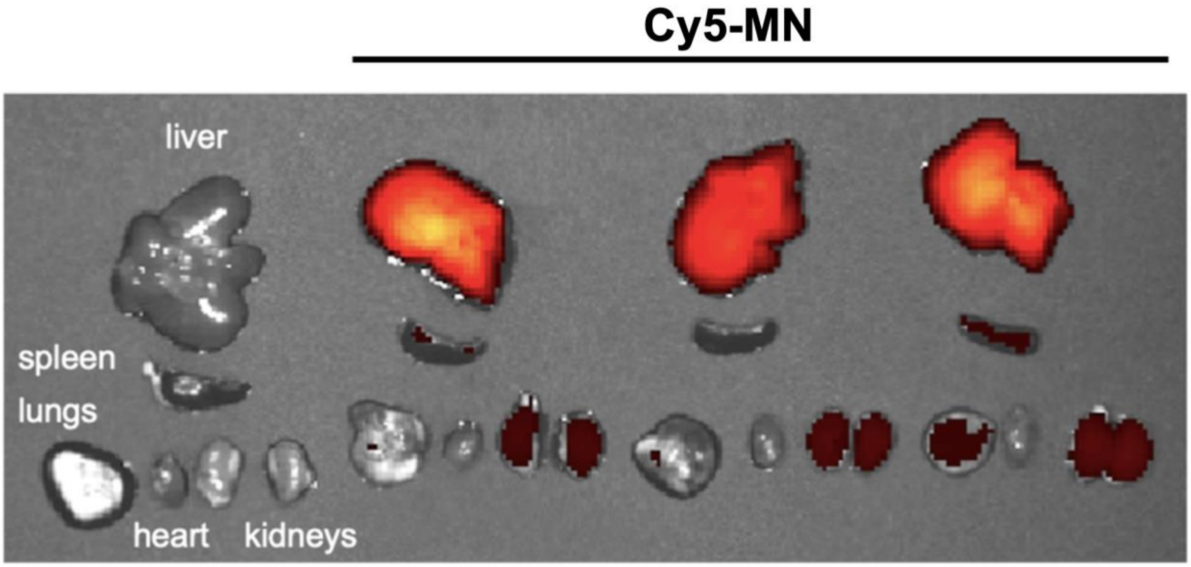

Fig. S7. Baseline organ biodistribution of mononucleosomes in healthy mice. Cy5-labeled mononucleosomes (Cy5-MN) 1 h after administration showing preferential uptake in the liver over all other organs. Left mouse was not injected and serves as a negative control.

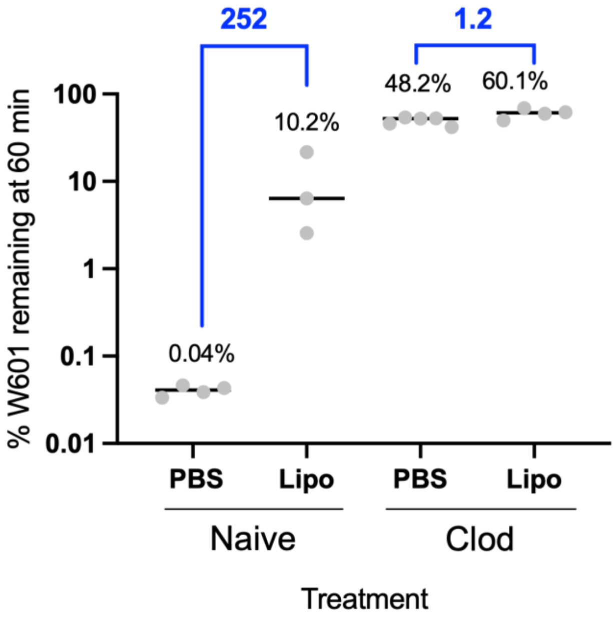

Fig S8. Macrophages are necessary for half-life extension of W601-mononucleosomes (W601-MN) following liposome administration. Mice were treated with liposomal clodronate or PBS. Two days after treatment, the plasma bioavailability of W601-MN following priming was determined. Liposomes or PBS were administered i.v. 30 min prior to i.v. administration of 1 μg of W601-MN in clodronate-naive mice (naive) vs. clodronate-treated mice (clod). The %W601 remaining in plasma 1 h after administration was determined.

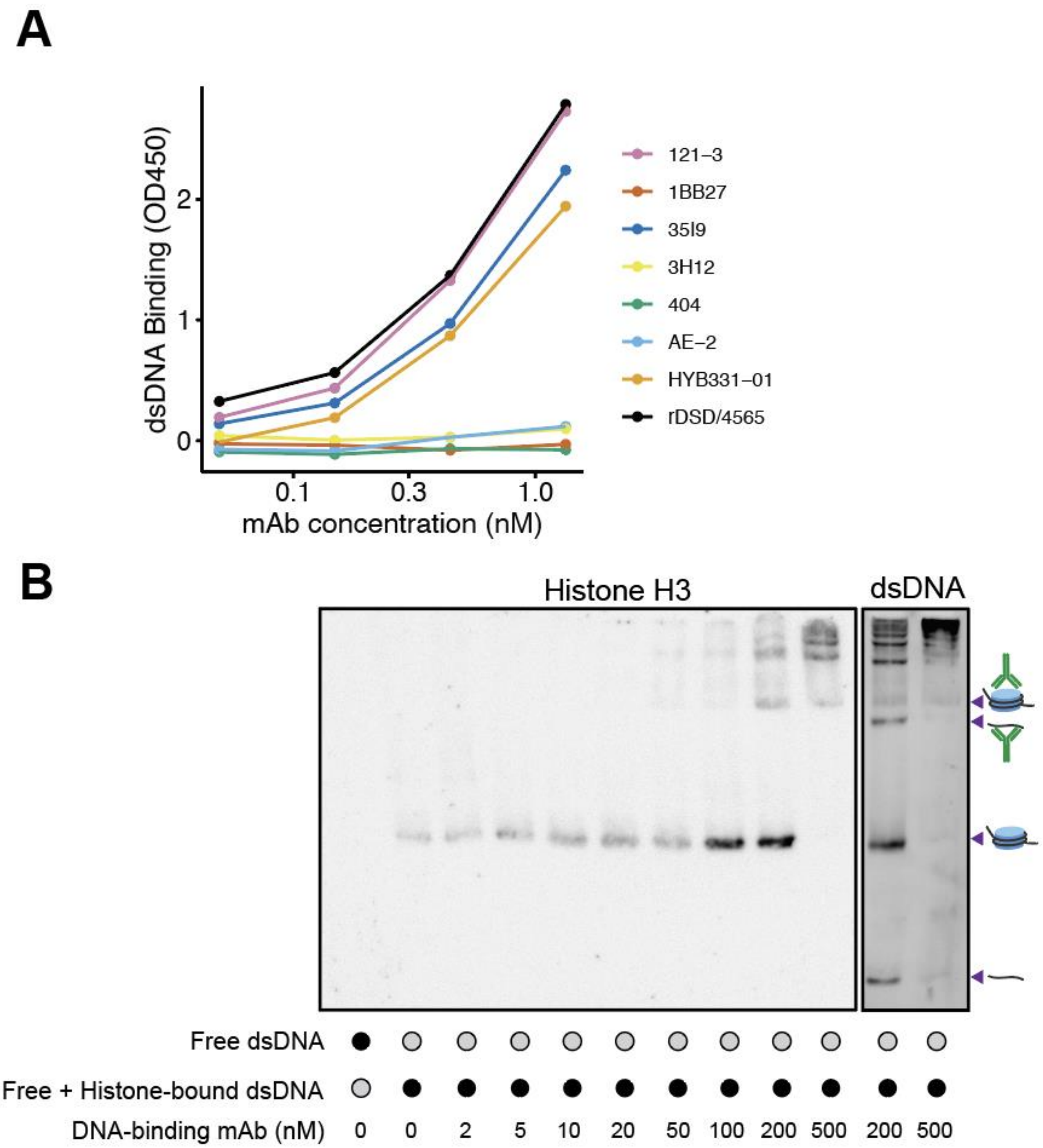

Fig. S9. Binding of anti-DNA mouse IgG mAbs to free dsDNA. (A) dsDNA binding activity via ELISA for nine DNA-binding mouse IgG antibodies. **(B)** Immunoblot of the gel in **Fig. 3A** for human histone H3. The two right-most lanes from Fig. 3A are included to facilitate comparison of bands.

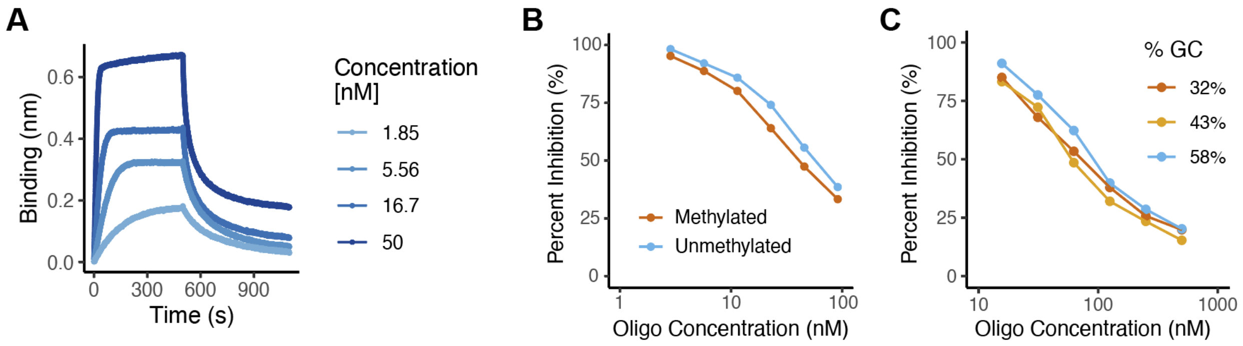

Fig. S10. Characterization of binding of mAb 35I9 to dsDNA. (A) Biolayer Interferometry (BLI) binding response of an immobilized dsDNA fragment to various concentrations of mAb 35I9. Binding affinity of 35I9 to **(B)** unmethylated versus methylated dsDNA and **(C)** dsDNA sequences of different GC content via competitive anti-dsDNA ELISA.

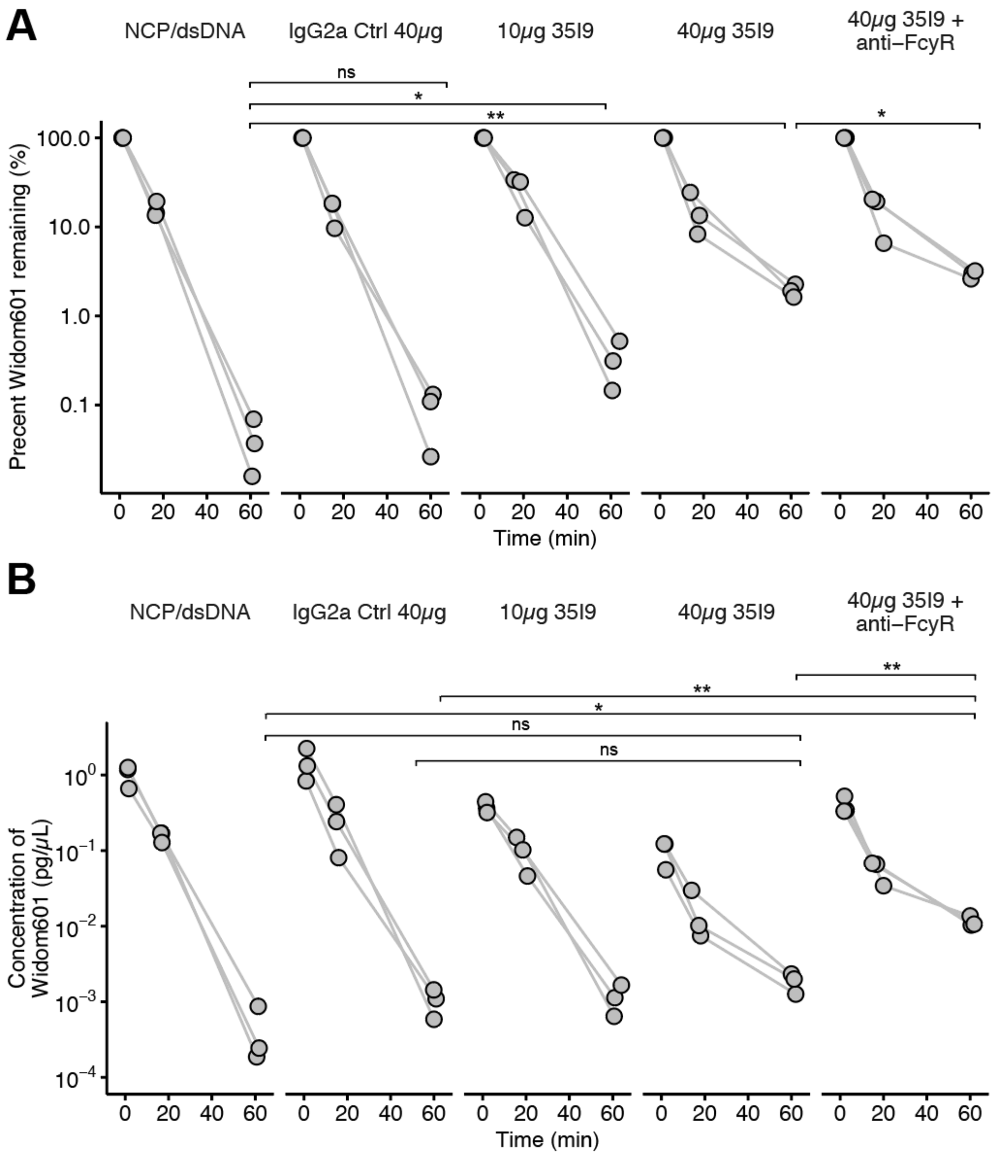

Fig. S11. Clearance of W601 over time upon intravenous administration of mAbs. (A) Percent clearance of W601 normalized to 1-minute levels. W601-mononucleosomes (W601-MN) with or without mAb (IgG2a control at 40 µg or aST3 at 10 µg or 40 µg) were injected, followed by serial blood draws and qPCR quantification of the W601 sequence in plasma. **(B)** Absolute levels of plasma W601 with or without mAb. ns - not significant, * $p < 0.05$, ** $p < 0.01$, one-way ANOVA.

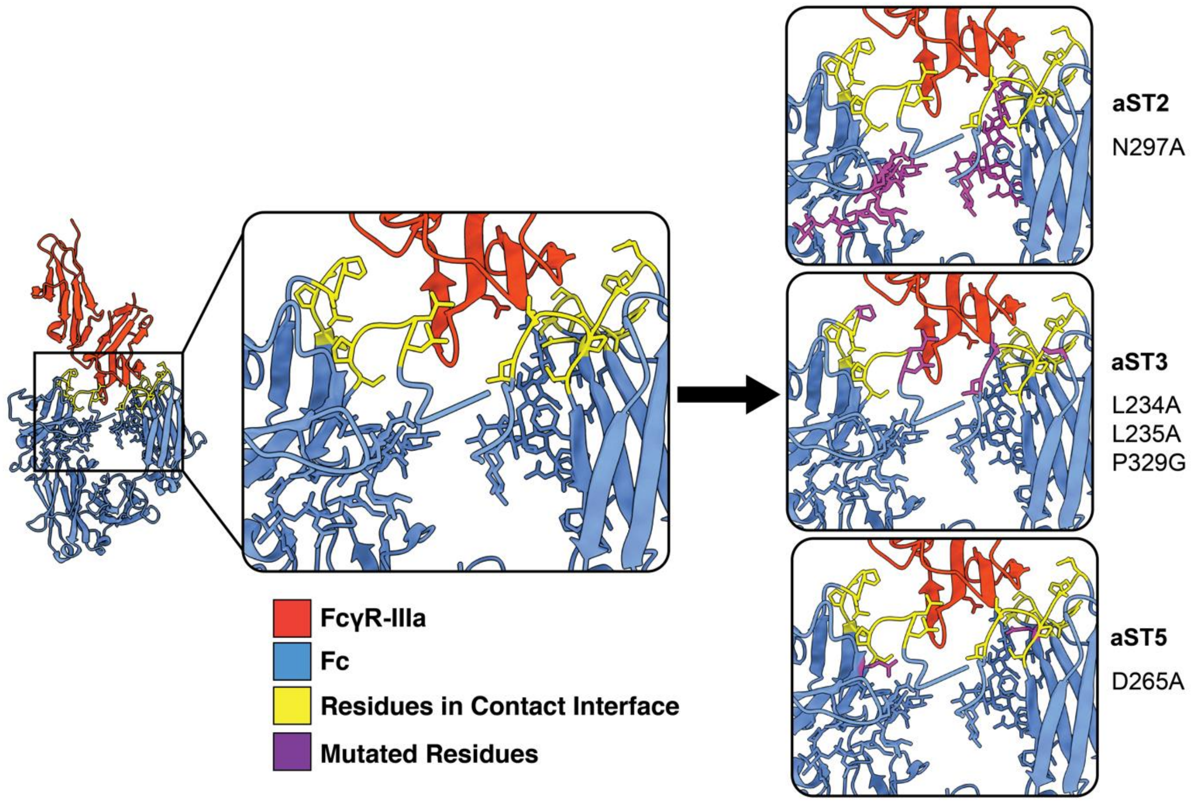

Fig. S12. Mutations disrupting interaction of Fc with FcγR-IIIa. Crystal structure of human FcγR-IIIa complexed with human IgG1 Fc (PDB: 1E4K), with residues in contact interface as identified in (*78*) highlighted in yellow. Residues mutated in Fc-engineered variants are conserved between mouse IgG2a and human IgG1, all overlap with the contact interface, and are highlighted here in purple.

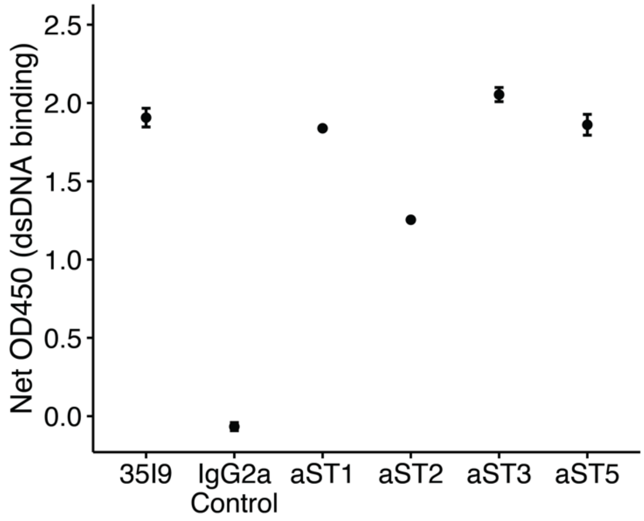

Fig. S13. Binding of engineered anti-DNA mAbs to dsDNA. dsDNA binding activity via ELISA for 35I9, unrelated IgG2a mAb, and four engineered variants. All antibodies are at 1 nM.

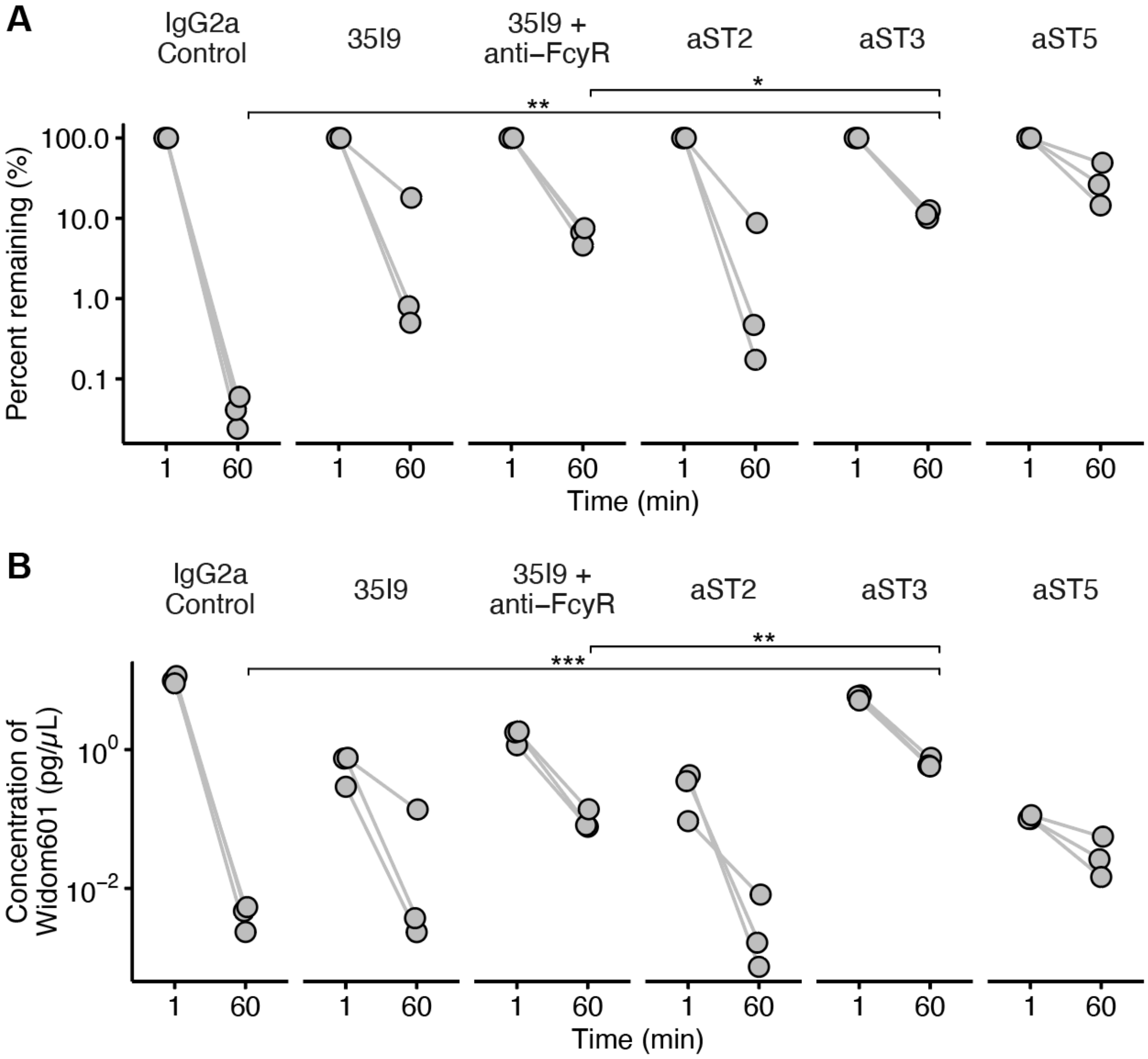

Fig. S14. Clearance of W601 from plasma after injection with various antibodies. (A) Percent of W601 remaining at 60 minutes, normalized to 1 minute. (**B**) Concentration of W601 in plasma at 1 minute and 60 minutes. IgG2a Control is an unrelated mouse IgG2a mAb. n=3 mice per treatment group. * $P < 0.05$, ** $P<0.01$, *** $P < 0.001$, one-way ANOVA.

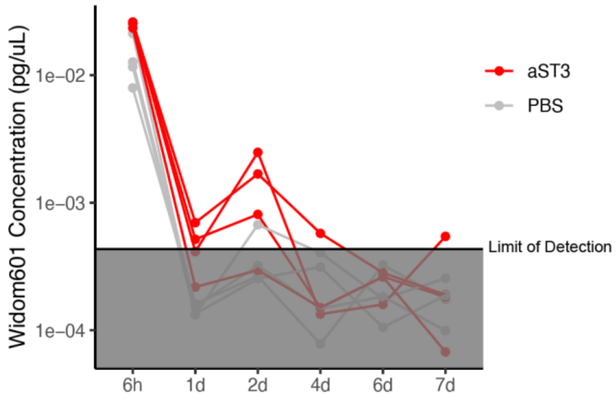

Fig. S15. Plasma clearance of W601 after the administration of W601-MN with or without mAb aST3. 20 ng W601-MN were administered i.v. with or without 40 µg of aST3 and blood sampled over time. The limit of detection is based on the Ct value that is above the average Ct value of non-template controls by 1.96 times the sum of standard deviations of non-template control and standard curve samples. Data from n=4 mice per group. h - hours, d – days.

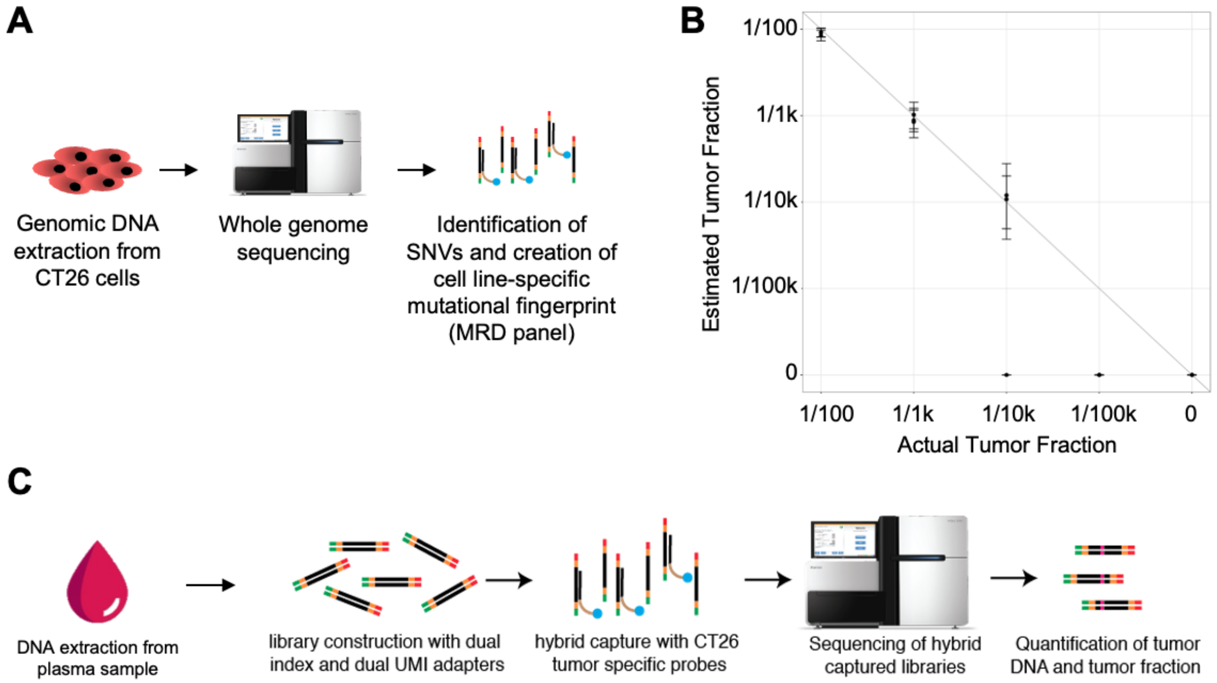

Fig. S16. Development of a ctDNA test for the CT26 bi-flank tumor model. (A) Schematic for the design of a CT26-specific tumor fingerprint panel (Data S1). **(B)** Validation of a ctDNA test tracking 98 SNVs of the CT26 bi-flank mouse model. The assay estimated tumor fractions are concordant with the actual tumor fractions down to 1:10k. Samples were prepared by serially diluting CT26 tumor gDNA into normal gDNA extracted from the buffy coat of healthy mice (n=3 biological replicates). **(C)** Schematic for the processing of plasma samples to detect SNVs. In brief, DNA was extracted from plasma samples, libraries were constructed using the Kapa Hyper Prep kit (Roche) and custom dual UMI adapters (IDT), regions of interest on the genome were captured with CT26 tumor specific SNV probes, and sequencing was performed. Following alignment and duplex consensus sequences assembly, the numbers of mutant and normal molecules were quantified, and tumor fractions were calculated using previously published methods (*9*).

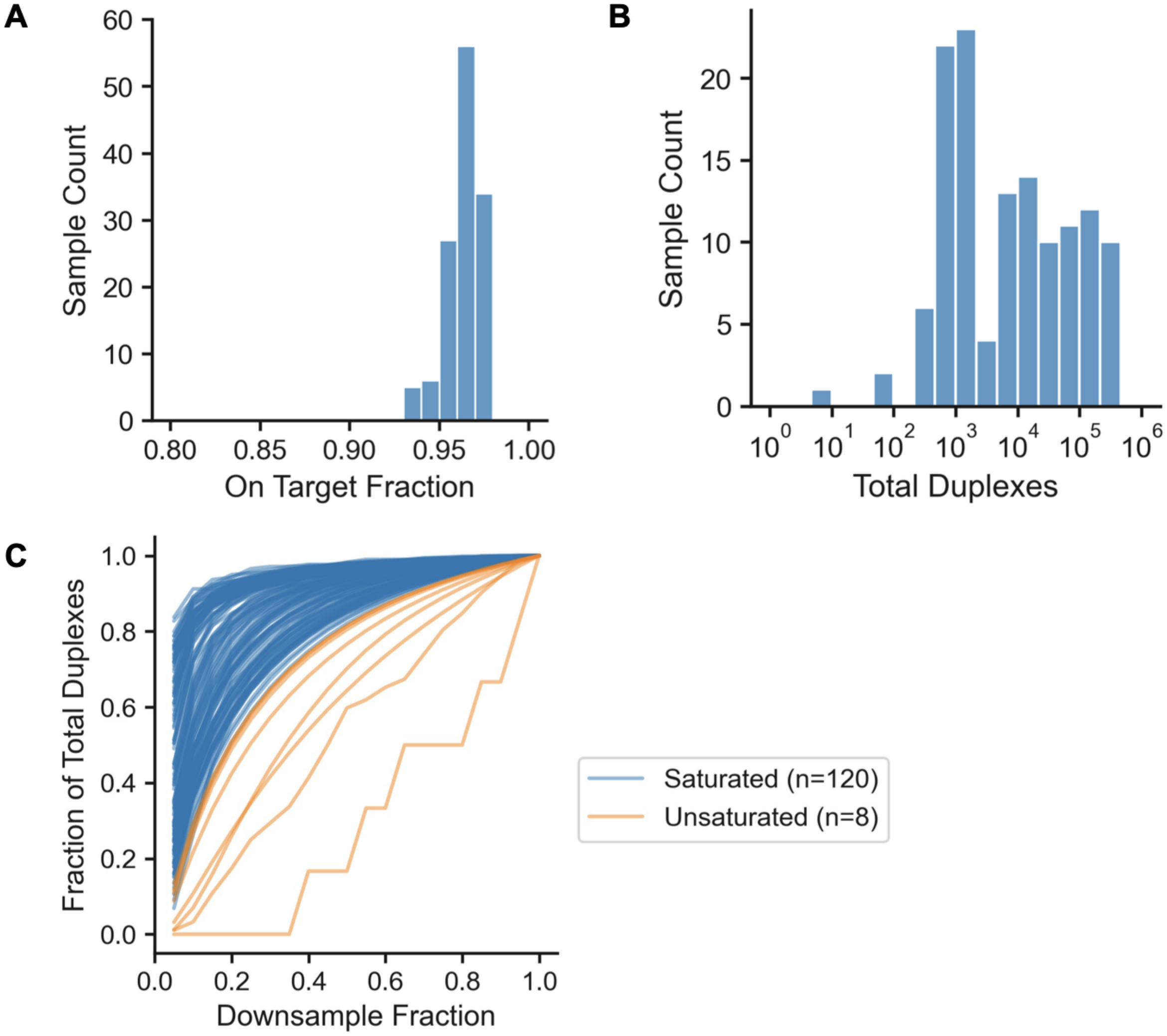

Fig. S17. Technical performance of the ctDNA diagnostic test tracking 98 SNVs in the CT26 bi-flank tumor model. Histograms of **(A)** the on-target rate and **(B)** the total number of duplexes recovered. **(C)** Fraction of total duplexes after down-sampling raw reads. We considered a sample to be saturated if it still contained at least 95% of its duplexes after down-sampling to 80% of its raw reads.

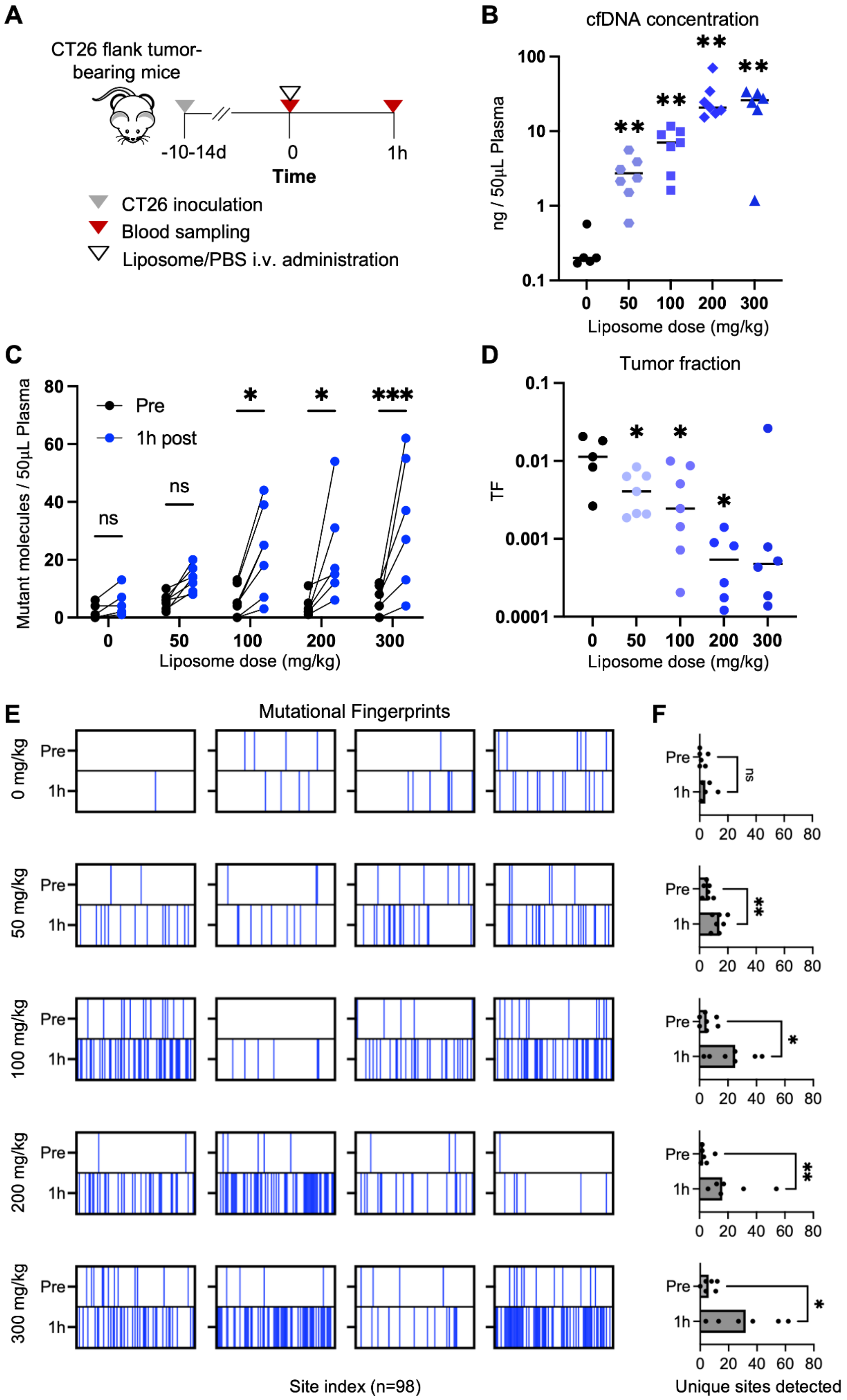
A
CT26 flank tumor-bearing mice
-10-14d
0
1h
Time
CT26 inoculation
Blood sampling
Liposome/PBS i.v. administration
B
cfDNA concentration
ng / 50μL Plasma
Liposome dose (mg/kg)
C
Mutant molecules / 50μL Plasma
Pre
1h post
ns
Liposome dose (mg/kg)
D
Tumor fraction
TF
Liposome dose (mg/kg)
E
Mutational Fingerprints
0 mg/kg
50 mg/kg
100 mg/kg
200 mg/kg
300 mg/kg
Pre
1h
Site index (n=98)
F
Unique sites detected

Fig. S18. SPE liposome priming increases the number of SNVs detected in a flank tumor model. (A) Experimental timeline to detect mutations in the plasma of CT26 tumor-bearing mice using the nanoparticle priming agent. Blood was drawn prior to and 1 hour after i.v. administration of PBS or liposomes at a range of doses (0-300 mg/kg). **(B)** cfDNA plasma concentration 1 hour after PBS or liposome treatment as quantified via qPCR **(C)** Number of mutant molecules (MT) per 50 μl of plasma pre- (black) and 1 h post- (blue) administration of liposomes and **(D)** median tumor fractions detected 1 hour post liposome-administration for each treatment group as quantified using the diagnostic ctDNA assay. Center line; median. **(E)** Mutational fingerprints showing individual sites (SNVs) detected for 4 representative mice per group. The top row represents SNVs detected pre-treatment, and the bottom row represents SNVs detected 1 hour post-treatment in a given plasma sample. Each vertical band corresponds to a different site in our 98-site SNV panel and is colored blue if detected at least once in the plasma sample. **(F)** Quantification of unique sites (SNVs) detected in (E). Bars; median. All panels refer to an experiment with *n* = 5-7 mice per group. ns $P > 0.05$, * $P < 0.05$, ** $P < 0.01$, two-tailed Mann-Whitney test.

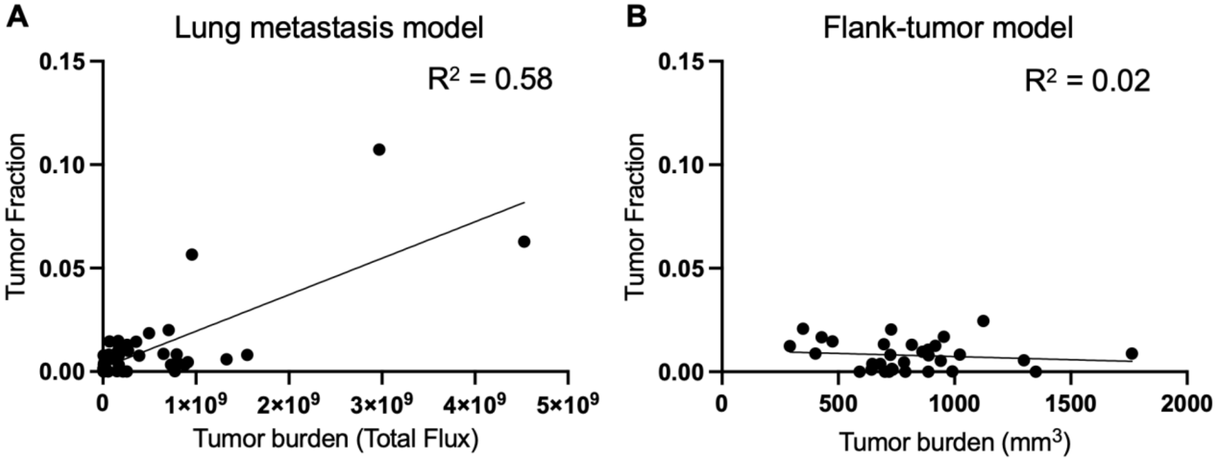

Fig. S19. Scatterplot of pre-treatment tumor fraction versus tumor burden. The scatterplot shows higher correlation **(A)** in a transplantation model of lung metastasis of the MC26 cell line vs. **(B)** in a CT26 bi-flank tumor model. This suggests earlier and more consistent access to the vasculature in the MC26 as well as better correlation between tumor burden and tumor fractions in plasma, as is typical for human tumors.

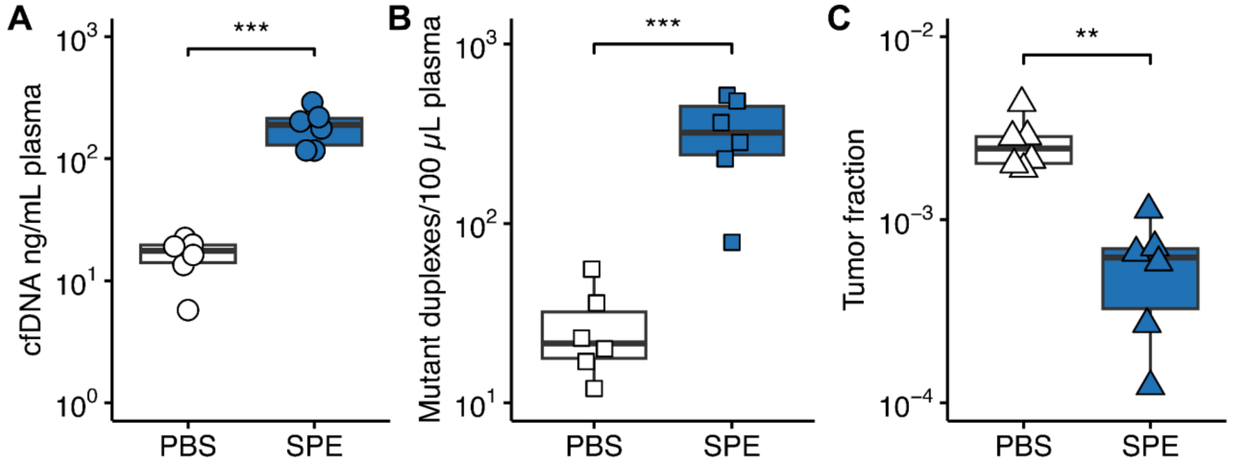

Fig. S20. Liposome priming improves ctDNA recovery in a murine lung metastasis model in an independent cohort. (A) Plasma cfDNA concentrations, **(B)** concentration of mutant molecules detected, and **(C)** tumor fractions detected 1 hour after administration of PBS or SPE liposomes (100 mg/kg) in an independent cohort of mice with Luc-MC26 lung metastases at 2 weeks post-inoculation (*n* = 6 mice per group). Boxplots in A-C show median and interquartile range; ns = not significant, * $P<0.05$, ** $P<0.01$, *** $P<0.001$; one-way ANOVA.

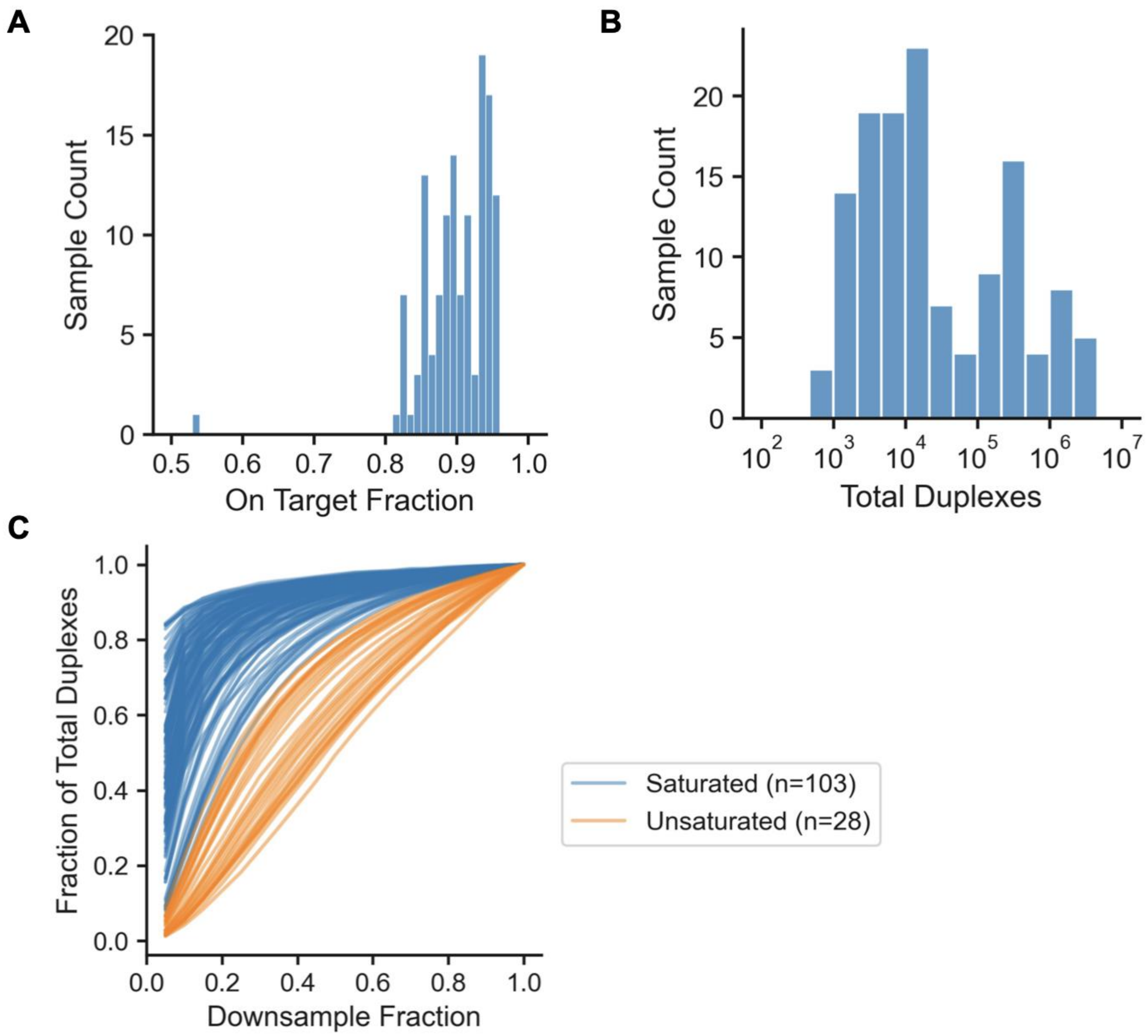

Fig. S21. Technical performance of the ctDNA diagnostic test tracking 1822 SNVs of the Luc-MC26 lung metastasis model. Histograms of **(A)** the on-target rate and **(B)** the total number of duplexes recovered. **(C)** Fraction of total duplexes after downsampling raw reads. We considered a sample to be saturated if it still contained at least 95% of its duplexes after downsampling to 80% of its raw reads.

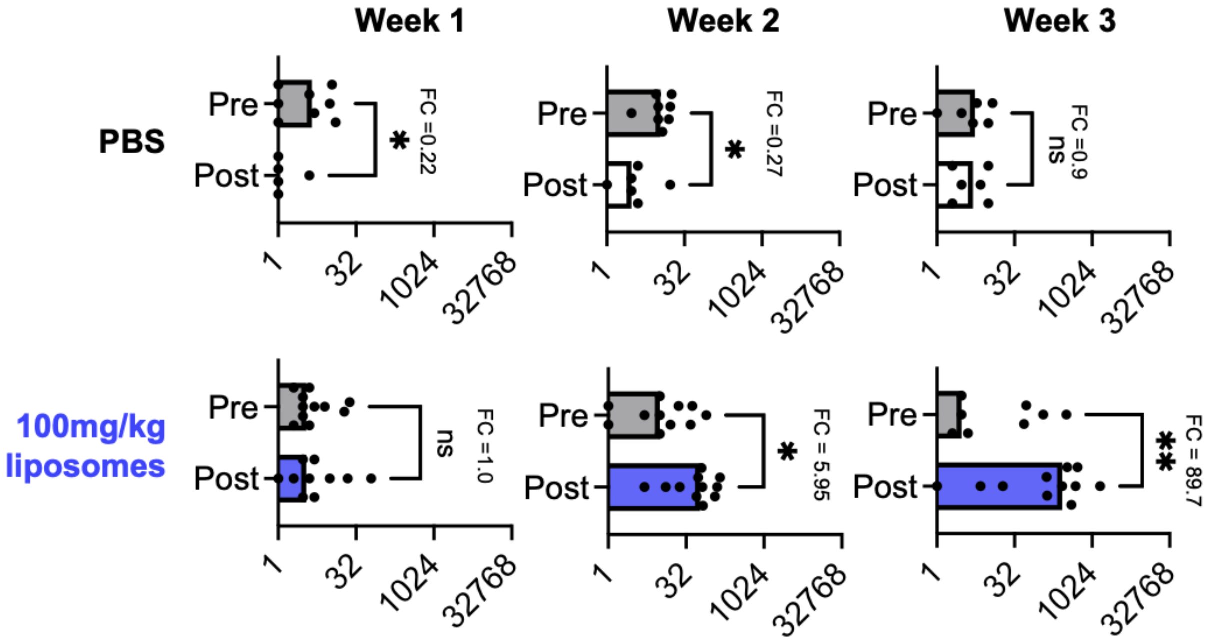

Fig. S22. Number of unique SNVs detected in Luc-MC26 tumor-bearing mice increases with liposome treatment. Blood was drawn prior to i.v. administration of PBS or liposomes (100 mg/kg) and 1 hour after treatment 1 week, 2 weeks, and 3 weeks after tumor inoculation. The liposome group had n=11-12 mice and the PBS group had n=6-8 mice. Fold changes (FC) in the number of unique SNVs detected are shown (1822 total SNVs). Liposome treatment resulted in significant increases in the number of SNVs detected post treatment at weeks 2 (5.95-fold, P<0.05) and week 3 (89.7-fold, P<0.01), whereas PBS treatment did not increase the number of SNVs detected. ns P > 0.05, * P < 0.05, ** P <0.01, two-tailed Mann-Whitney test.

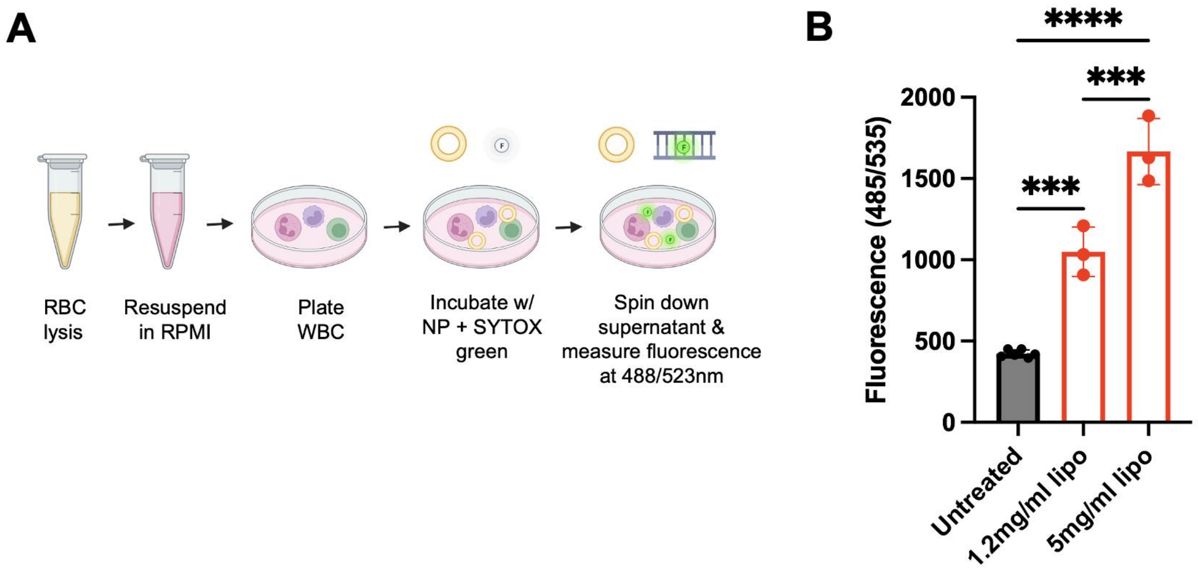

Fig. S23. Incubating primary murine white blood cells with liposomes in vitro led to increased detection of DNA in conditioned medium, as measured using SYTOX-green dye. **(A)** In vitro assay to assess cfDNA release from primary white blood cells harvested from mouse blood. **(B)** cfDNA content in conditioned medium following 2h treatment with different liposome doses or PBS, as quantified by SYTOX-DNA dye emission. One-way ANOVA, *** P<0.001, **** P<0.0001.

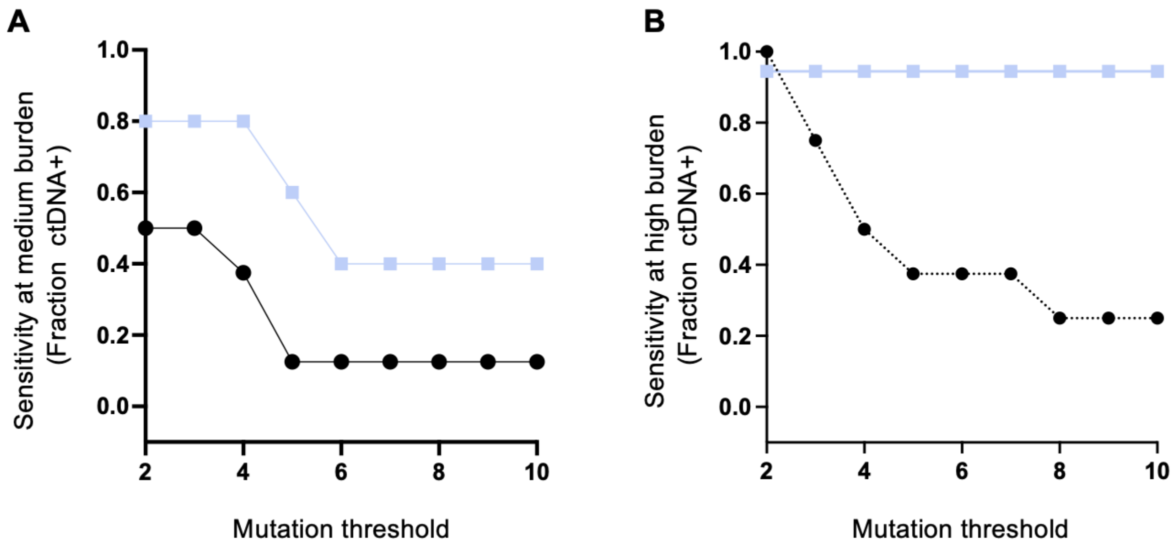

Fig. S24. Sensitivity of ctDNA tests. Sensitivity vs. mutation (SNV) threshold for tumor detection for **(A)** medium (total flux 1.5e7 p/s <burden < total flux 1.5e8 p/s) and **(B)** high (burden > total flux 1.5e8 p/s) tumor burden groups after 100 mg/kg liposome or PBS treatment. Sensitivity was calculated as the fraction of samples for which the number of mutations (SNVs) detected in a blood sample after treatment was equal or exceeded a given mutation (SNV) threshold.

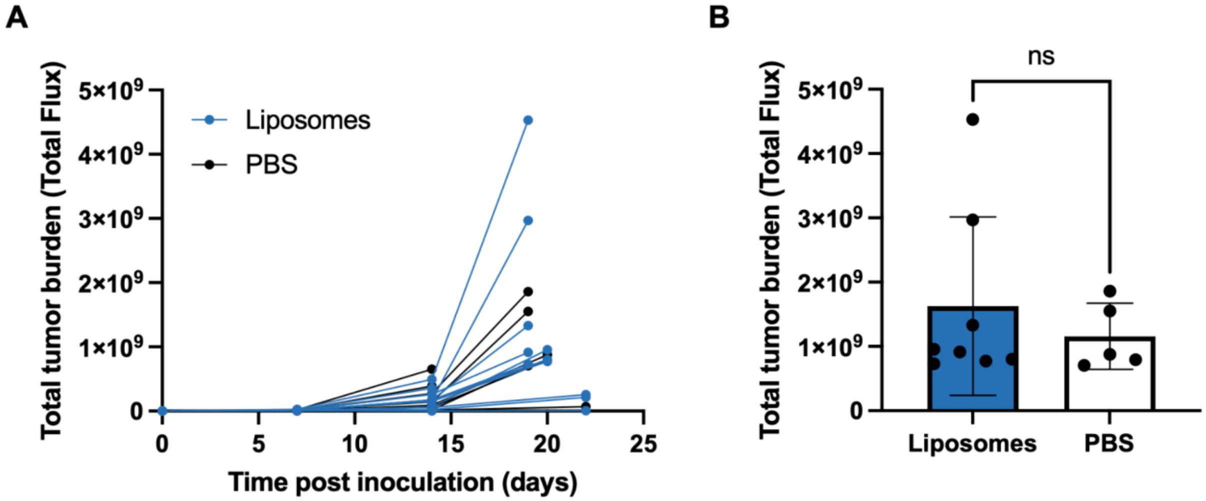

Fig. S25. In vivo luminescence imaging of tumor burden over time shows no difference in tumor growth between liposome and PBS-treated mice. Luc-MC26 tumors were inoculated via tail vein and IVIS imaging performed once a week during tumor progression. Mice were treated 3 times with liposomes (100 mg/kg) or PBS; on days 6, 14, and between days 19 and 21. **(A)** Total tumor burden over time and **(B)** tumor burden at days 19 and 20 post-tumor inoculation for each treatment group. ns P > 0.05, two-tailed unpaired t-test.

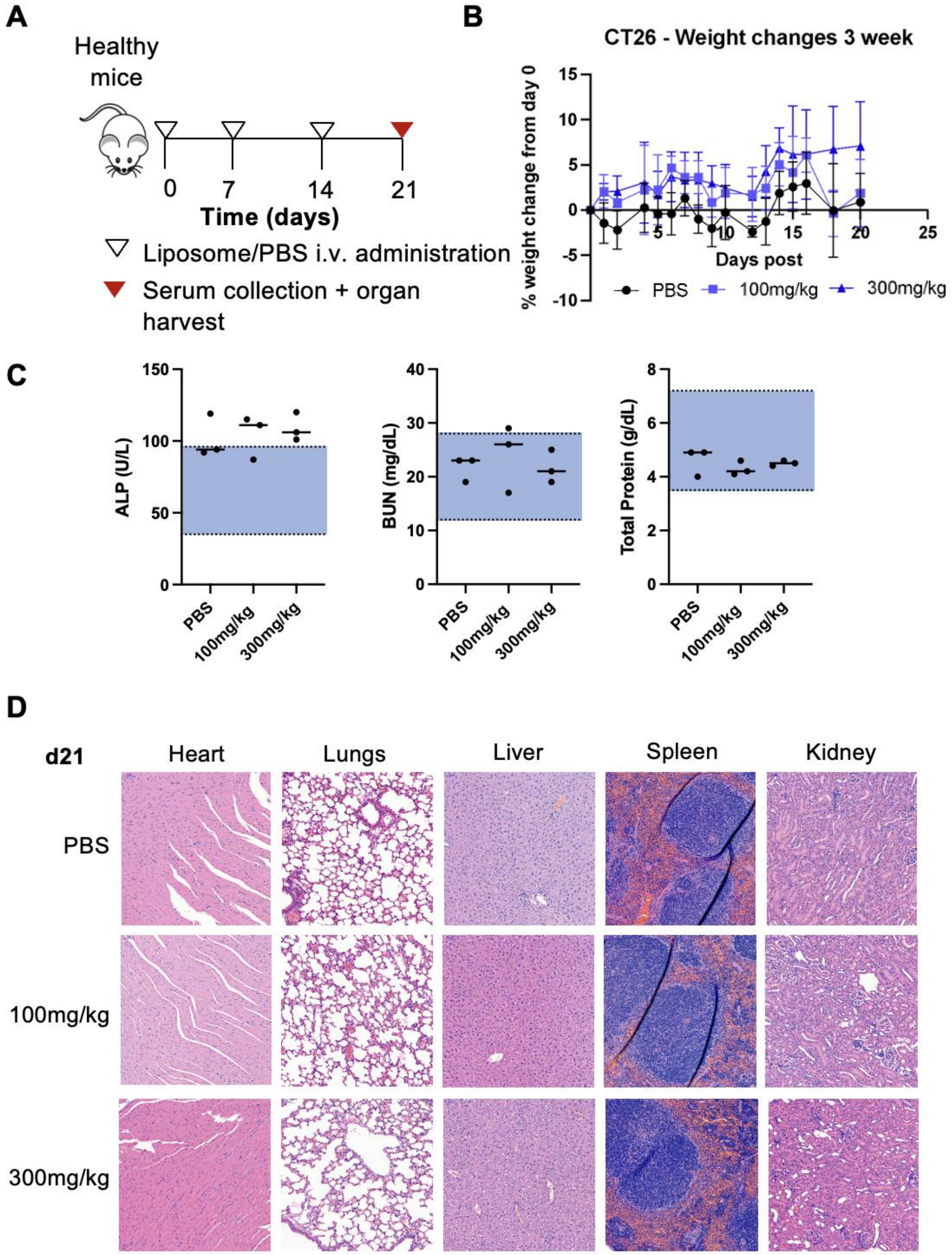

Fig. S26. Liposomal priming agent incurs no signs of acute toxicity or weight loss after 3 doses. (A) Experimental timeline to assess the toxicity of the nanoparticle priming agent in mice. **(B)** SPE-liposomes at either dose incurred no significant weight loss over the treatment course. **(C)** Serum chemistry assessing a basic liver and kidney panel showed no significant difference between PBS- and liposome-treated mice. **(D)** 7 days after the third liposome dose, organs (heart, lung, liver, spleen, and kidney) were collected, fixed, embedded in paraffin, and stained with

hematoxylin & eosin. Analysis by a veterinary pathologist confirmed that tissues from liposome-injected mice appeared similar to PBS injected controls, exhibiting no signs of toxicity. Study was done with n = 3 mice per group and images from representative animals are shown.

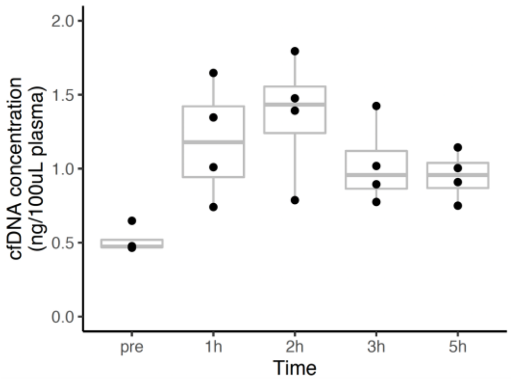

Fig. S27. cfDNA concentration levels after injection of 20 µg of aST3 antibody priming agent in healthy mice. aST3 (40 µg) was administered into healthy mice, and cfDNA levels were measured in blood collected longitudinally (n=4).

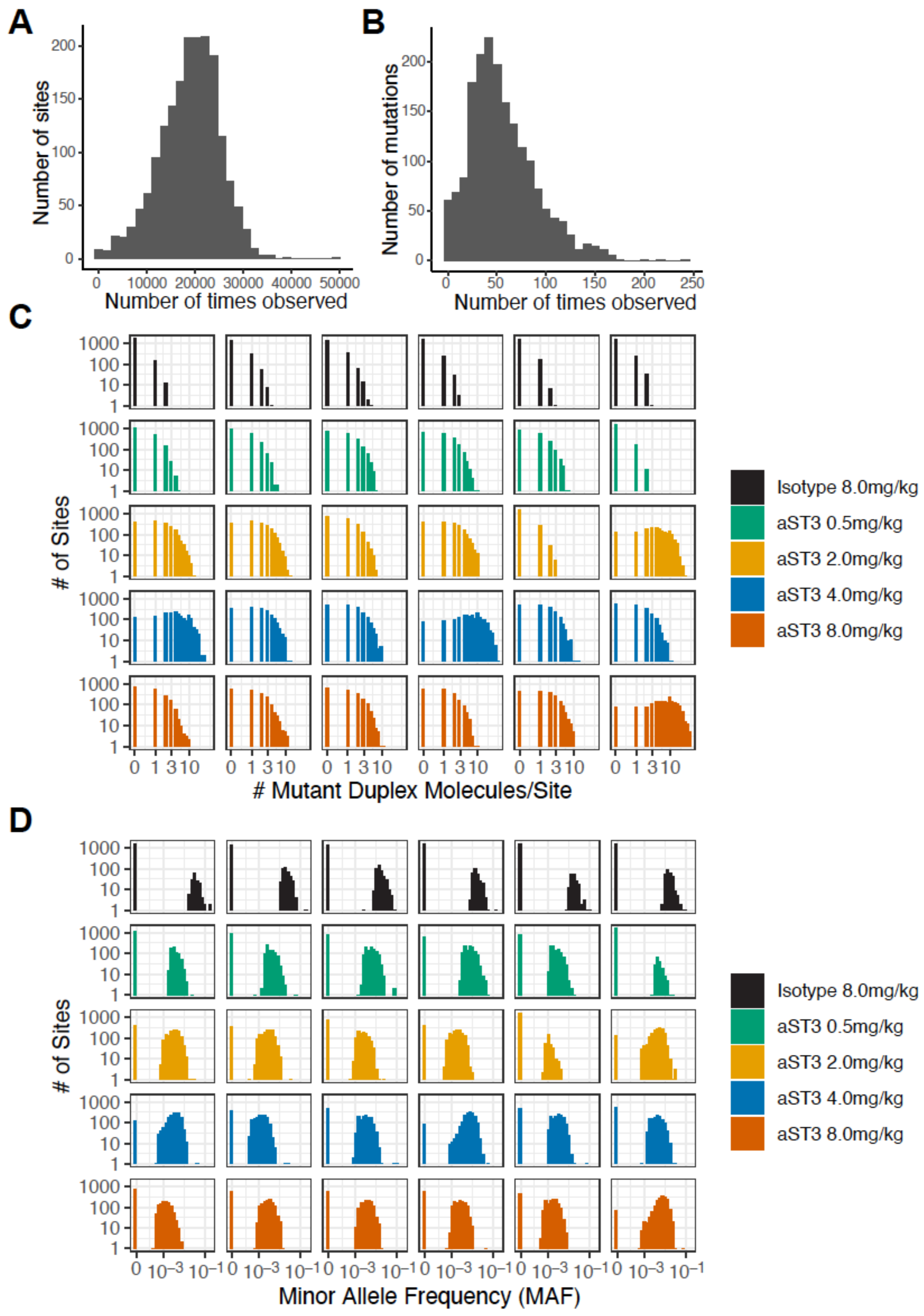

Fig. S28. Performance of 1822-SNV panel with aST3. Distribution of the number of times a **(A)** given SNV locus or **(B)** SNV was observed in the mouse cohort in Figure 5, demonstrating unimodal distributions with expected sampling variation, and without evidence of separate peaks suggestive of sites that are either preferentially lost or detected. **(C)** Distribution of number of mutant duplexes detected per site. **(D)** Mutant allele fraction (MAF) distribution across 1822 SNV loci. For C and D, each panel is data from an individual mouse. Colors indicate dose of aST3 or IgG2a isotype.

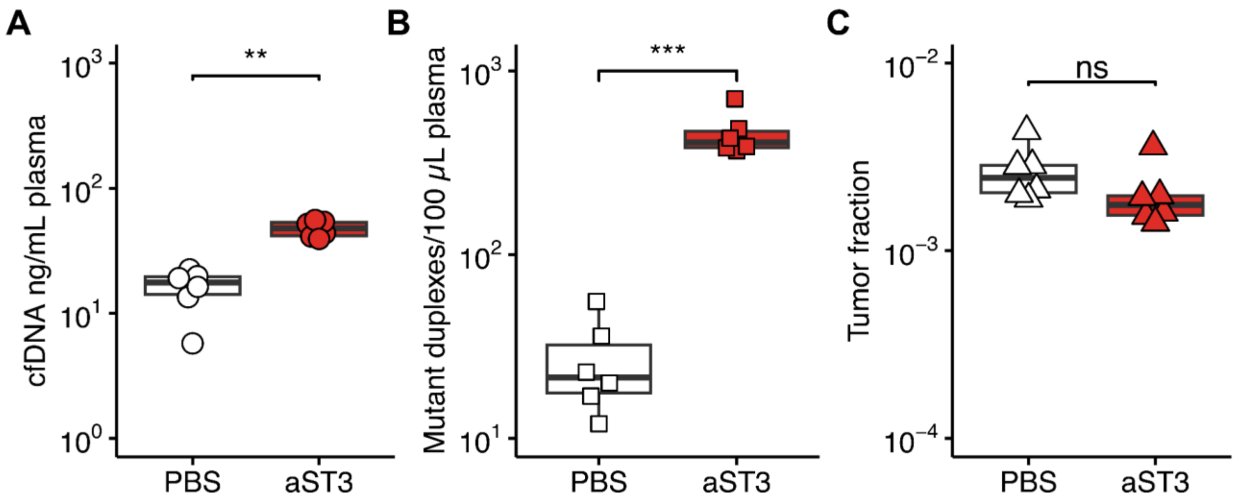

Fig. S29. Antibody priming agent improves ctDNA recovery in murine lung metastasis model in an independent cohort. (A) Plasma cfDNA concentrations, (**B**) concentration of mutant molecules detected, and (**C**) tumor fractions detected 2 hours after administration of PBS or aST3 at 4.0 mg/kg, in an independent cohort of mice with Luc-MC26 lung metastases (*n* = 6 mice per group). Boxplots in A-C show median and interquartile range; ns = not significant, * P<0.05, ** P<0.01, *** P<0.001; one-way ANOVA.

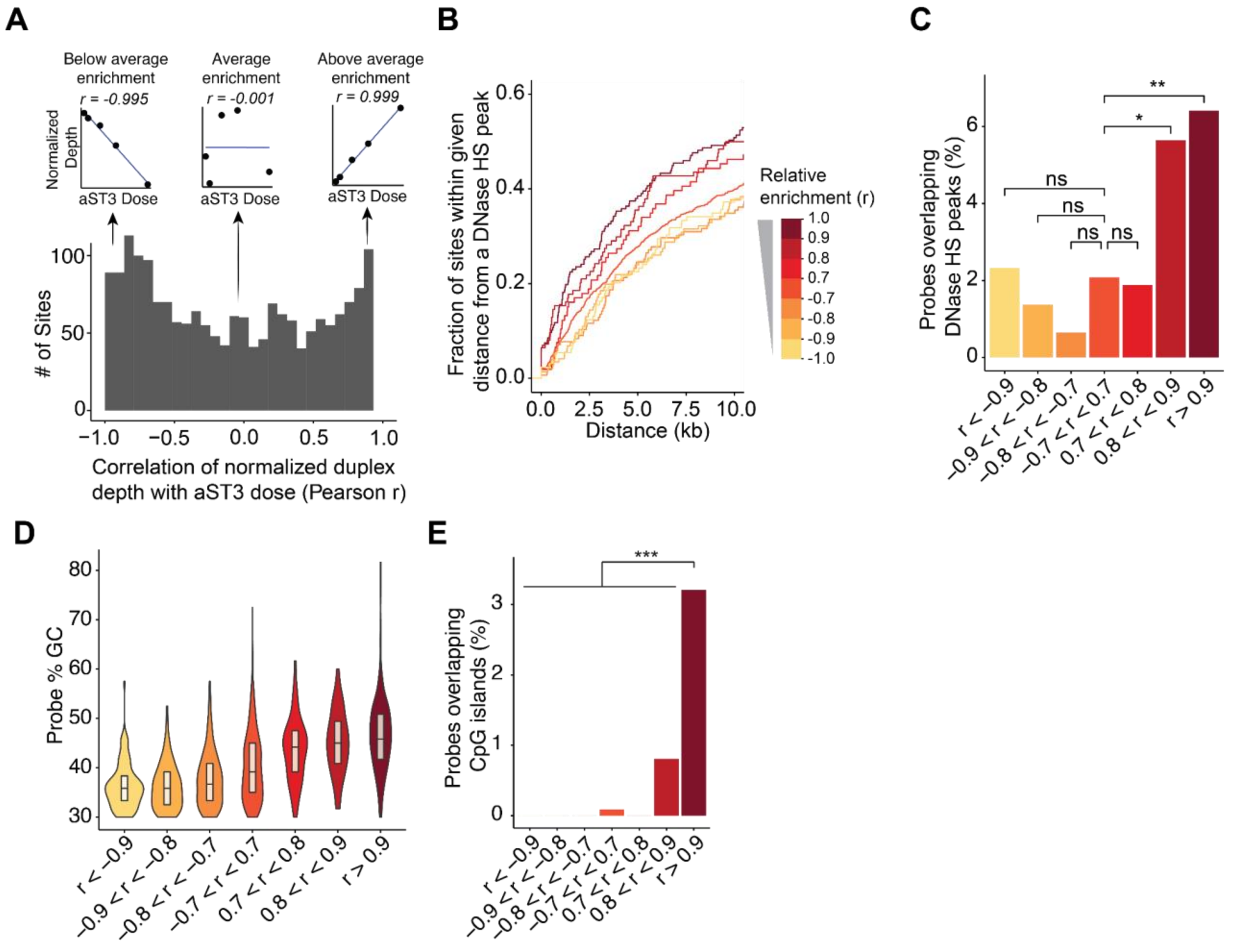

Fig. S30. DNase hypersensitivity and GC content are associated with higher relative enrichment from aST3. (A) Distribution of Pearson r correlation coefficients between aST3 dose and normalized duplex depth for 1,822 SNV loci. Inserts show scatterplots of normalized depth versus aST3 dose for three sample SNV loci exhibiting less than average, average or more than average enrichment (from left to right). **(B)** Proximity of 1,822 tracked SNV loci to leukocyte/myeloid DNase hypersensitivity peaks in mouse ENCODE, grouped by relative enrichment with higher aST3 doses. **(C)** Fraction of 1,822 probes overlapping leukocyte/myeloid DNase hypersensitivity peaks in mouse ENCODE within each relative enrichment group. 49 probes of 1822 total overlapped with DNase hypersensitive sites, and 164 probes overlapped or were within 1kb of DNase hypersensitive sites. **(D)** Correlation of GC content of 1,822 probes with the relative enrichment/depletion of the targeted SNV loci with aST3 treatment. Boxplots represent median and interquartile range. **(E)** Fraction of 1,822 probes overlapping CpG islands within each relative enrichment group. All panels refer to an experiment with n=6 mice per group. ns = not significant, * P<0.05, ** P<0.01, *** P<0.001; Fisher’s exact test.

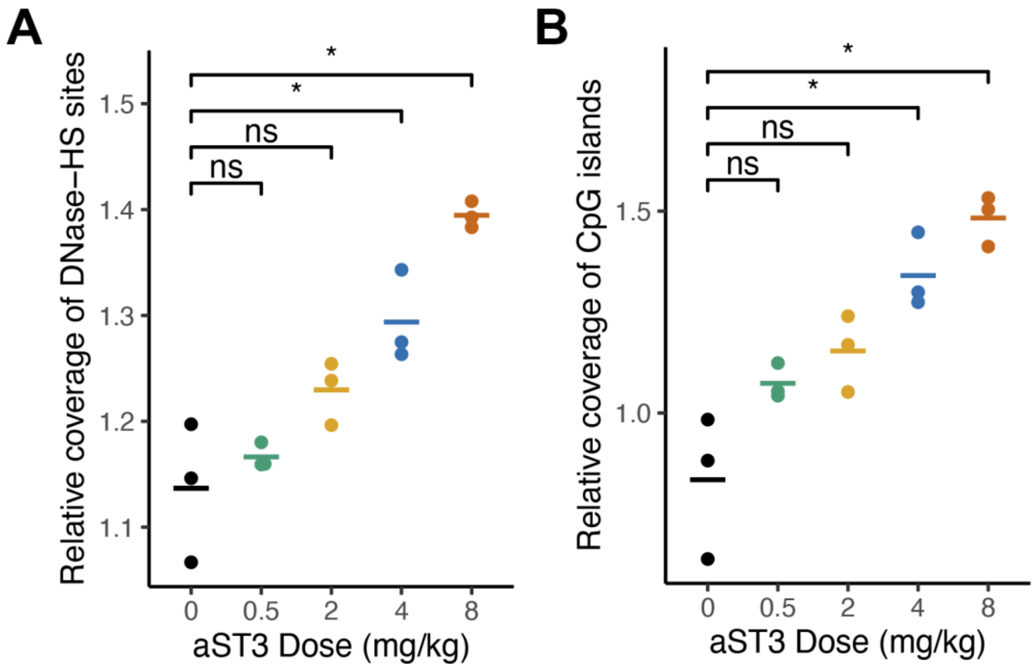

Fig. S31. Genome-wide coverage of DNase Hypersensitive (HS) sites and CpG islands 2 hours after aST3 administration. Relative coverage of **(A)** DNase-hypersensitive (DNase-HS) sites and **(B)** CpG islands with increasing doses of aST3 from whole-genome sequencing of plasma cell-free DNA. Relative coverage was calculated as mean coverage within sites of interest divided by mean coverage genome-wide.

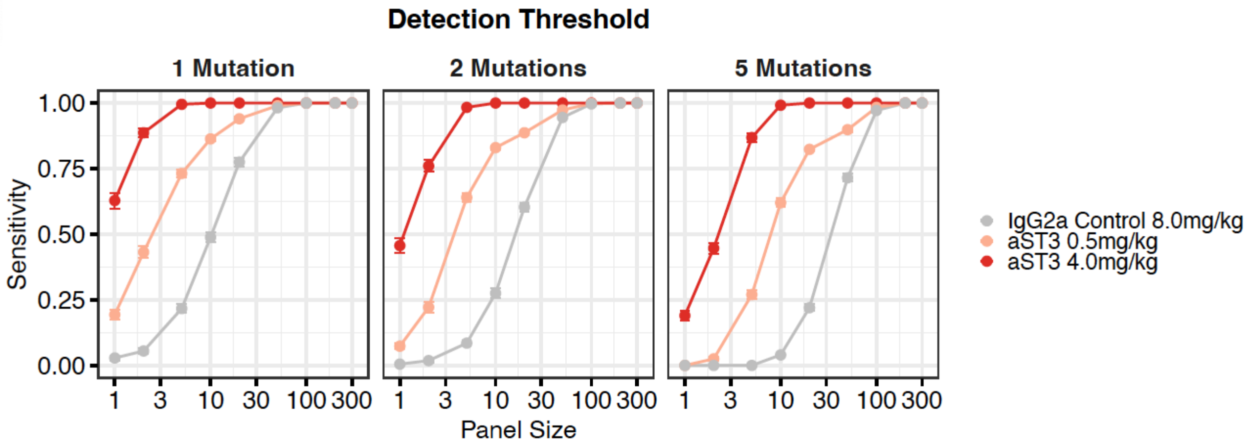

Fig. S32. Sensitivity for detection of ctDNA in plasma with or without antibody priming agent under various panel sizes and detection thresholds based on downsampling from full panel. Each point is mean +/- s.e.m. of 100 replicates.

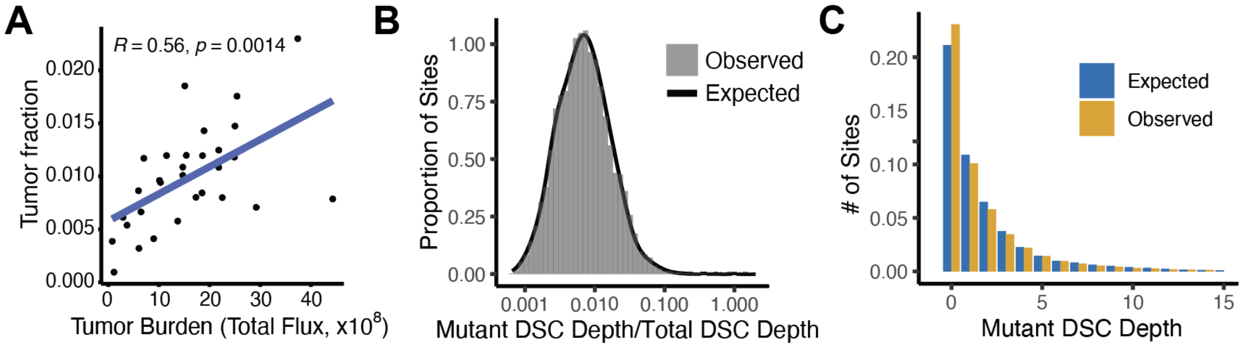

Fig. S33. A binomial model recapitulates the distribution of mutant molecules. (A) Correlation between lung tumor burden and concentration of mutant molecules per unit volume of plasma. **(B)** Comparison of resampled mutant duplex sequence consensus (DSC) depth/total DSC depth using a binomial model and the per-sample global tumor fraction (black density line) versus the observed distribution (bars). **(C)** Observed distribution of mutant DSC depth versus expected distribution using a binomial model.

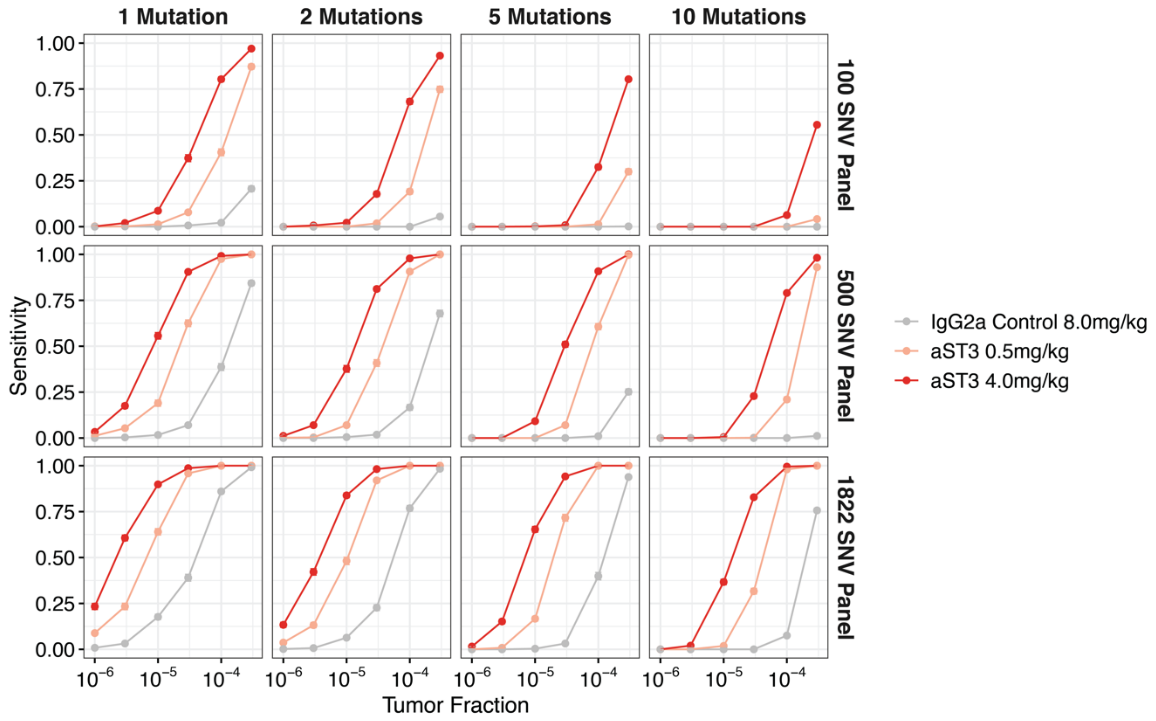

Fig. S34. Sensitivity for ctDNA detection with various panel sizes and detection thresholds. Estimated sensitivity at various detection thresholds [1-10 mutations (SNVs)] and panel sizes (100-1822 SNV panels) with and without priming. Points and error-bars represent mean and s.e.m. from 100 replicates.

Data S1. (separate file)
List of 98 tracked SNVs in CT26 panel, each carrying a SNV specific to the CT26 cell-line.

Data S2. (separate file)
List of 1,822 tracked SNVs in Luc-MC26 panel, each carrying a SNV specific to the Luc-MC26 cell-line.

Data S3. (separate file)
ctDNA test results and sample metadata for experiments in Fig. 4.

Data S4. (separate file)
ctDNA test results and sample metadata for experiments in Fig. S20 and Fig. S29.

Data S5. (separate file)
ctDNA test results and sample metadata for experiments in Fig. 5.

REFERENCES AND NOTES

1. Y. M. D. Lo, D. S. C. Han, P. Jiang, R. W. K. Chiu, Epigenetics, fragmentomics, and topology of cell-free DNA in liquid biopsies. *Science* **372**, eaaw3616 (2021). doi:10.1126/science.aaw3616 Medline

2. N. C. Rose, A. J. Kaimal, L. Dugoff, M. E. Norton; American College of Obstetricians and Gynecologists' Committee on Practice Bulletins—Obstetrics; Committee on Genetics; Society for Maternal-Fetal Medicine, Screening for Fetal Chromosomal Abnormalities: ACOG Practice Bulletin, Number 226. *Obstet. Gynecol.* **136**, e48–e69 (2020). doi:10.1097/AOG.0000000000004084 Medline

3. T. A. Blauwkamp, S. Thair, M. J. Rosen, L. Blair, M. S. Lindner, I. D. Vilfan, T. Kawli, F. C. Christians, S. Venkatasubrahmanyam, G. D. Wall, A. Cheung, Z. N. Rogers, G. Meshulam-Simon, L. Huijse, S. Balakrishnan, J. V. Quinn, D. Hollemon, D. K. Hong, M. L. Vaughn, M. Kertesz, S. Bercovici, J. C. Wilber, S. Yang, Analytical and clinical validation of a microbial cell-free DNA sequencing test for infectious disease. *Nat. Microbiol.* **4**, 663–674 (2019). doi:10.1038/s41564-018-0349-6 Medline

4. E. Heitzer, I. S. Haque, C. E. S. Roberts, M. R. Speicher, Current and future perspectives of liquid biopsies in genomics-driven oncology. *Nat. Rev. Genet.* **20**, 71–88 (2019). doi:10.1038/s41576-018-0071-5 Medline

5. I. De Vlaminck, H. A. Valantine, T. M. Snyder, C. Strehl, G. Cohen, H. Luikart, N. F. Neff, J. Okamoto, D. Bernstein, D. Weisshaar, S. R. Quake, K. K. Khush, Circulating Cell-Free DNA Enables Noninvasive Diagnosis of Heart Transplant Rejection. *Sci. Transl. Med.* **6**, 241ra77 (2014).

6. M. C. Liu, G. R. Oxnard, E. A. Klein, C. Swanton, M. V. Seiden, CCGA Consortium, Sensitive and specific multi-cancer detection and localization using methylation signatures in cell-free DNA. *Ann. Oncol.* **31**, 745–759 (2020). doi:10.1016/j.annonc.2020.02.011 Medline

7. A. Jamshidi, M. C. Liu, E. A. Klein, O. Venn, E. Hubbell, J. F. Beausang, S. Gross, C. Melton, A. P. Fields, Q. Liu, N. Zhang, E. T. Fung, K. N. Kurtzman, H. Amini, C. Betts, D. Civello, P. Freese, R. Calef, K. Davydov, S. Fayzullina, C. Hou, R. Jiang, B. Jung, S. Tang, V. Demas, J. Newman, O. Sakarya, E. Scott, A. Shenoy, S. Shojaee, K. K. Steffen, V. Nicula, T. C. Chien, S. Bagaria, N. Hunkapiller, M. Desai, Z. Dong, D. A. Richards, T. J. Yeatman, A. L. Cohn, D. D. Thiel, D. A. Berry, M. K. Tummala, K. McIntyre, M. A. Sekeres, A. Bryce, A. M. Aravanis, M. V. Seiden, C. Swanton, Evaluation of cell-free DNA approaches for multi-cancer early detection. *Cancer Cell* **40**, 1537–1549.e12 (2022). doi:10.1016/j.ccell.2022.10.022 Medline

8. M. G. O. Fernandes, N. Cruz-Martins, J. C. Machado, J. L. Costa, V. Hespanhol, The value of cell-free circulating tumour DNA profiling in advanced non-small cell lung cancer (NSCLC) management. *Cancer Cell Int.* **21**, 675 (2021). doi:10.1186/s12935-021-02382-0 Medline

9. H. A. Parsons, J. Rhoades, S. C. Reed, G. Gydush, P. Ram, P. Exman, K. Xiong, C. C. Lo, T. Li, M. Fleharty, G. J. Kirkner, D. Rotem, O. Cohen, F. Yu, M. Fitarelli-Kiehl, K. W. Leong, M. E. Hughes, S. M. Rosenberg, L. C. Collins, K. D. Miller, B. Blumenstiel, L. Trippa, C. Cibulskis, D. S. Neuberg, M. DeFelice, S. S. Freeman, N. J. Lennon, N.

Wagle, G. Ha, D. G. Stover, A. D. Choudhury, G. Getz, E. P. Winer, M. Meyerson, N. U. Lin, I. Krop, J. C. Love, G. M. Makrigiorgos, A. H. Partridge, E. L. Mayer, T. R. Golub, V. A. Adalsteinsson, Sensitive Detection of Minimal Residual Disease in Patients Treated for Early-Stage Breast Cancer. *Clin. Cancer Res.* **26**, 2556–2564 (2020). doi:10.1158/1078-0432.CCR-19-3005 Medline

10. A. R. Parikh, E. E. Van Seventer, G. Siravegna, A. V. Hartwig, A. Jaimovich, Y. He, K. Kanter, M. G. Fish, K. D. Fosbenner, B. Miao, S. Phillips, J. H. Carmichael, N. Sharma, J. Jarnagin, I. Baiev, Y. S. Shah, I. J. Fetter, H. A. Shahzade, J. N. Allen, L. S. Blaszkowsky, J. W. Clark, J. S. Dubois, J. W. Franses, B. J. Giantonio, L. Goyal, S. J. Klempner, R. D. Nipp, E. J. Roeland, D. P. Ryan, C. D. Weekes, J. Y. Wo, T. S. Hong, L. Bordeianou, C. R. Ferrone, M. Qadan, H. Kunitake, D. Berger, R. Ricciardi, J. C. Cusack, V. M. Raymond, A. Talasaz, G. M. Boland, R. B. Corcoran, Minimal Residual Disease Detection using a Plasma-only Circulating Tumor DNA Assay in Patients with Colorectal Cancer. *Clin. Cancer Res.* **27**, 5586–5594 (2021). doi:10.1158/1078-0432.CCR-21-0410 Medline

11. E. J. Moding, B. Y. Nabet, A. A. Alizadeh, M. Diehn, Detecting Liquid Remnants of Solid Tumors: Circulating Tumor DNA Minimal Residual Disease. *Cancer Discov.* **11**, 2968–2986 (2021). doi:10.1158/2159-8290.CD-21-0634 Medline

12. A. M. Newman, A. F. Lovejoy, D. M. Klass, D. M. Kurtz, J. J. Chabon, F. Scherer, H. Stehr, C. L. Liu, S. V. Bratman, C. Say, L. Zhou, J. N. Carter, R. B. West, G. W. Sledge Jr., J. B. Shrager, B. W. Loo Jr., J. W. Neal, H. A. Wakelee, M. Diehn, A. A. Alizadeh, Integrated digital error suppression for improved detection of circulating tumor DNA. *Nat. Biotechnol.* **34**, 547–555 (2016). doi:10.1038/nbt.3520 Medline

13. M. W. Schmitt, S. R. Kennedy, J. J. Salk, E. J. Fox, J. B. Hiatt, L. A. Loeb, Detection of ultra-rare mutations by next-generation sequencing. *Proc. Natl. Acad. Sci. U.S.A.* **109**, 14508–14513 (2012). doi:10.1073/pnas.1208715109 Medline

14. D. M. Kurtz, J. Soo, L. Co Ting Keh, S. Alig, J. J. Chabon, B. J. Sworder, A. Schultz, M. C. Jin, F. Scherer, A. Garofalo, C. W. Macaulay, E. G. Hamilton, B. Chen, M. Olsen, J. G. Schroers-Martin, A. F. M. Craig, E. J. Moding, M. S. Esfahani, C. L. Liu, U. Dührsen, A. Hüttmann, R.-O. Casasnovas, J. R. Westin, M. Roschewski, W. H. Wilson, G. Gaidano, D. Rossi, M. Diehn, A. A. Alizadeh, Enhanced detection of minimal residual disease by targeted sequencing of phased variants in circulating tumor DNA. *Nat. Biotechnol.* **39**, 1537–1547 (2021). doi:10.1038/s41587-021-00981-w Medline

15. G. Gydush, E. Nguyen, J. H. Bae, T. Blewett, J. Rhoades, S. C. Reed, D. Shea, K. Xiong, R. Liu, F. Yu, K. W. Leong, A. D. Choudhury, D. G. Stover, S. M. Tolaney, I. E. Krop, J. Christopher Love, H. A. Parsons, G. Mike Makrigiorgos, T. R. Golub, V. A. Adalsteinsson, Massively parallel enrichment of low-frequency alleles enables duplex sequencing at low depth. *Nat. Biomed. Eng.* **6**, 257–266 (2022). doi:10.1038/s41551-022-00855-9 Medline

16. A. Zviran, R. C. Schulman, M. Shah, S. T. K. Hill, S. Deochand, C. C. Khamnei, D. Maloney, K. Patel, W. Liao, A. J. Widman, P. Wong, M. K. Callahan, G. Ha, S. Reed, D. Rotem, D. Frederick, T. Sharova, B. Miao, T. Kim, G. Gydush, J. Rhoades, K. Y. Huang, N. D. Omans, P. O. Bolan, A. H. Lipsky, C. Ang, M. Malbari, C. F. Spinelli, S. Kazancioglu, A. M. Runnels, S. Fennessey, C. Stolte, F. Gaiti, G. G. Inghirami, V.

Adalsteinsson, B. Houck-Loomis, J. Ishii, J. D. Wolchok, G. Boland, N. Robine, N. K. Altorki, D. A. Landau, Genome-wide cell-free DNA mutational integration enables ultra-sensitive cancer monitoring. *Nat. Med.* **26**, 1114–1124 (2020). doi:10.1038/s41591-020-0915-3 Medline

17. L. Keller, Y. Belloum, H. Wikman, K. Pantel, Clinical relevance of blood-based ctDNA analysis: Mutation detection and beyond. *Br. J. Cancer* **124**, 345–358 (2021). doi:10.1038/s41416-020-01047-5 Medline

18. F. Chemi, S. P. Pearce, A. Clipson, S. M. Hill, A.-M. Conway, S. A. Richardson, K. Kamieniecka, R. Caeser, D. J. White, S. Mohan, V. Foy, K. L. Simpson, M. Galvin, K. K. Frese, L. Priest, J. Egger, A. Kerr, P. P. Massion, J. T. Poirier, G. Brady, F. Blackhall, D. G. Rothwell, C. M. Rudin, C. Dive, cfDNA methylome profiling for detection and subtyping of small cell lung cancers. *Nat. Cancer* **3**, 1260–1270 (2022). doi:10.1038/s43018-022-00415-9 Medline

19. S. Cristiano, A. Leal, J. Phallen, J. Fiksel, V. Adleff, D. C. Bruhm, S. Ø. Jensen, J. E. Medina, C. Hruban, J. R. White, D. N. Palsgrove, N. Niknafs, V. Anagnostou, P. Forde, J. Naidoo, K. Marrone, J. Brahmer, B. D. Woodward, H. Husain, K. L. van Rooijen, M. W. Ørntoft, A. H. Madsen, C. J. H. van de Velde, M. Verheij, A. Cats, C. J. A. Punt, G. R. Vink, N. C. T. van Grieken, M. Koopman, R. J. A. Fijneman, J. S. Johansen, H. J. Nielsen, G. A. Meijer, C. L. Andersen, R. B. Scharpf, V. E. Velculescu, Genome-wide cell-free DNA fragmentation in patients with cancer. *Nature* **570**, 385–389 (2019). doi:10.1038/s41586-019-1272-6 Medline

20. C. Fiala, E. P. Diamandis, Can a Broad Molecular Screen Based on Circulating Tumor DNA Aid in Early Cancer Detection? *J. Appl. Lab. Med.* **5**, 1372–1377 (2020). doi:10.1093/jalm/jfaa138 Medline

21. M. Ignatiadis, G. W. Sledge, S. S. Jeffrey, Liquid biopsy enters the clinic - implementation issues and future challenges. *Nat. Rev. Clin. Oncol.* **18**, 297–312 (2021). doi:10.1038/s41571-020-00457-x Medline

22. A. Tivey, M. Church, D. Rothwell, C. Dive, N. Cook, Circulating tumour DNA - looking beyond the blood. *Nat. Rev. Clin. Oncol.* **19**, 600–612 (2022). doi:10.1038/s41571-022-00660-y Medline

23. L. Zhu, A. Nazeri, C. P. Pacia, Y. Yue, H. Chen, Focused ultrasound for safe and effective release of brain tumor biomarkers into the peripheral circulation. *PLOS ONE* **15**, e0234182 (2020). doi:10.1371/journal.pone.0234182 Medline

24. J. M. Noh, Y. J. Kim, H. Y. Lee, C. Choi, W.-G. Ahn, T. Lee, H. Pyo, J. H. Park, D. Park, W.-Y. Park, Targeted Liquid Biopsy Using Irradiation to Facilitate the Release of Cell-Free DNA from a Spatially Aimed Tumor Tissue. *Cancer Res. Treat.* **54**, 40–53 (2022). doi:10.4143/crt.2021.151 Medline

25. A. Kustanovich, R. Schwartz, T. Peretz, A. Grinshpun, Life and death of circulating cell-free DNA. *Cancer Biol. Ther.* **20**, 1057–1067 (2019). doi:10.1080/15384047.2019.1598759 Medline

26. S. Khier, P. B. Gahan, Hepatic Clearance of Cell-Free DNA: Possible Impact on Early Metastasis Diagnosis. *Mol. Diagn. Ther.* **25**, 677–682 (2021). doi:10.1007/s40291-021-00554-2 Medline

27. D. S. C. Han, Y. M. D. Lo, The Nexus of cfDNA and Nuclease Biology. *Trends Genet.* **37**, 758–770 (2021). doi:10.1016/j.tig.2021.04.005 Medline

28. M. Germain, M.-E. Meyre, L. Poul, M. Paolini, C. Berjaud, F. Mpambani, M. Bergere, L. Levy, A. Pottier, Priming the body to receive the therapeutic agent to redefine treatment benefit/risk profile. *Sci. Rep.* **8**, 4797 (2018). doi:10.1038/s41598-018-23140-9 Medline

29. T. Liu, H. Choi, R. Zhou, I.-W. Chen, RES blockade: A strategy for boosting efficiency of nanoparticle drug. *Nano Today* **10**, 11–21 (2015). doi:10.1016/j.nantod.2014.12.003

30. B. Ouyang, W. Poon, Y.-N. Zhang, Z. P. Lin, B. R. Kingston, A. J. Tavares, Y. Zhang, J. Chen, M. S. Valic, A. M. Syed, P. MacMillan, J. Couture-Senécal, G. Zheng, W. C. W. Chan, The dose threshold for nanoparticle tumour delivery. *Nat. Mater.* **19**, 1362–1371 (2020). doi:10.1038/s41563-020-0755-z Medline

31. N. R. M. Saunders, M. S. Paolini, O. S. Fenton, L. Poul, J. Devalliere, F. Mpambani, A. Darmon, M. Bergère, O. Jibault, M. Germain, R. Langer, A Nanoprimer To Improve the Systemic Delivery of siRNA and mRNA. *Nano Lett.* **20**, 4264–4269 (2020). doi:10.1021/acs.nanolett.0c00752 Medline

32. M. Jain, N. Kamal, S. K. Batra, Engineering antibodies for clinical applications. *Trends Biotechnol.* **25**, 307–316 (2007). doi:10.1016/j.tibtech.2007.05.001 Medline

33. R.-M. Lu, Y.-C. Hwang, I.-J. Liu, C.-C. Lee, H.-Z. Tsai, H.-J. Li, H.-C. Wu, Development of therapeutic antibodies for the treatment of diseases. *J. Biomed. Sci.* **27**, 1 (2020). doi:10.1186/s12929-019-0592-z Medline

34. P. Liu, G. Chen, J. Zhang, A Review of Liposomes as a Drug Delivery System: Current Status of Approved Products, Regulatory Environments, and Future Perspectives. *Molecules* **27**, 1372 (2022). doi:10.3390/molecules27041372 Medline

35. C. D. Walkey, J. B. Olsen, H. Guo, A. Emili, W. C. W. Chan, Nanoparticle size and surface chemistry determine serum protein adsorption and macrophage uptake. *J. Am. Chem. Soc.* **134**, 2139–2147 (2012). doi:10.1021/ja2084338 Medline

36. Y.-N. Zhang, W. Poon, A. J. Tavares, I. D. McGilvray, W. C. W. Chan, Nanoparticle-liver interactions: Cellular uptake and hepatobiliary elimination. *J. Control. Release* **240**, 332–348 (2016). doi:10.1016/j.jconrel.2016.01.020 Medline

37. C. Rosales, E. Uribe-Querol, Phagocytosis: A Fundamental Process in Immunity. *BioMed Res. Int.* **2017**, 9042851 (2017). doi:10.1155/2017/9042851 Medline

38. P. T. Lowary, J. Widom, New DNA sequence rules for high affinity binding to histone octamer and sequence-directed nucleosome positioning. *J. Mol. Biol.* **276**, 19–42 (1998). doi:10.1006/jmbi.1997.1494 Medline

39. N. H. H. Heegaard, D. T. Olsen, K.-L. P. Larsen, Immuno-capillary electrophoresis for the characterization of a monoclonal antibody against DNA. *J. Chromatogr. A* **744**, 285–294 (1996). doi:10.1016/0021-9673(96)00425-6 Medline

40. E. Ben Chetrit, E. H. Dunsky, S. Wollner, D. Eilat, In vivo clearance and tissue uptake of an anti-DNA monoclonal antibody and its complexes with DNA. *Clin. Exp. Immunol.* **60**, 159–168 (1985). Medline

41. I. Mellman, H. Plutner, Internalization and degradation of macrophage Fc receptors bound to polyvalent immune complexes. *J. Cell Biol.* **98**, 1170–1177 (1984). doi:10.1083/jcb.98.4.1170 Medline

42. F. Junker, J. Gordon, O. Qureshi, Fc Gamma Receptors and Their Role in Antigen Uptake, Presentation, and T Cell Activation. *Front. Immunol.* **11**, 1393 (2020). doi:10.3389/fimmu.2020.01393 Medline

43. R. Liu, R. J. Oldham, E. Teal, S. A. Beers, M. S. Cragg, Fc-Engineering for Modulated Effector Functions-Improving Antibodies for Cancer Treatment. *Antibodies (Basel)* **9**, 64 (2020). doi:10.3390/antib9040064 Medline

44. X. Wang, M. Mathieu, R. J. Brezski, IgG Fc engineering to modulate antibody effector functions. *Protein Cell* **9**, 63–73 (2018). doi:10.1007/s13238-017-0473-8 Medline

45. M. R. Walker, J. Lund, K. M. Thompson, R. Jefferis, Aglycosylation of human IgG1 and IgG3 monoclonal antibodies can eliminate recognition by human cells expressing Fc γ RI and/or Fc γ RII receptors. *Biochem. J.* **259**, 347–353 (1989). doi:10.1042/bj2590347 Medline

46. M. H. Tao, S. L. Morrison, Studies of aglycosylated chimeric mouse-human IgG. Role of carbohydrate in the structure and effector functions mediated by the human IgG constant region. *J. Immunol.* **143**, 2595–2601 (1989). doi:10.4049/jimmunol.143.8.2595 Medline

47. M. K. Leabman, Y. G. Meng, R. F. Kelley, L. E. DeForge, K. J. Cowan, S. Iyer, Effects of altered FcγR binding on antibody pharmacokinetics in cynomolgus monkeys. *MAbs* **5**, 896–903 (2013). doi:10.4161/mabs.26436 Medline

48. T. Schlothauer, S. Herter, C. F. Koller, S. Grau-Richards, V. Steinhart, C. Spick, M. Kubbies, C. Klein, P. Umaña, E. Mössner, Novel human IgG1 and IgG4 Fc-engineered antibodies with completely abolished immune effector functions. *Protein Eng. Des. Sel.* **29**, 457–466 (2016). doi:10.1093/protein/gzw040 Medline

49. R. A. Clynes, T. L. Towers, L. G. Presta, J. V. Ravetch, Inhibitory Fc receptors modulate in vivo cytotoxicity against tumor targets. *Nat. Med.* **6**, 443–446 (2000). doi:10.1038/74704 Medline

50. L. Baudino, Y. Shinohara, F. Nimmerjahn, J. Furukawa, M. Nakata, E. Martínez-Soria, F. Petry, J. V. Ravetch, S. Nishimura, S. Izui, Crucial role of aspartic acid at position 265 in the CH2 domain for murine IgG2a and IgG2b Fc-associated effector functions. *J. Immunol.* **181**, 6664–6669 (2008). doi:10.4049/jimmunol.181.9.6664 Medline

51. S. W. Hosea, E. J. Brown, M. I. Hamburger, M. M. Frank, Opsonic requirements for intravascular clearance after splenectomy. *N. Engl. J. Med.* **304**, 245–250 (1981). doi:10.1056/NEJM198101293040501 Medline

52. Materials and methods are available as supplementary materials.

53. A. N. Ilinskaya, M. A. Dobrovolskaia, Nanoparticles and the blood coagulation system. Part I: Benefits of nanotechnology. *Nanomedicine (Lond.)* **8**, 773–784 (2013). doi:10.2217/nnm.13.48 Medline

54. J. Tie, J. D. Cohen, K. Lahouel, S. N. Lo, Y. Wang, S. Kosmider, R. Wong, J. Shapiro, M. Lee, S. Harris, A. Khattak, M. Burge, M. Harris, J. Lynam, L. Nott, F. Day, T. Hayes, S.-A. McLachlan, B. Lee, J. Ptak, N. Silliman, L. Dobbyn, M. Popoli, R. Hruban, A. M.

Lennon, N. Papadopoulos, K. W. Kinzler, B. Vogelstein, C. Tomasetti, P. Gibbs; DYNAMIC Investigators, Circulating Tumor DNA Analysis Guiding Adjuvant Therapy in Stage II Colon Cancer. *N. Engl. J. Med.* **386**, 2261–2272 (2022). doi:10.1056/NEJMoa2200075 Medline

55. P. M. Kasi, F. Dayyani, V. K. Morris, S. Kopetz, A. R. Parikh, J. S. Starr, S. Cohen, A. Grothey, C. H. Lieu, M. H. O'Hara, K. Loranger, L. Westbrook, S. Sharma, S. Krinshpun, N. Hook, B. Zimmermann, P. R. Billings, A. Aleshin, Tumor-informed assessment of molecular residual disease and its incorporation into practice for patients with early and advanced-stage colorectal cancer (CRC-MRD Consortia). *J. Clin. Oncol.* **38**, 4108 (2020). doi:10.1200/JCO.2020.38.15_suppl.4108

56. S. Avanzini, D. M. Kurtz, J. J. Chabon, E. J. Moding, S. S. Hori, S. S. Gambhir, A. A. Alizadeh, M. Diehn, J. G. Reiter, A mathematical model of ctDNA shedding predicts tumor detection size. *Sci. Adv.* **6**, eabc4308 (2020). doi:10.1126/sciadv.abc4308 Medline

57. J. C. M. Wan, K. Heider, D. Gale, S. Murphy, E. Fisher, F. Mouliere, A. Ruiz-Valdepenas, A. Santonja, J. Morris, D. Chandrananda, A. Marshall, A. B. Gill, P. Y. Chan, E. Barker, G. Young, W. N. Cooper, I. Hudecova, F. Marass, R. Mair, K. M. Brindle, G. D. Stewart, J. E. Abraham, C. Caldas, D. M. Rassl, R. C. Rintoul, C. Alifrangis, M. R. Middleton, F. A. Gallagher, C. Parkinson, A. Durrani, U. McDermott, C. G. Smith, C. Massie, P. G. Corrie, N. Rosenfeld, ctDNA monitoring using patient-specific sequencing and integration of variant reads. *Sci. Transl. Med.* **12**, eaaz8084 (2020). doi:10.1126/scitranslmed.aaz8084 Medline

58. B. R. McDonald, T. Contente-Cuomo, S.-J. Sammut, A. Odenheimer-Bergman, B. Ernst, N. Perdigones, S.-F. Chin, M. Farooq, R. Mejia, P. A. Cronin, K. S. Anderson, H. E. Kosiorek, D. W. Northfelt, A. E. McCullough, B. K. Patel, J. N. Weitzel, T. P. Slavin, C. Caldas, B. A. Pockaj, M. Murtaza, Personalized circulating tumor DNA analysis to detect residual disease after neoadjuvant therapy in breast cancer. *Sci. Transl. Med.* **11**, eaax7392 (2019). doi:10.1126/scitranslmed.aax7392 Medline

59. V. S. Nair, A. B.-Y. Hui, J. J. Chabon, M. S. Esfahani, H. Stehr, B. Y. Nabet, L. Zhou, A. A. Chaudhuri, J. Benson, K. Ayers, H. Bedi, M. Ramsey, R. Van Wert, S. Antic, N. Lui, L. Backhus, M. Berry, A. W. Sung, P. P. Massion, J. B. Shrager, A. A. Alizadeh, M. Diehn, Genomic Profiling of Bronchoalveolar Lavage Fluid in Lung Cancer. *Cancer Res.* **82**, 2838–2847 (2022). doi:10.1158/0008-5472.CAN-22-0554 Medline

60. H. A. Parsons, T. Blewett, X. Chu, S. Sridhar, K. Santos, K. Xiong, V. G. Abramson, A. Patel, J. Cheng, A. Brufsky, J. Rhoades, J. Force, R. Liu, T. A. Traina, L. A. Carey, M. F. Rimawi, K. D. Miller, V. Stearns, J. Specht, C. Falkson, H. J. Burstein, A. C. Wolff, E. P. Winer, N. Tayob, I. E. Krop, G. M. Makrigiorgos, T. R. Golub, E. L. Mayer, V. A. Adalsteinsson, Circulating tumor DNA association with residual cancer burden after neoadjuvant chemotherapy in triple-negative breast cancer in TBCRC 030. *Ann. Oncol.* **34**, 899–906 (2023). doi:10.1016/j.annonc.2023.08.004 Medline

61. X. Hou, T. Zaks, R. Langer, Y. Dong, Lipid nanoparticles for mRNA delivery. *Nat. Rev. Mater.* **6**, 1078–1094 (2021). doi:10.1038/s41578-021-00358-0 Medline

62. N. Boehnke, J. P. Straehla, H. C. Safford, M. Kocak, M. G. Rees, M. Ronan, D. Rosenberg, C. H. Adelmann, R. R. Chivukula, N. Nabar, A. G. Berger, N. G. Lamson, J. H. Cheah, H. Li, J. A. Roth, A. N. Koehler, P. T. Hammond, Massively parallel pooled screening

reveals genomic determinants of nanoparticle-cell interactions. *Science* **377**, 384 (2022). doi:10.1126/science.abm5551

63. C. S. Zent, M. R. Elliott, Maxed out macs: Physiologic cell clearance as a function of macrophage phagocytic capacity. *FEBS J.* **284**, 1021–1039 (2017). doi:10.1111/febs.13961 Medline

64. A. Hakkim, B. G. Fürnrohr, K. Amann, B. Laube, U. A. Abed, V. Brinkmann, M. Herrmann, R. E. Voll, A. Zychlinsky, Impairment of neutrophil extracellular trap degradation is associated with lupus nephritis. *Proc. Natl. Acad. Sci. U.S.A.* **107**, 9813–9818 (2010). doi:10.1073/pnas.0909927107 Medline

65. J. A. Chen, S. Meister, V. Urbonaviciute, F. Rödel, S. Wilhelm, J. R. Kalden, K. Manger, R. E. Voll, Sensitive detection of plasma/serum DNA in patients with systemic lupus erythematosus. *Autoimmunity* **40**, 307–310 (2007). doi:10.1080/08916930701356317 Medline

66. D. A. Isenberg, J. J. Manson, M. R. Ehrenstein, A. Rahman, Fifty years of anti-ds DNA antibodies: Are we approaching journey's end? *Rheumatology (Oxford)* **46**, 1052–1056 (2007). doi:10.1093/rheumatology/kem112 Medline

67. S. Yung, T. M. Chan, Anti-DNA antibodies in the pathogenesis of lupus nephritis—The emerging mechanisms. *Autoimmun. Rev.* **7**, 317–321 (2008). doi:10.1016/j.autrev.2007.12.001 Medline

68. K. Ohnishi, F. M. Ebling, B. Mitchell, R. R. Singh, B. H. Hahn, B. P. Tsao, Comparison of pathogenic and non-pathogenic murine antibodies to DNA: Antigen binding and structural characteristics. *Int. Immunol.* **6**, 817–830 (1994). doi:10.1093/intimm/6.6.817 Medline

69. K. A. Fenton, B. Tømmerås, T. N. Marion, O. P. Rekvig, Pure anti-dsDNA mAbs need chromatin structures to promote glomerular mesangial deposits in BALB/c mice. *Autoimmunity* **43**, 179–188 (2010). doi:10.3109/08916930903305633 Medline

70. S. G. Thakku, J. Lirette, K. Murugesan, J. Chen, G. Theron, N. Banaei, P. C. Blainey, J. Gomez, S. Y. Wong, D. T. Hung, Genome-wide tiled detection of circulating Mycobacterium tuberculosis cell-free DNA using Cas13. *Nat. Commun.* **14**, 1803 (2023). doi:10.1038/s41467-023-37183-8 Medline

71. T. M. Soelter, J. H. Whitlock, A. S. Williams, A. A. Hardigan, B. N. Lasseigne, Nucleic acid liquid biopsies in Alzheimer's disease: Current state, challenges, and opportunities. *Heliyon* **8**, e09239 (2022). doi:10.1016/j.heliyon.2022.e09239 Medline

72. I. A. Polina, D. V. Ilatovskaya, K. Y. DeLeon-Pennell, Cell free DNA as a diagnostic and prognostic marker for cardiovascular diseases. *Clin. Chim. Acta* **503**, 145–150 (2020). doi:10.1016/j.cca.2020.01.013 Medline

73. P. Bankhead, M. B. Loughrey, J. A. Fernández, Y. Dombrowski, D. G. McArt, P. D. Dunne, S. McQuaid, R. T. Gray, L. J. Murray, H. G. Coleman, J. A. James, M. Salto-Tellez, P. W. Hamilton, QuPath: Open source software for digital pathology image analysis. *Sci. Rep.* **7**, 16878 (2017). doi:10.1038/s41598-017-17204-5 Medline

74. P. Sharma, F. Yan, V. A. Doronina, H. Escuin-Ordinas, M. D. Ryan, J. D. Brown, 2A peptides provide distinct solutions to driving stop-carry on translational recoding. *Nucleic Acids Res.* **40**, 3143–3151 (2012). doi:10.1093/nar/gkr1176 Medline

75. J. H. Kim, S.-R. Lee, L.-H. Li, H.-J. Park, J.-H. Park, K. Y. Lee, M.-K. Kim, B. A. Shin, S.-Y. Choi, High cleavage efficiency of a 2A peptide derived from porcine teschovirus-1 in human cell lines, zebrafish and mice. *PLOS ONE* **6**, e18556 (2011). doi:10.1371/journal.pone.0018556 Medline

76. S. Tabrizi, priming agents, Zenodo (2023); https://doi.org/10.5281/zenodo.10237042.

77. R. Sadeh, I. Sharkia, G. Fialkoff, A. Rahat, J. Gutin, A. Chappleboim, M. Nitzan, I. Fox-Fisher, D. Neiman, G. Meler, Z. Kamari, D. Yaish, T. Peretz, A. Hubert, J. E. Cohen, A. Salah, M. Temper, A. Grinshpun, M. Maoz, S. Abu-Gazala, A. Ben Ya'acov, E. Shteyer, R. Safadi, T. Kaplan, R. Shemer, D. Planer, E. Galun, B. Glaser, A. Zick, Y. Dor, N. Friedman, ChIP-seq of plasma cell-free nucleosomes identifies gene expression programs of the cells of origin. *Nat. Biotechnol.* **39**, 586–598 (2021). doi:10.1038/s41587-020-00775-6 Medline

78. P. Sondermann, R. Huber, V. Oosthuizen, U. Jacob, The 3.2-A crystal structure of the human IgG1 Fc fragment-Fc gammaRIII complex. *Nature* **406**, 267–273 (2000). doi:10.1038/35018508 Medline
